# Identification of extremely GC-rich micro RNAs for RT-qPCR data normalization in human plasma

**DOI:** 10.3389/fgene.2022.1058668

**Published:** 2023-01-04

**Authors:** Volker Baumann, Angelos-Theodoros Athanasiou, Omid R. Faridani, Andreas R. Schwerdtfeger, Bernard Wallner, Ralf Steinborn

**Affiliations:** ^1^ Genomics Core Facility, VetCore, University of Veterinary Medicine, Vienna, Austria; ^2^ Garvan Institute of Medical Research, Sydney, NSW, Australia; ^3^ Lowy Cancer Research Centre, School of Biomedical Sciences, University of New South Wales, Sydney, NSW, Australia; ^4^ Institute of Psychology, Karl-Franzens-University Graz, Graz, Austria; ^5^ Department of Behavioral and Cognitive Biology, University of Vienna, Vienna, Austria; ^6^ Department of Microbiology, Immunobiology and Genetics, University of Vienna, Vienna, Austria

**Keywords:** miRNA expression microarray, small-RNA sequencing, stem-loop reverse-transcription quantitative PCR, human plasma miRNAs, miRNA reference genes, cognitive stress-coping, qPCR normalization

## Abstract

We aimed at extending the repertoire of high-quality miRNA normalizers for reverse transcription-quantitative PCR (RT-qPCR) of human plasma with special emphasis on the extremely guanine-cytosine-rich portion of the miRNome. For high-throughput selection of stable candidates, microarray technology was preferred over small-RNA sequencing (sRNA-seq) since the latter underrepresented miRNAs with a guanine-cytosine (GC) content of at least 75% (*p* = 0.0002, *n* = 2). miRNA abundances measured on the microarray were ranked for consistency and uniformity using nine normalization approaches. The eleven most stable sequences included miRNAs of moderate, but also extreme GC content (45%–65%: miR-320d, miR-425-5p, miR-185-5p, miR-486-5p; 80%–95%: miR-1915-3p, miR-3656-5p, miR-3665-5p, miR-3960-5p, miR-4488-5p, miR-4497 and miR-4787-5p). In contrast, the seven extremely GC-rich miRNAs were not found in the two plasma miRNomes screened by sRNA-seq. Stem-loop RT-qPCR was employed for stability verification in 32 plasma samples of healthy male Caucasians (age range: 18–55 years). The lowest inter-individual variance of miRNA abundance was determined for miR-3665 and miR-1915-3p [coefficient of variation (*CV*) values: 0.08 and 0.50, respectively]. The eight most stable sequences included four extremely GC-rich miRNAs (miR-1915-3p, miR-3665, miR-4787-5p and miR-4497). The best-performing duo normalization factor (*NF*) for the condition of human plasma, miR-320d and miR-4787-5p, also included a GC-extreme miRNA. In summary, the identification of extremely guanine-cytosine-rich plasma normalizers will help to increase accuracy of PCR-based miRNA quantification, thus raise the potential that miRNAs become markers for psychological stress reactions or early and precise diagnosis of clinical phenotypes. The novel miRNAs might also be useful for orthologous contexts considering their conservation in related animal genomes.

## Introduction

miRNAs are non-coding, post-transcriptional regulators of gene expression. They are rather uniform in size (18–25 nucleotides (Sen, 2014)), but differ vastly in GC content, base and backbone modifications (reviewed in (Bryzgunova et al., 2021)) as well as in expression depending on time and tissue (Diener et al., 2022). Out of the 2,300 mature miRNAs extrapolated for human cells (Alles et al., 2019), a significant fraction appears to be highly conserved in other animals (Penso-Dolfin et al., 2018). miRNAs fine-tune a broad diversity of molecular functions and processes by post-transcriptional repression of mRNAs and other transcripts (Bartel, 2018) or by exerting unconventional regulatory functions outside of this paradigm (Dragomir et al., 2018). Extracellular miRNAs such as body-fluid miRNAs widely vary in half-life, but are highly stable if associated with extracellular vesicles (Coenen-Stass et al., 2019). Pronounced resistance against degradation can be provided by complexing with proteins, lipoproteins, supramolecular complexes, and by packaging in membrane-free particles such as high-density lipoprotein particles (Vickers et al., 2011) or membranous extracellular vesicles (EVs) that comprise exosomes (Bryzgunova et al., 2021), shedding microvesicles (Hunter et al., 2008), and apoptotic bodies (Zernecke et al., 2009). These “shields” are likely responsible for the remarkable stability of cell-free miRNAs over prolonged storage at room temperature and multiple freeze-thaw cycles (Chen et al., 2008; Mitchell et al., 2008; Zampetaki et al., 2012; Rao et al., 2013). The half-life of extracellular mature miRNAs positively correlates with GC content (Coenen-Stass et al., 2019). Moreover, when a mature miRNA is “free”, not knotted into the RISC complex, folding into hairpin secondary structures can convey nuclease resistance (Belter et al., 2014). Features such as non-invasiveness, stability, and repeatability, combined with the ability of EV-shuttled miRNAs to cross the blood-brain barrier (Banks et al., 2020), makes circulating plasma/serum miRNAs an ideal diagnostic signature for brain-related contexts. These phenotypes range from cancer (Dai et al., 2020), stroke (Wang et al., 2018), neurodegenerative disorders (Wang and Zhang, 2020) and their early stages (Sheinerman et al., 2012) over depression (Chen et al., 2020; Li et al., 2021) up to stress and stress-associated diseases (Crigna et al., 2020).

A miRNA can increase its impact by targeting a set of genes that are involved in the same pathway or protein complex (Linsley et al., 2007), hence even relatively small changes of its abundance can cause an amplified biological effect (Calin and Croce, 2006). From the point of view of accurate miRNA quantification, intolerance towards poor normalization is especially important since alteration of miRNA abundance tends to be subtle, typically a few fold (Zhang et al., 2012). Technical noise reduction of qPCR-based measurement of a particular miRNA would be essential and can be leveraged by up-to-date data normalization with two to three stable endogenous controls appropriately selected for the experimental context of interest (Pagacz et al., 2020).

The identification of miRNAs that are stably abundant in human plasma, serum and extracellular vesicles was a research topic for more than a decade (Kroh et al., 2010; Meyer et al., 2010) up to the present (Faraldi et al., 2018). At the onset of the qPCR-based era of quantitative miRNA analysis, small nucleolar RNAs or small nuclear RNAs were frequently used for normalization (Song et al., 2012; Dufourd et al., 2019). Considering their low abundance in body fluid samples (Mitchell et al., 2008; Turchinovich et al., 2011; Qi et al., 2012; Zhang et al., 2016), higher variability (Wotschofsky et al., 2011; Schwarzenbach et al., 2015) and different class assignation, they were soon replaced by miRNA spike-ins (Roberts et al., 2014) and/or specific miRNAs (Zhang et al., 2016; Hakansson et al., 2018; Faraldi et al., 2019). Favourably, the normalizer in low-throughput profiling of extracellular miRNAs should come from the same molecule class, should be amenable to miRNA assay design, exhibit similar extraction efficiency, degradation and turnover regulation (Zhang et al., 2012) and shuttling. There are several layers requiring attention in the identification process of normalizers. Their extraction can be biased across samples and studies resulting from different extraction chemistries (Tan et al., 2015; Parker et al., 2021). The comparison of miRNA stability across different cohorts can be hampered by various physiological parameters impairing their abundance such as subject’s age (Noren Hooten et al., 2013; Ameling et al., 2015; Fehlmann et al., 2020; Maffioletti et al., 2020; Wang et al., 2020), gender (Duttagupta et al., 2011; Ameling et al., 2015), pregnancy (Tay et al., 2017) or ethnicity (Patel et al., 2019). Moreover, levels of circulating miRNAs can be impacted by alcohol consumption (Ignacio et al., 2015), dietary habit (Tarallo et al., 2014; Liu et al., 2020), poor sleep quality (Baek et al., 2021), obesity parameters (body mass index (Ameling et al., 2015), levels of visceral and subcutaneous adipose tissue, or body-fat percentage (Munetsuna et al., 2018)), settlement (*e.g*. high-altitude hypoxic environment (Yan et al., 2015)), psychological activity (Dal Lin et al., 2021), exercise (Wardle et al., 2015; Li et al., 2020) including training regime (Wardle et al., 2015) and cigarette smoking (Takahashi et al., 2013; Banerjee et al., 2015). The latter deregulated nine of the miRNA normalizers formerly recommended, namely let-7b, miR-16, miR-93-5p, miR-126-3p, miR-185, miR-188-5p, miR-191, miR-345 and miR-425-5p (Takahashi et al., 2013), thus representing a striking example for the importance of physiological context.

There are various methodologies for the selection of miRNA normalizers (Mathew et al., 2020). When choosing the profiling platform for undirected selection of stable miRNAs, the specific strengths and weaknesses of a technique have to be considered together with the experimental aim and setting (Mestdagh et al., 2014). For example, extreme base compositions of DNA or RNA such as AT- or GC-rich sequences, have caused an uneven coverage or even no coverage of reads on Illumina’s platform for next-generation sequencing (NGS) (Aird et al., 2011; Chen et al., 2013; Backes et al., 2016).

Aiming at the selection of high-quality normalizers for human plasma, our study considered the positive correlation between half-life and GC content of intra- and extracellular miRNAs (Kakimoto et al., 2016; Coenen-Stass et al., 2019). For high-throughput selection of stable miRNAs we preferred microarray analysis over NGS to avoid bias against highly GC-rich miRNAs. We also aimed at inspiring future research on miRNA markers related to psychological stress reactions by devising normalizers for the condition of cognitive stress-coping styles self-assessed using the Mainz Coping Inventory (MCI).

## Materials and methods

### Study design and participants

For microarray-based selection of stable miRNA normalizers (phase 1) and their verification by qPCR (phase 2), separate sets of male Caucasians were recruited. Grouping of participants in the two study phases was based on the dimensions of cognitive avoidance and vigilance scored by answers to the MCI self-report instrument. This theory-based, self-administered stimulus-response inventory is based on the participant’s stated behaviour in hypothetical physical or self-esteem harming situations (Krohne and Egloff, 1999). The model postulates four characteristic modes of coping with threat, sensitization (low cognitive avoidance and high vigilance), repression (high avoidance, low vigilance), anxiety (high scores on both dimensions), and non-defensiveness (low scores on both dimensions). Completed MCI surveys were evaluated using the statistical software platform IBM^®^ SPSS^®^ Statistics version 20 (SPSS Inc, Chicago, IL, United States of America).

In phase 1, a participant was assigned to one of the four MCI groups applying a threshold of ± 2 of the median scores for coping dimensions of cognitive avoidance and vigilance calculated for the cohort of all study participants (*n* = 64). Considering this “spacing zone”, blood donors of study phase-1 were selected when exhibiting score ranges of cognitive avoidance and vigilance of ≤23 to ≥27 and ≤16 to ≥20, respectively. To meet the required threshold of total RNA input requested by the manufacturer of the miRNA expression microarray, sample pooling had to be performed occasionally (Supplementary Table S1).

In phase 2, eight probands were selected per coping group without considering a “spacing zone” (*n* = 32; age range: 18–55 years; Supplementary Table S2).

Plasma RNAs subjected to sRNA-seq were derived from a proband that was also listed as participant of study phase-2 and from an independent adult male of Caucasian decent.

### Plasma preparation

Participants reporting to be in good physical health donated ∼9 mL of venous blood that was collected into EDTA tubes (Vacuette^®^ K2EDTA tubes; Greiner BioOne International AG, Kremsmünster, Austria). To exclude regulation of miRNA expression by the circadian rhythm (Shende et al., 2011), blood was taken around the same day time (between 8 and 11 a.m.). Cells and cellular debris were pelleted within 30 min after blood donation (3,000 × *g*, 10 min, room temperature). Aliquots of the supernatant were stored at −80°C until RNA extraction.

### Extraction of plasma RNA

Efficiency of RNA extraction, synthesis of complementary DNA (cDNA) and PCR amplification was monitored using a synthetic spike-in control (McAlexander et al., 2013; Tan et al., 2015). It represented a miRNA-like sequence termed “spike-A” (Androvic et al., 2019) that was predicted to be linear. RNA was extracted using silica-membrane spin-column technology (Qiagen, Hilden, Germany) supplied by the miRNeasy Serum/Plasma Kit (phase 1) or the miRNeasy Serum/Plasma Advanced Kit (phase 2). In phase 2, 2 μg of glycogen carrier (Thermo Fisher Scientific GmbH, Vienna, Austria) was added per 200 μl plasma for improving recovery and reproducibility (Androvic et al., 2019). An aliquot of 200 μl plasma thawed on ice was mixed with lysis solution containing 2.0 × 10^−5^ spike copies. The concentration of the RNA extracted was slightly increased by reducing the volume of the eluant (45 μl instead of 50 μl RNase-free water). To exclude elevated levels of free oxyhaemoglobin stemming from sample haemolysis, spectrophotometric absorbance was measured at 414 nm applying a threshold of less than 0.2 (Kirschner et al., 2011).

Body-fluid RNA can rarely be assessed for integrity based on an RNA integrity number (RIN) value, as the RNA concentration is usually too low. When these samples have a high enough RNA concentration to detect a RIN value, it would be low. This is due to the fact that plasma/serum samples would contain short fragments of RNA (<1,000 nucleotides) being perceived by the microfluidic-measurement system as degraded RNA. Usually, ribosomal RNAs are not detected in these samples (Lam et al., 2012). Since RNA concentration extracted from plasma/serum volumes of ≤200 μl is at the limit of accurate quantification by spectrophotometric or microfluidic measurement (Schlosser et al., 2015; Marabita et al., 2016), normalization based on a fixed amount of starting RNA was considered impracticable and replaced by normalization to a fixed volume of template (Marabita et al., 2016). RNA was stored at −80°C until use.

### sRNA-seq analysis

Small-RNA libraries were prepared without experimental size-selection (Faridani et al., 2016). The protocol included the use of unique molecular identifiers (UMIs) to counteract PCR stochasticity and to enable counting of RNA molecules. Briefly, a 3′ adaptor was ligated to total RNA using T4 RNA Ligase 2, truncated KQ (New England Biolabs, Ipswich, MA, United States). Then, free 3′ adaptors were removed enzymatically using 5′ deadenylase and Lambda exonuclease (New England Biolabs). Next, the 5′ adaptor containing a UMI was ligated using T4 RNA ligase 1 (New England Biolabs). The use of UMIs was reported to mitigate RT and amplification biases, hence to improve accuracy and reliability of miRNA quantification by sRNA-seq (Wright et al., 2019). Note that only sRNAs can be ligated to 5′ adaptor and mRNA ligation is inhibited due to the existence of the cap structure. RT was performed using Invitrogen™ SuperScript™ II Reverse Transcriptase (Thermo Fisher Scientific) followed by PCR using indexed primers and KAPA HiFi polymerase (Roche Diagnostics International AG, Rotkreuz, Switzerland). Final cDNA libraries were cleaned by AMPure XP beads (Beckman Coulter AB, Bromma, Sweden), checked by Bioanalyzer High Sensitivity DNA Kit (Agilent Technologies Sweden AB, Sundbyberg, Sweden) and subjected to NGS.

Sequencing-by-synthesis and mapping of reads were performed by the Core Facility for Next-Generation Sequencing of the University of Vienna (www.viennabiocenter.org/vbcf/next-generation-sequencing, part of Vienna BioCenter Core Facilities, Austria). Sequencing was performed on the HiSeq 2500 System (Illumina Inc, San Diego, CA, United States) using SR50 for a read length of 50 bp and the High Output Run Mode (v4). The computational pipeline used for read annotation (Faridani et al., 2016) is available at https://github.com/eyay/smallseq. First, the 8-bp unique molecular identifiers were removed from the 5′ end of the raw reads using scripts of the Computational Genomics Analysis Tools (CGAT) collection https://cgat.readthedocs.io/en/latest/cgat.html. Second, adapters and the CA spacer preceding each read were removed using the adapter trimmer Cutadapt (Martin, 2011). UMI sequences were added to the header of the reads for the ease of later processing. Third, reads were mapped against the human reference genome (ncbi38_hg20) using the Spliced Transcripts Alignment to a Reference (STAR) aligner ((Dobin et al., 2013); https://github.com/alexdobin/STAR/releases; version 2.7.3a) run with the settings outSAMstrandField: intronMotif, outFilterMultimapNmax: 50, outFilterScoreMinOverLread: 0, outFilterMatchNmin: 18, outFilterMatchNminOverLread: 0, and outFilterMismatchNoverLmax: 0.04, alignIntronMax: 1. This disabled spliced alignment and mismatching within the first 25 bp of the read while allowing a single mismatch in total. PCR duplicates were removed by deduplicating and counting the sequences using UMI tools (https://github.com/CGATOxford/UMI-tools), thereby collapsing the reads based on the adjacency network approach. In the last step, the biotypes of the remaining reads were assigned based on the annotation of mirBase (www.mirbase.org; version 21), the Genomic tRNA Database (GtRNAdb; (Chan and Lowe, 2016); http://gtrnadb.ucsc.edu) and the GENCODE project (www.gencodegenes.org; release 22). Sequence molecules mapping to multiple genomic locations were assigned by a weighting approach (molecule number/number of annotated locations). miRNAs expressed from different genomic locations were collapsed.

### miRNA profiling with expression microarray

Plasma abundance of human miRNAs was measured on the GeneChip^®^ miRNA 3.0 Array (Thermo Fisher Scientific; formerly Affymetrix, Santa Clara, CA, United States) that requires a minimum input of 130 ng RNA according to the manufacturer. Three biological replicates were measured per coping mode using a template amount of 176 ng RNA. To overcome limitations in sample quantity and to reduce variance, template RNA was pooled from three to eight individuals (sample set 1, Supplementary Table S1). Microarray expression analysis was outsourced to a commercial service provider (AROS Applied Biotechniques A/S, Aarhus, Denmark, www.arosab.com; part of Eurofins Genomics, https://eurofinsgenomics.eu/) and analysed as follows. Two unimodal signal distributions, background noise caused by false-positive hybridization and the actual signal of an expressed miRNA, and their superimposition were observed in the signal number per intensity plot. To separate the two signal modes, a cut-off was manually set and only miRNAs expressed above this threshold were further analysed. Targets not called present by the Detection Above Average (DABG) algorithm implemented by Affymetrix were excluded. Consistency of miRNA expression was evaluated following normalization with nine approaches (global mean normalization, generalized procrustean analysis, loess, cyclic loess, modified loess, quantile normalization, variance stabilization normalization, variance stabilization normalization based on invariant miRNAs, and Z-score normalization). If not implemented in a normalization workflow, background correction was performed by applying the R function “bg.correct.rma” included in the R package ‘affy’ (Gautier et al., 2004). For each expressed miRNA, the occurrences of top-ten ranks across the nine normalization approaches were counted. Consistency of abundance was assumed if a miRNA was called present on at least eleven of the twelve arrays. To rank the stabilities of the consistently expressed miRNA set, their signal intensity values were log_2_ transformed and treated similarly to cycle-of-quantification (*Cq*) values of RT-qPCR. Expression stability was assessed with four common statistical algorithms, geNorm (Vandesompele et al., 2002), Normfinder (Andersen et al., 2004), BestKeeper (Pfaffl et al., 2004), and the Δ*Ct* method (Silver et al., 2006) accessed at the comprehensive platform RefFinder ((Xie et al., 2012); www.heartcure.com.au/reffinder/). The ranks of the resulting four lists were aggregated using the R package “RankAggreg” (Pihur et al., 2009) that assigns an appropriate weight to an individual gene and calculates the geometric mean of their weights for the overall final ranking.

### Design of stem-loop RT-qPCR oligonucleotides

Specificity of the stem-loop RT-qPCR assays was achieved by the stem-loop RT primer and the forward PCR primer except of the assay against miR-93-5p. In this case, specificity was derived solely from a specific 3′ end of the forward PCR primer (Supplementary Table S3). Assays used two versions of a universal reverse amplification primer and dye-based monitoring of product accumulation.

Considering that *in silico* tools such as Primer-BLAST ((Ye et al., 2012); https://www.ncbi.nlm.nih.gov/tools/primer-blast/) or Human BLAT Search (Kent, 2002); https://genome.ucsc.edu/cgi-bin/hgBlat) could not easily be adapted to predict the specificity of oligonucleotides designed for miRNA detection, *e.g.* of those employed by stem-loop RT-qPCR, their specificity was evaluated as follows. FASTA-formatted, 5′ to 3′ sequences of all mature human miRNAs were downloaded from miRBase (release 22 March 2018 (Kozomara and Griffiths-Jones, 2014); https://www.mirbase.org) and saved in a Microsoft Excel spreadsheet. The 5′ to 3′ sequences and their reverse counterparts were sorted alphabetically to facilitate assessment of the cross-hybridization potential of the forward PCR primer and the stem-loop RT primer, respectively (Supplementary Table S3). Based on this alignment, for certain extremely GC-rich miRNAs the common single-stranded specific stretch of six-nucleotide at the 3′ end of the stem-loop RT primer (Varkonyi-Gasic and Hellens, 2011) had to be extended by one to three nucleotides.

All stem-loop RT primers were designed for optional application of a universal short hydrolysis probe modified with locked nucleic acid (LNA) bases (*Tm*: ∼70°C; https://geneglobe.qiagen.com/us/tools/tm-prediction). In the 8-mer probe sequence 5′-T+G+G+C+T+C+T+G all nucleotides except the last at the 5′ terminus were LNA-modified. This was to allow cleavage by the 5′ to 3′ nuclease activity of *Taq* DNA polymerase (Echwald et al., 2013).

### Quantification of mature miRNAs by dye-based stem-loop RT-qPCR

Plasma abundance of mature miRNAs was quantified using stem-loop RT-qPCR that discriminates between mature and pre- or pri-miRNA by 100-fold (Kramer, 2011). Before stem-loop RT-qPCR, the structure of the stem-loop RT primer was re-constituted by denaturing at 95°C for 10 min and reassociation achieved by reducing the temperature to 75°C slowly at a speed of 1°C/s, 1 h incubations at 75, 68, 65 and 62°C, and incubation at 60°C for several hours (Kramer, 2011). For pre-RT annealing, 2 μl template RNA was mixed with 1 *μ*l of 1 *μ*M RT primer and 3.25 μl RNase-free water, and heated to 65°C for 1 min to remove secondary structures. After the annealing step performed for 30 min at the specific temperature requested by the particular RT primer (24°C–45°C), the mixture was immediately placed on ice. Subsequently, it was supplemented with 1 mM dNTP mix (Solis BioDyne, Tartu, Estonia), 1 × RT-Buffer, 10 U RiboLock RNase Inhibitor and 100 U RevertAid Reverse Transcriptase (all Thermo Fisher Scientific) to obtain a final RT volume of 10 *μ*l. Following pulsed RT (60 cycles: 30°C for 30 s, 42°C for 30 s, 50°C for 2 s) performed to minimise unspecific interaction and increase detection sensitivity (Chen et al., 2005), cDNA synthesis was terminated by enzyme inactivation at 85°C for 5 min. Incubation steps of RT were performed on a MJ Research PTC-200 Thermal Cycler (Bio-Rad, Hercules, CA, United States). To monitor the impact of putative DNA contamination, a mock-RT reaction was carried out for each experimental RNA.

The signal of stem-loop RT-qPCR was generated using the SYTO™ 82 Orange Fluorescent Nucleic Acid Stain (Thermo Fisher Scientific). It produces a signal-to-noise ratio of ∼40 (https://www.gene-quantification.de/syto-dyes.pdf) and a narrow peak during amplicon melting (Gudnason et al., 2007), is without AT- or GC preference (Giglio et al., 2003; Gudnason et al., 2007) and not inhibitory to the PCR (Gudnason et al., 2007), binds DNA mainly dependent on charge and primarily in the minor groove (Eischeid, 2011), and exhibits lower nucleic-acid affinity compared to the common SYBR Green I (Eischeid, 2011), hence, has less potential to form primer hybrids (Gudnason et al., 2007).

The 15-*μ*l qPCR contained 1.2 mM dye, 0.2 μM of each primer (Sigma-Aldrich, St. Louis, MO, United States), 1 × B2 buffer (Tris-HCl (NH_4_)_2_SO_4_ and Tween 20), 0.2 mM of each dNTP, 2.5 or 3.0 mM MgCl_2_ depending on assay, 1 U of the hot-start *Taq* DNA polymerase HOT FIREPol^®^ (all Solis BioDyne) and 4 µl cDNA that was eight-fold diluted in 1 × TE buffer (10 mM Tris-HCl, 1 mM EDTA, pH 8). Amplification reactions were carried out in a 96 well-plate format and scored using the LightCycler^®^ 96 Instrument operated by the LightCycler^®^ 96 application version 1.1.0.1320 (Roche Diagnostics GmbH, Vienna, Austria). Following template denaturation and activation of the polymerase at 95°C for 15 min, amplification was performed over 40 cycles (denaturation for 20 s at 95°C, annealing for 20 s at 59°C, 60°C, 61°C or 62°C, and elongation for 10 s at 72°C). Fluorescence was monitored during the extension step. The specificity of each primer set was verified by melt-curve analysis. Following initial denaturation at 95°C for 10 s and renaturation at 65°C for 60 s, amplicons were dissociated by ramping from 65 to 97°C in 0.2°C increments per second. Fluorescence signals were continuously acquired at each increment.

### S-poly(T) miRNA method for quantification of miR-1469

The S-Poly(T) miRNA method (Kang et al., 2012) comprises miRNA polyadenylation and RT with a S-Poly(T) primer that contains sites for binding of a universal reverse PCR primer and a universal probe, of an oligo (dT)_11_ sequence and of several miRNA-specific bases at its 3′ end. RT of miR-1469 was primed with 5′-CAG​TGG​AGG​GTC​GGA​GGT​CAGTGG​CTC​TGTTT​TTT​TTT​TTGGA​GCC, where the underlined nucleotides are target specific. The 8-mer presented in bold designates the arbitrary sequence of the hydrolysis probe 5′-T+G+G+C+T+C+T+G used to monitor amplification (see above). Amplification was primed with the universal reverse primer 5′-CAG​TGG​AGG​GTC​GGA​GGT and the specific forward primer 5′-ata​att​taC​TCG​GCG​CGG​G, where lower-case letters designate the arbitrary *Tm*-enhancing tail.

### Efficiency of qPCR amplification, adjustment of measured *Cq* values and outlier treatment

Amplification efficiency was calculated from the exponential phase of the amplification curve. In detail, not baseline-corrected (raw) fluorescence values exported from the qPCR system’s operating software in the Real-time PCR Data Markup Language were imported into Real-time PCR Miner 4.0 ((Zhao and Fernald, 2005); http://miner.ewindup.cn/miner/) run under default settings. *Cq* values measured at *E*_*fi*_ < 1 were corrected according to the term *Cq* × log_10_ (*E*_*fi*_ + 1)/log_10_2) (Kubista et al., 2007), where *E*_*fi*_ represents the mean of the individual amplification efficiencies determined for the assayed cDNAs.

A qPCR replicate was treated as outlier and discarded when it caused a standard deviation of more than 0.5 calculated for the quadruplicate *Cq* values of an experimental sample (qPCR duplicates for each of the two cDNA replicates). If the means of the qPCR duplicates run for each cDNA replicates differed by more than one cycle, the cDNA replicate that exhibited the highest deviation from the global average of the 32-sample cohort was removed from analysis.

### Assessment of miRNA abundance stability

#### *CV* analysis

The *CV* value (ratio of standard deviation to the mean) was used as a quality control metrics reflecting the variation of a reference gene across all samples. It was used as the simplest parameter to compare the variation of the sequence abundances independent from their actual levels (Boda et al., 2009).

#### Efficiency adjustment of *Cq* values

Efficiency-adjusted, not log-transformed *Cq* values were used as input to determine the optimal number of reference sequences for appropriate RT-qPCR normalization and to rank the expression stability of single miRNAs and duos or trios of miRNAs.

#### Determination of the optimal number of sequences for RT-qPCR normalization

The optimal number of miRNAs required for appropriate normalization was concluded based on the pairwise variation *V* value of the geNorm tool. The software determined the optimal number of normalizers based on pairwise variation (*V*_*n*_ */V*_*n+1*_) between two sequential factors, *NF*_*n*_ and *NF*_*n+1*_. The common cut-off value of *V*_*n*_ */V*_*n+1*_ < 0.15 determined the lowest number of genes for accurate normalisation (Vandesompele et al., 2002).

#### Abundance stability of single normalizers

The stability of single miRNAs across the four stress-coping modes was assessed using the statistical algorithms, geNorm (Vandesompele et al., 2002; Hellemans et al., 2007) incorporated in the qbase + software 3.2 (Biogazelle, Ghent, Belgium; (Hellemans et al., 2007); www.qbaseplus.com), and the Excel-based programs NormFinder (Andersen et al., 2004) and BestKeeper (Pfaffl et al., 2004). The resulting three rank scores were compiled into a comprehensive rank using the ComprFinder algorithm (Zhang et al., 2020). In contrast to the geometric averaging applied by the RefFinder platform, ComprFinder maintains the dimension of an individual stability gain or loss by standardising the range of the stability score across the individual statistical algorithms.

#### Systematic assessment of normalizer combinations

The abundance stability of all possible *NF*s calculated as the geometric mean of two or three miRNAs, was compared using NormiRazor ((Grabia et al., 2020); https://norm.btm.umed.pl), a tool that includes the geNorm, NormFinder and BestKeeper algorithms.

### Statistical analysis

Statistical analysis was performed using GraphPad Prism 9.2.0 software (GraphPad Software Inc, San Diego, CA, United States), Microsoft Excel 2010 (Microsoft Corporation, Redmond, United States) or The R Project for Statistical Computing (https://www.r-project.org) if not otherwise stated.

Pearson’s correlation between the two best-ranked miRNA normalizers for the condition of plasma irrespective of copying style, of their efficiency-adjusted *Cq* values was assessed at https://astatsa.com/CorrelationTest/using default settings (two-sided, true correlation: non-zero).

The *CV* value (relative standard deviation) was used to reflect the extent of variability in miRNA abundance. Pearson’s correlation between miRNA’s *CV* and minimal free energy (*MFE*) values was calculated using the Correlation Coefficient Calculator (Statistics Kingdom, Melbourne, Australia; https://www.statskingdom.com/correlation-calculator.html).

*MFE* of folding calculated for miRNA sets with moderate or extremely high GC contents (below or above 64%) was evaluated for statistical significance using the Mann-Whitney *U* test Calculator with default settings (https://www.statskingdom.com/170median_mann_whitney.html).

Normality of miRNA’s *Cq* values reflecting their plasma abundances was examined by the Shapiro-Wilk test. Since data were not always normally distributed, the non-parametric Mann-Whitney *U* test was used to assess differential abundance of a miRNA across a stress-coping dimension (low versus high scores of cognitive avoidance or low versus high vigilance).

## Results

Nowadays, stably expressed miRNAs can be selected from miRNomes by various approaches including RNA-seq and the historically older microarray technology. Due to its low amount of RNA, profiling plasma by RNA-seq would involve an exponential amplification step during construction of the sequencing library. However, common protocols for NGS cause under-representation of highly GC-rich sequences owing to inefficient amplification by PCR. Therefore, we asked whether miRNA profiling by sRNA-seq that does not involve measures against GC-rich sequence bias (Faridani et al., 2016) would similarly be challenged. Expression data measured on a miRNA microarray platform were used for comparison.

### Bias of small RNA-seq against extremely GC-rich miRNAs

**FIGURE 1:**
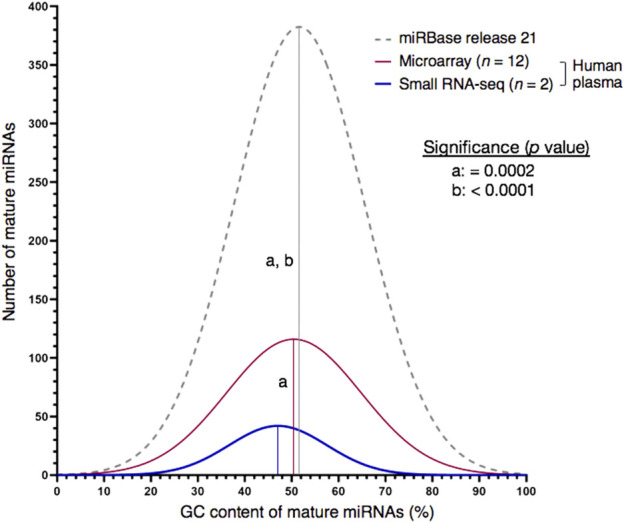
Choice of high-throughput profiling technique may cause detection bias for miRNA targets with very high GC content (≥75%). Whereas the GC plot of miRNAs contained in the miRBase database was similar with the one of miRNAs detected by miRNA microarray analysis on the Affymetrix GeneChip^®^ platform, small RNA-seq produced a left-skewed distribution as indicated by Shapiro-Wilk test for normality. Numbers of miRNAs annotated by miRBase database or detected by expression analysis in blood plasma of human males: 2,654 (miRBase, release 22), 837 (miRNA microarray, *n* = 12) and 226 (sRNA-seq, *n* = 2). Mean GC proportions are depicted by bell-shaped curves: miRBase database (grey), miRNA microarray (red) and sRNA-seq (blue). Non-significant and significant differences between two means are depicted by the same or a different letter, respectively. Significance was analysed with Kruskal–Wallis test followed by Dunn’s test using a threshold of *p* < 0.01.

GC bias is inherent to any PCR-amplified RNA-seq library preparation (Shi et al., 2021; Santacruz et al., 2022). For example, transcript regions with a GC content above 70% can strongly be underrepresented (Sendler et al., 2011). Here, we asked whether this limited ability to capture the true sequence representation would also affect our sRNA-seq workflow. Exemplarily, we profiled two plasma samples from probands exhibiting low or high MCI scores for vigilance. While both the set of mature human miRNAs contained in the miRBase repository and the two NGS-derived plasma miRNomes did not follow a normal distribution (*p* < 0.001, Supplementary Table S4), the sRNA-seq workflow was biased from false-negative detection of extremely GC-rich miRNAs (≥75% G/C, *p* = 0.0002, Figure 1 and Supplementary Table S5). Therefore, microarray expression analysis was subsequently employed to select stable miRNAs at high throughput.

### Selection of stable miRNAs from microarray expression data

When profiling human plasma for miRNAs, it is important to remember that it contains traces of bacterial and viral RNA in addition to miRNAs and other transcripts of the host (Semenov et al., 2012). The miRNA microarray data produced for our human plasma samples contained a high fraction of miRNAs with low expression (Supplementary Figure S1). We argued that the bimodal distribution observed in the signal number per signal intensity plot is caused by a mixture of two unimodal distributions with either high or low signal intensities. Whereas the former reflects the distribution of actually expressed miRNAs, the latter is derived from extremely low expressed or even unexpressed miRNAs as well as from artefacts due to cross hybridization of various RNA fragments (Semenov et al., 2012) or other background noise. The exclusion of low-intensity signals resulted in a list of 103 mature human miRNAs (Supplementary Table S6). Constitutive expression across the experimental samples was assumed if a miRNA could be detected on at least eleven of the twelve arrays evaluated by the DABG algorithm. This request was supposed to compensate for possible cases of false-negative detection by the algorithm. A set of 37 mature miRNAs passing this criterion (Supplementary Table S7) were analysed by nine approaches for microarray data normalization (Supplementary Table S8). The particular effects of the normalizations on the distribution and balancing of individual hybridization intensities were illustrated in Supplementary Figure S2. Application of the nine approaches identified 24 miRNAs with at least one top-ten call (Table 1). For each of the nine normalization workflows, we assessed their expression stability using the RefFinder tool that delivered a comprehensive ranking based on the four common statistical algorithms geNorm, NormFinder, BestKeeper and Δ*Ct* method. Finally, a list of putative reference miRNAs was derived by aggregating the individual ranks (Supplementary Table S9).

**TABLE 1:** Number of top-ten ranks of consistently expressed human plasma miRNAs across nine approaches for microarray data normalization.

No.	Mature human miRNA	Rank score in microarray normalization approach									“Top-10” calls
		GMN	GPA	Loess	Loess_C	Loess_M	Quantile	VSN	VSN-INV	Z-Score	
1	miR-4488-5p	4	8	8	1	8	4	6	5	3	9
2	miR-4787-5p	3	1	1	10	1	7	>10	8	10	8
3	miR-1469	6	4	6	3	5	5	>10	6	7	8
4	miR-3665-5p	1	2	7	2	7	1	>10	2	2	8
5	miR-3960-5p	2	3	5	>10	4	9	>10	1	6	7
6	miR-1915-3p	5	10	>10	7	10	8	5	>10	5	7
7	miR-3656-5p	>10	>10	4	8	3	10	7	3	>10	6
8	miR-320d	>10	5	2	>10	2	2	>10	4	>10	5
9	miR-486-5p	8	>10	3	6	>10	>10	>10	>10	1	4
10	miR-19b	10	>10	>10	>10	>10	6	>10	9	8	4
11	miR-4466	>10	9	>10	4	>10	3	1	>10	>10	4
12	miR-425-5p	>10	6	10	>10	6	>10	9	>10	>10	4
13	miR-2861	>10	7	>10	>10	>10	>10	>10	10	9	3
14	miR-185-5p	>10	>10	>10	5	>10	>10	>10	7	>10	2
15	miR-4497-5p	>10	>10	>10	>10	9	>10	3	>10	>10	2
16	miR-320c	7	>10	>10	>10	>10	>10	>10	>10	>10	1
17	miR-4668-5p	9	>10	>10	>10	>10	>10	>10	>10	>10	1
18	miR-320b	>10	>10	>10	>10	>10	>10	>10	>10	4	1
19	miR-1281	>10	>10	>10	>10	>10	>10	2	>10	>10	1
20	miR-378h	>10	>10	>10	>10	>10	>10	4	>10	>10	1
21	miR-4487	>10	>10	>10	>10	>10	>10	8	>10	>10	1
22	miR-4763-3p	>10	>10	>10	>10	>10	>10	10	>10	>10	1
23	miR-3616-3p	>10	>10	9	>10	>10	>10	>10	>10	>10	1
24	miR-92a	>10	>10	>10	9	>10	>10	>10	>10	>10	1

Numbers represent expression stability ranks of a microarray normalization approach. Abbreviations: GMN, global mean normalization; GPA, generalized procrustean analysis; Loess, Locally estimated scatterplot smoothing; Loess_C, cycle loess; Loess_M, modified loess; VSN, variance stabilization normalization; VSN-INV, VSN based on invariant miRNAs; Z-score, Z-Score Normalization. Underlined: putative miRNA reference genes selected for validation by RT-qPCR.

List restricted to the consistently expressed miRNAs, that produced at least a single “top-10” call.

### Set-up of stem-loop RT-qPCR assays

**FIGURE 2:**
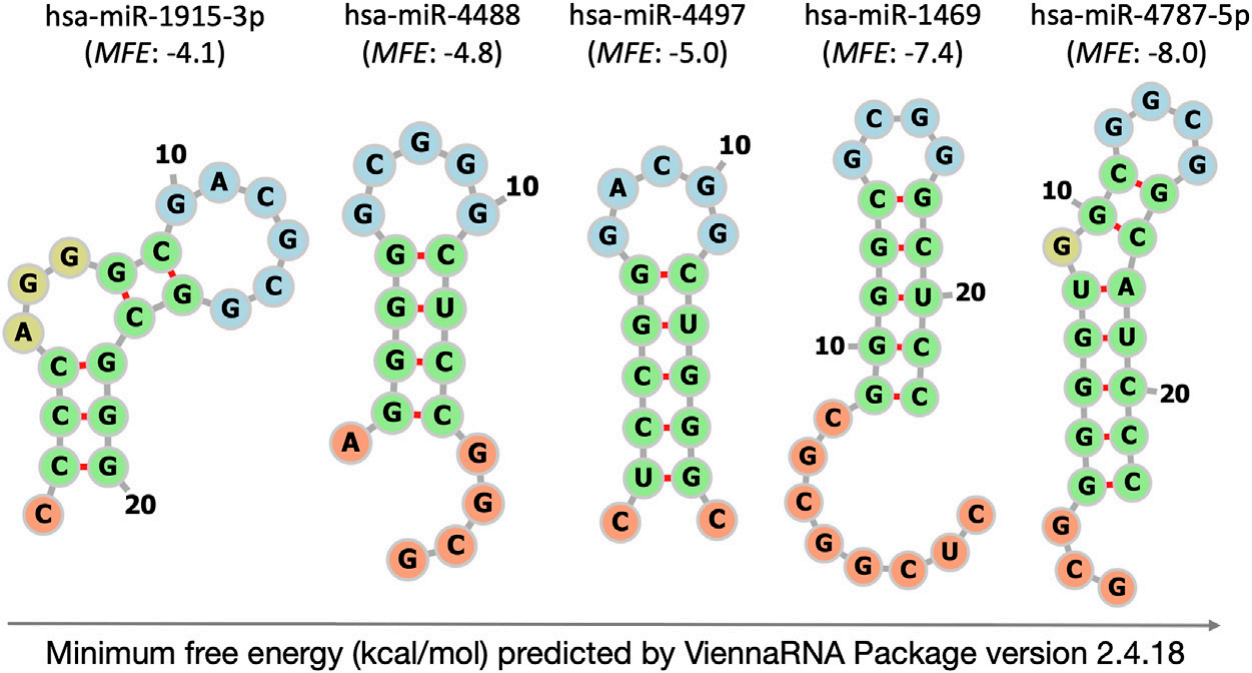
Self-complementarity of extremely GC-rich mature miRNAs at 37°C predicted by ViennaRNA Package version 2.4.18. The illustration of intramolecular hybridisation is limited to the five microarray-selected miRNAs with most negative Δ*G* values. *MFE*: minimum free energy (kcal/mol). Structural details depicted by colour are loop (blue), stem (green), single-stranded tail (orange) and bulge or internal loop (olive).

From the consensus list of the nine approaches for microarray data normalization, twelve constitutively and stably abundant miRNAs were chosen to validate their plasma stability by stem-loop RT-qPCR (Table 2). The set covered inter- and intragenic miRNAs with a wide range of chromosomal locations, GC contents and secondary structures including several well-formed hairpins with very negative *MFE* values of up to −8 kcal/mol (Supplementary Table S9 and Figure 2). Each of these normalizer candidates was (also) found to be residing within circulating exosomes of the blood (Supplementary Table S9).

**TABLE 2:** Details of stem-loop RT-qPCR assays.

Mature miRNA target^a^				Details of RT		Details of qPCR			
Mature miRNA	miRBase identity	Sequence of miRNA	GC content (%)	Sequence of stem-loop primer	*Ta* of pre-RT (°C)	Forward (F) & reverse (R) primers	*Ta* (°C)	Amplicon length (bp)	*E*
miR-93-5p	MIMAT0000093	CAAAGUGCUGUUCGUGCAGGUAG	52.7	GTGGCTCTGCAGTGCAGGGTCCGAGGTAtgCAGAGCCAcCTACCTGC	24	F:atgacagtCAAAGTGCTGTTCGT; R1	60	61	0.85
miR-126-3p	MIMAT0000445	UCGUACCGUGAGUAAUAAUGCG	45.5	GTGGCTCTGCAGTGCAGGGTCCGAGGTAtgCAGAGCCAcCGCATTATT	24	F:acgacagtTCGTACCGTGAGT; R1	59	60	0.85
miR-185-5p	MIMAT0000455	UGGAGAGAAAGGCAGUUCCUGA	50.0	cTGGCTCTGCAGTGCAGGGTCCGAGGTAagCAGAGCCAgTCAGGA	25	F:agcatctaTGGAGAGAAAGGCAG; R1	60	60	0.85
miR-320d	MIMAT0006764	AAAAGCUGGGUUGAGAGGA	47.4	GTGGCTCTGCAGTGCAGGGTCCGAGGTAtgCAGAGCCAcTCCTCTCA	24	F:tgactcacagAAAAGCTGGGT; R1	59	59	0.87
miR-425-5p	MIMAT0003393	AAUGACACGAUCACUCCCGUUGA	47.8	GTGGCTCTGCAGTGCAGGGTCCGAGGTAtgCAGAGCCAcTCAACGGG	25	F:tactcacagAATGACACGATCACT; R1	59	62	0.85
miR-486-5p	MIMAT0002177	UCCUGUACUGAGCUGCCCCGAG	63.6	gTGGCTCTGCAGTGCAGGGTCCGAGGTAtgCAGAGCCAcCTCGGG	25	F:gatcaatataTCCTGTACTGAGCTGC; R1	60	62	0.88
miR-1915-3p	MIMAT0007892	CCCCAGGGCGACGCGGCGGG	90.0	gTGGCTCTGCAGTGCAGGGTCCGAGGTAtgCAGAGCCAcCCCGCCGC	45	F:tcataaCCCCAGGGCGAC; R1	61	56	0.82
miR-3656-5p	MIMAT0018076	GGCGGGUGCGGGGGUGG	88.3	cTGGCTCTGACCTGGTGTCGTGGAGTCGctCAGAGCCAgCCACCCC	25	F:agataaataaGGCGGGTGCG; R2	60	57	0.83
miR-3665-5p	MIMAT0018087	AGCAGGUGCGGGGCGGCG	83.3	gTGGCTCTGCAGTGCAGGGTCCGAGGTATGCAGAGCCAcCGCCGCC	30	F:agagatcaAGCAGGTGCGG; R1	60	56	0.84
miR-3960-5p	MIMAT0019337	GGCGGCGGCGGAGGCGGGGG	95.0	cTGGCTCTGACCTGGTGTCGTGGAGTCGttCAGAGCCAgCCCCCG	40	F:agataaataaGGCGGCGGC; R2	61	60	0.78
miR-4488-5p	MIMAT0019022	AGGGGGCGGGCUCCGGCG	88.9	cgTGGCTCTGCAGTGCAGGGTCCGAGGTAtgCAGAGCCAcCGCCGGAG	30	F: atacttAGGGGGCGGGCT; R1	62	54	n.d
miR-4497	MIMAT0019032	CUCCGGGACGGCUGGGC	82.4	gTGGCTCTGcaGTGCAGGGTCCGAGGTAtgCAGAGCCAcGCCCAGC	25	F: actgacagtCTCCGGGACG; R1	60	56	0.84
miR-4787-5p	MIMAT0019956	GCGGGGGUGGCGGCGGCAUCCC	86.3	cgTGGCTCTGCAGTGCAGGGTCCGAGGTAtgCAGAGCCAcGGGATGCCGC	35	F: aataaGCGGGGGTGGCG; R1	61	57	0.80
Spike-A^b^	Androvic et al. (2019)	UGCAGCCCUACCGACACGUUCC	63.0	cTGGCTCTGACCTGGTGTCGTGGAGTCGtgCAGAGCCAgGGAACG	30	F: gaagatcaaTGCAGCCCTACC; R2	60	61	0.85

Sequence of oligonucleotides is presented in 5′ to 3′ direction. *Ta*, annealing temperature.

Universal reverse primers of qPCR: 5′-CAGTGCAGGGTCCGAGGTA (R1) or 5′-ACCTGGTGTCGTGGAGTCG (R2).

^a^ miRNA, reference candidates were selected based on microarray transcript expression profiling for plasma of healthy human males (this study) or were taken based on their reported appropriateness for normalization in the context of human blood plasma (hsa-miR-93-5p: ((Niu et al., 2016), (Zalewski et al., 2017) and references reviewed by (Donati et al., 2019); hsa-miR-126-3p: (Faraldi et al., 2019)).

^b^ Synthetic spike-in control miRNA with arbitrary sequence.

*E*: amplification efficiency; n.d.: not determined because of only sporadic expression of miR-4488. Underlined sequence: part of stem-loop RT primer for miRNA-specific binding or binding site of the RT primer on the 3′ end of the target miRNA. Small letters in the stem-loop RT primer: spacer required to form the secondary structure of the stem-loop RT primer. Small letters at the 5′ end of the forward qPCR primer: arbitrary tail sequence elevating primer’s melting temperature. Sequences of all stem-loop primers are compatible with probe-based fluorescence monitoring using the hydrolysis probe “T+G+G+C+T+C+T+G” modified by locked nucleic acid (LNA) nucleotides except of the 5’ base. LNA modification is designated by the plus (+) sign preceding the nucleotide. This probe sequence is identical with probe #21 of the Universal ProbeLibrary (Merck; formerly Roche Life Science) phased out by the end of 2020.

For comparison, we tested the plasma normalizers miR-126-3p (Faraldi et al., 2019) and miR-93-5p (Liu et al., 2014; Niu et al., 2016; Zalewski et al., 2017). Both are among the seven potential miRNA reference genes included in Qiagen’s Serum/Plasma Focus miRNA PCR Panels (Qiagen, 2022).

Stem-loop RT-qPCR assays were established for 13 of the 14 miRNAs of interest (except of miR-1469, see below). Positive RNA templates for assay set-up were isolated from the human cancer cell lines HepG2, MDA-MB-231 and TR146 (Supplementary File S1). The latter cell line used for most of the assays (*n* = 9), was subjected to genotype-based authentication at a commercial service provider. The profiling of eight autosomal short tandem repeat (STR) core markers and the gender marker amelogenin confirmed its identity (Supplementary File S1 and Supplementary Table S10). Only one allele differed from the publicly available STR profile.

Specificity of stem-loop RT-qPCR assays was concluded from melt-curve analysis and gel electrophoresis (Supplementary Figure S3 and Supplementary File S1). The level and stability of miRNA plasma abundances was determined on a sample set collected from 32 probands classified into the four stress-coping styles based on the self-report instrument MCI. They represented high-anxious and non-defensive stress copers as well as sensitizers and repressors (*n* = 8 per group; Supplementary Table S2). In general, the miRNAs targeted were consistently detected by stem-loop RT-qPCR with the exception of the sporadically appearing miR-4488 (two positives).

The abundance of miR-1469 was quantified using the S-Poly(T) method (Supplementary Figure S4). It was found to be consistently abundant across the 32 plasma samples, although at highly diverse and mostly rather low levels (∆*Cq*: 12, median *Cq*: 30.1, respectively).

Consequentially, miR-4488 and miR-1469 were excluded from further analysis of abundance stability due to sporadic occurrence or poor stability.

### Stability assessment of the selected miRNA reference candidates

**FIGURE 3:**
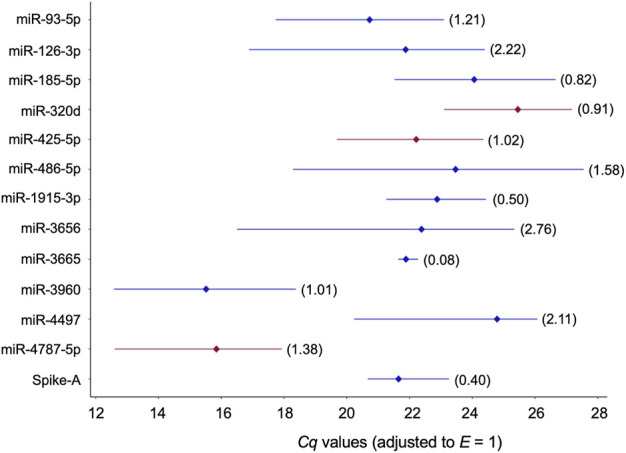
Profiles of *Cq* values of 13 candidate reference miRNAs across 32 blood plasmas derived from probands with different modes of cognitive stress coping. *Cq* values were corrected with the amplification efficiency value determined for the respective qPCR assay (Table 2). The median value is depicted by the diamond. The percent coefficient of variation (*CV*) is given in parentheses. miRNAs composing the two-gene *NF*s either recommended for plasma or the two stress-coping dimensions are highlighted in red.

**FIGURE 4:**
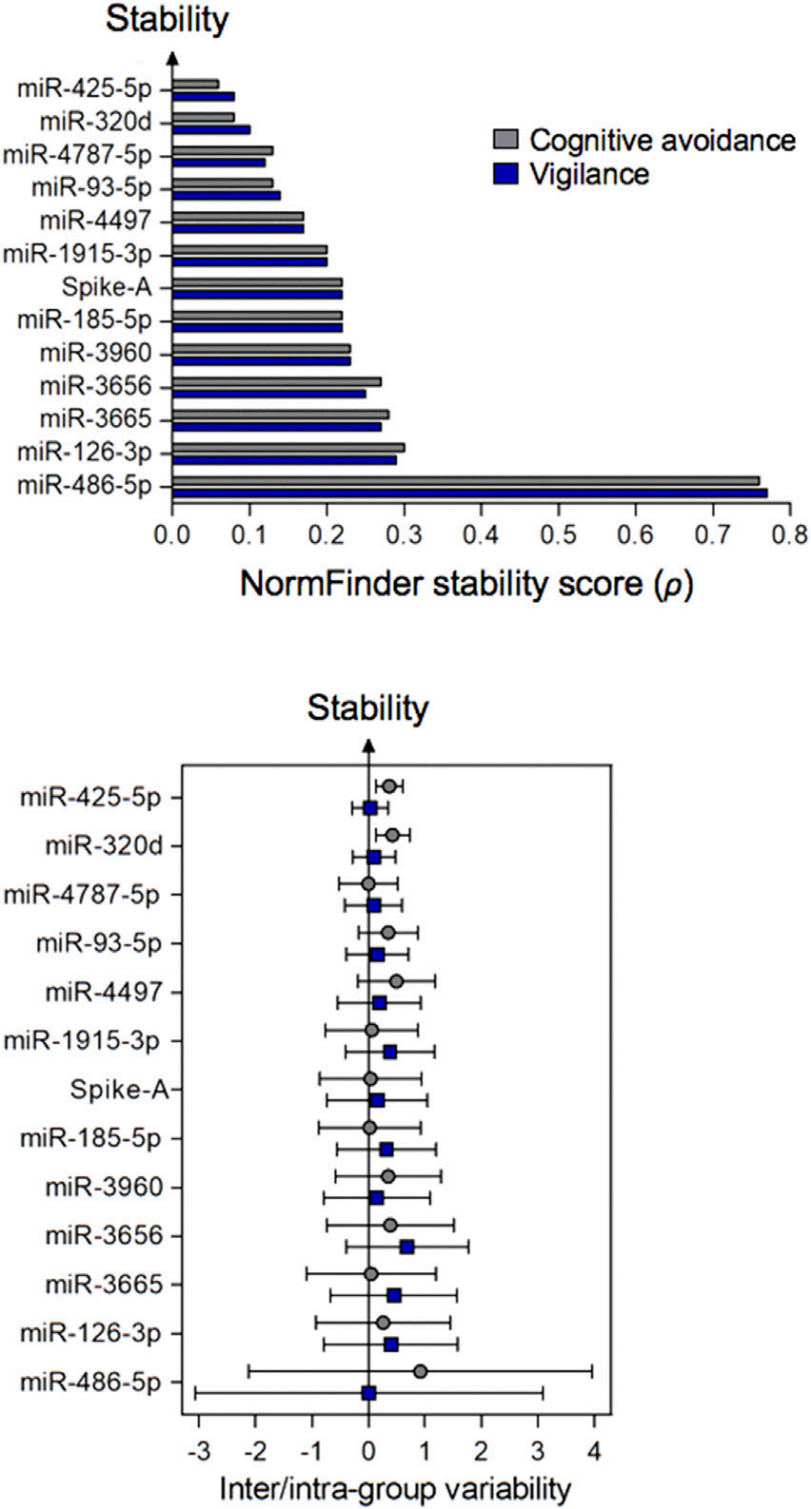
Stability of plasma miRNAs (top figure) and their inter- and intra-group variation (bottom figure) calculated by the NormFinder algorithm. Samples were grouped according to the stress-coping dimensions of cognitive avoidance or vigilance. The circles or squares of the bottom figure relate to inter- or intra-group analysis, respectively. Error bars reflect the extent of variability. Minimal intergroup variation (close to zero) and small error bars are indicative for a stable miRNA. miRNAs are ranked in descending order based on the stability scores of NormFinder.

The remaining twelve miRNA sequences were consistently detected (Table 3; Figure 3 and Supplementary Table S11). Their abundance levels differed considerably ranging from low-to medium in case of miR-486-5p and miR-320d (*Cq* values of 27.5 and 27.2, respectively) up to very high in case of miR-3960 and miR-4787-5p (*Cq*: 12.6). They differed considerably in abundance stability ranging from poor to high or extraordinarily high (*CV* range: 2.76 down to 0.50 and 0.08; Figure 3, Table 3, and Supplementary Table S12) and showed mostly low inter- and intragroup variability assessed with the NormFinder algorithm (Figure 4).

**TABLE 3:** Abundance stability of candidate miRNA references in plasma of healthy male Caucasians.

***CV***		Scores of statistical algorithms for stability assessment						Weighted comprehensive analysis*		
		geNorm		BestKeeper		NormFinder				
		miRNA	*M*	miRNA	*SD*	miRNA	*ρ*	miRNA	score	final rank
miR-3665	0.08	miR-425-5p	0.30	miR-3665	0.09	miR-425-5p	0.31	**miR-425-5p**	0.10	1
Spike-A	0.40	miR-93-5p	0.36	miR-1915-3p	0.33	miR-320d	0.38	**miR-320d**	0.13	2
miR-1915-3p	0.50	miR-320d	0.37	Spike-A	0.56	miR-4787-5p	0.50	miR-93-5p	0.17	3
miR-185-5p	0.82	miR-185-5p	0.65	miR-185-5p	0.80	miR-93-5p	0.54	miR-1915-3p	0.25	4
miR-320d	0.91	Spike-A	0.79	miR-320d	0.94	miR-4497	0.72	miR-185-5p	0.25	5
miR-3960	1.01	miR-1915-3p	0.85	miR-4497	0.96	miR-1915-3p	0.80	Spike-A	0.27	6
miR-425-5p	1.02	miR-3665	0.90	miR-4787-5p	0.99	miR-185-5p	0.88	miR-3665	0.27	7
miR-93-5p	1.21	miR-4787-5p	0.94	miR-425-5p	0.99	Spike-A	0.88	miR-4787-5p	0.31	8
miR-4787-5p	1.38	miR-4497	0.97	miR-93-5p	1.18	miR-3960	0.93	miR-4497	0.35	9
miR-486-5p	1.58	miR-3960	1.02	miR-3960	1.36	miR-3656	1.12	miR-3960	0.43	10
miR-4497	2.11	miR-126-3p	1.08	miR-126-3p	1.40	miR-3665	1.13	miR-126-3p	0.49	11
miR-126-3p	2.22	miR-3656	1.13	miR-3656	1.44	miR-126-3p	1.18	miR-3656	0.50	12
miR-3656	2.76	miR-486-5p	1.44	miR-486-5p	2.99	miR-486-5p	3.03	miR-486-5p	1.00	13

*CV*, coefficient of variation; *SD*, standard deviation; *M* and *ρ*, stability scores of GeNorm and NormFinder, respectively.

*Comprehensive score of ComprFinder; the algorithm uses weighted standardization instead of the geometric mean of the individual scores applied by RefFinder (www.heartcure.com.au/reffinder/?type=reference); score is based on the stability values of geNorm, BestKeeper and NormFinder, but not of the *CV* analysis; higher stability is indicated by a lower comprehensive score.

Mature miRNAs highlighted by blue bold font composed the duo *NF* being most appropriate in the condition of blood plasma donated by healthy Caucasian males.

While these putative normalizers were not differently abundant between phase-2 participants with either low or high vigilance scores (*p* > 0.15), the analogous comparison for the coping dimension of cognitive avoidance yielded only a single case of different abundance (miR-3665: *p* = 0.040 without adjusting for multiple testing). Correcting the significance levels according to the method of (Benjamini and Hochberg, 1995) recommended for multiple comparisons rendered this relationship non-significant. However, it has to be noted that while the *p*-value adjustment decreases the probability of a type-I error, it might favour a type-II error, thus reduces the power of the test. Taken together, we concluded that in general, intergroup variation of the twelve consistently abundant miRNAs would likely not impair quantification of a miRNA of interest in the experimental setting.

Since knotting the ends of mature miRNAs into a hairpin-stem structure can extend their half-life (Belter et al., 2014), we asked whether miRNA’s plasma stability would be related to the extent of secondary-structure formation. However, *MFE* of folding was not related to the *CV* of miRNA’s plasma abundance (Pearson’s correlation coefficient *r* = 0.03, *p* = 0.93, *n* = 12). This clearly implicated that the steady state of miRNA abundance is regulated in a more complex manner going beyond the mere influence of secondary structure.

### Folding of spike-in miRNA controls

A synthetic, unstructured miRNA-like arbitrary sequence was used as spike-in process control to monitor RNA extraction, cDNA synthesis, and qPCR technical quality. Its uniformity of recovery was good based on low *CV* (0.40) and was only outperformed by miR-3665 (Table 3). We are unaware of attempts to further increase the uniformity of spike recovery by using miRNAs with knotty secondary structure that were reported to convey nuclease resistance outside of the RNA-induced silencing complex (Belter et al., 2014). Disclosed sequences of miRNA spike-ins currently in use for mammalian gene expression analysis are either linear (*MFE*: 0.0 kcal/mol) or form hairpins of low to moderate stability that did not involve both ends of the miRNA spike (*MFE*: 0.4 to −3.5 kcal/mol, Figure 2, Supplementary Table S13). Future experimentation should address, whether the recovery of spike-in controls can be improved by using worm (*Caenorhabditis elegans*), plant (*Arabidopsis thaliana* or *Oryza sativa*), artificial or other miRNAs that form hairpins of elevated stability similar to those predicted for our extremely GC-rich miRNAs (*MFE* ≤ −4.1 kcal/mol, Figure 2).

### Stability of single miRNAs and their duo and trio combinations

The stability of single miRNAs across the plasma samples of healthy male Caucasians was ranked using the mainstream algorithms geNorm, NormFinder, and BestKeeper. In line with various other reports, differences were observed between the three resulting lists (Table 3). To compile a final ranking, we applied ComprFinder, a novel tool that maintains the dimension of an individual stability gain or loss by standardising the range of the stability score across the individual statistical algorithms. The top-eight ranks were occupied by a mixture of miRNAs with moderate or extreme GC content (moderate: miR-425-5p, miR-320d, miR-93-5p, and miR-185-5p; extreme: miR-1915-3p, miR-3665, miR-4787-5p, and miR-4497, respectively; Table 3). Ranking miR-93-5p at position three, confirmed a former report that recommended this miRNA as normalizer for plasma or serum samples of healthy individuals (Supplementary Table S14).

The intrinsic variability that a single reference shows across individual samples and/or experimental conditions, can be minimised by using multiple internal controls (Chervoneva et al., 2010). Often, studies based on high-throughput transcriptome data found a combination of just two normalizers to be sufficient for quantitative accuracy. This minimises the number of assays to be processed for each sample, template input and costs, hence increases practicality. The minimal/optimal number of reference genes for adequate normalization of qPCR data is commonly determined using the geNorm’s pairwise variation coefficient *V*_*n*_/*V*_*n+1*_ calculated between the sequential normalization factors *NF*_*n*_ and *NF*_*n+1*_. A *V*_*n*_/*V*_*n+1*_ value of less than 0.15 indicates that *n* reference genes/sequences would be the best compromise between accuracy and expenditure compared with *n  +  *1 genes/sequences. For the condition of human plasma, just two miRNAs were sufficient for accurate normalization and adding another normalizer sequence would not significantly improve reliability (Supplementary Figure S5). Meeting the cut-off of *V*_*2/3*_ < 0.15 as in our case of undirected high-throughput selection, indicates that high-quality references were selected for the particular experimental condition. Therefore, all possible sequence duos, but also trio combinations were assessed for stability assessment. Screening was performed using NormiRazor, a web tool that integrates the established normalization algorithms geNorm, NormFinder and BestKeeper. It best-ranked the combination of miR-320d and miR-4787-5p for all 32 plasma samples, *i.e.* without consideration of coping-style stratification (Table 4, Supplementary Table S15). For either of the stress-coping dimensions of cognitive avoidance or vigilance, the duo *NF* composed of miR-425p and miR-4787-5p was identified as best performing (Table 4).

**TABLE 4:** Stability scores of miRNA reference gene pairs in plasma of male Caucasians differing in their mode of cognitive stress coping.

Rank^a^	Coping styles				Across coping styles	
	Cognitive avoidance	Score	Vigilance	Score	All plasmas	Score
1	miR-425-5p, miR-4787-5p	0.035	miR-425-5p, miR-4787-5p	0.035	miR-320d, miR-4787-5p	0.03
2	miR-320d, miR-4787-5p	0.038	miR-320d, miR-4787-5p	0.038	miR-93-5p, miR-4497	0.06
3	miR-93-5p, miR-4787-5p	0.05	miR-93-5p, miR-4787-5p	0.05	miR-93-5p, miR-1915-3p	0.07
4	miR-93-5p, miR-1915-3p	0.07	miR-93-5p, miR-1915-3p	0.07	miR-425-5p, miR-4497	0.08
5	miR-93-5p, miR-4497	0.09	miR-93-5p, miR-4497	0.08	miR-425-5p, miR-4787-5p	0.09
6	miR-425-5p, miR-4497	0.11	miR-425-5p, miR-4497	0.10	miR-425-5p, miR-320d	0.13
7	miR-425-5p, miR-320d	0.12	miR-320d, miR-4497	0.13	miR-320d, miR-4497	0.13
8	miR-320d, miR-4497	0.13	miR-425-5p, miR-320d	0.14	miR-425-5p, miR-1915-3p	0.15
9	miR-425-5p, miR-1915-3p	0.14	miR-425-5p, miR-1915-3p	0.14	miR-425-5p, miR-93-5p	0.15
10	miR-320d, miR-93-5p	0.18	miR-4787-5p, miR-3960	0.19	miR-320d, miR-93-5p	0.16

^a^ Average rank of GeNorm, NormFinder and BestKeeper determined by NormiRazor.

For rank lists of single miRNA, references and the complete information on duo and trio *NF*s, see Supplementary Information.

Central to choosing multiple reference genes for normalization of RT-qPCR expression data is to avoid the possibility of co-regulation. To minimise the chance for this bias, we assured that the NF-composing miRNAs were neither members of the same miRNA family (miR family members are often co-expressed, either from the same pri-miR transcript or from distinct but co-regulated loci) nor were they clustered on the genome (genomic distance: >10 kb). A Pearson’s correlation coefficient of *r* ≥ 0.9 was considered as threshold to highlight the rather poor effectiveness of a combination of two miRNA normalizers (Babion et al., 2017). In our case, this critical level of correlation was not reached, neither for miR-320d & miR-4787-5p that were found to be most appropriate for all 32 plasma samples (*r* = 0.69, *p* = 0.000017), nor for the combination of miR-425-5p & miR-4787-5p being most stable under the conditions of cognitive avoidant or vigilant stress-coping (*r* = 0.66 to 0.82, *p* = 0.02–0.11).

Associations between the mRNA targets and pathways of the paired miRNAs were either absent (miR-320d and miR-4787-5p) or minor (miR-425-5p and miR-4787-5p) according to the miRNA Pathway Dictionary Database (Supplementary File S1). Mostly, they targeted different signalling or metabolic pathways (Supplementary Figure S6, Supplementary Tables S16, S17).

### Impaired ability of sRNA-seq to detect the novel extremely GC-rich miRNA normalizers

Here we return to the bias of sRNA-seq against detection of miRNAs with high and extreme GC contents (see above) providing a focused analysis for the novel extremely GC-rich miRNA normalizers (Supplementary Table S18). While our sRNA-seq protocol successfully detected the sequences of moderate GC-content such as miR-93-5p, miR-126-3p, miR-185-5p, miR-320d, miR-425-5p, and miR-486-5p (GC content: 45%–65%), it caused false-negative detection of the extremely GC-rich miRNAs miR-1915-3p, miR-3656-5p, miR-3665-5p, miR-3960-5p, miR-4488-5p, miR-4497 and miR-4787-5p (GC content: 82%–95%; Table 2). In contrast to the miRNAs with moderate GC contents (<64%), they were significantly more prone to intramolecular hybridization as indicated by more negative Δ*G* values (medians of −0.7 and −4.5 kcal/mol, respectively, Supplementary Table S9; *p* = 0.012). This confirmed the expectation that sequences of elevated GC content are likely to have more stable secondary structure (Chan et al., 2009).

## Discussion

**FIGURE 5:**
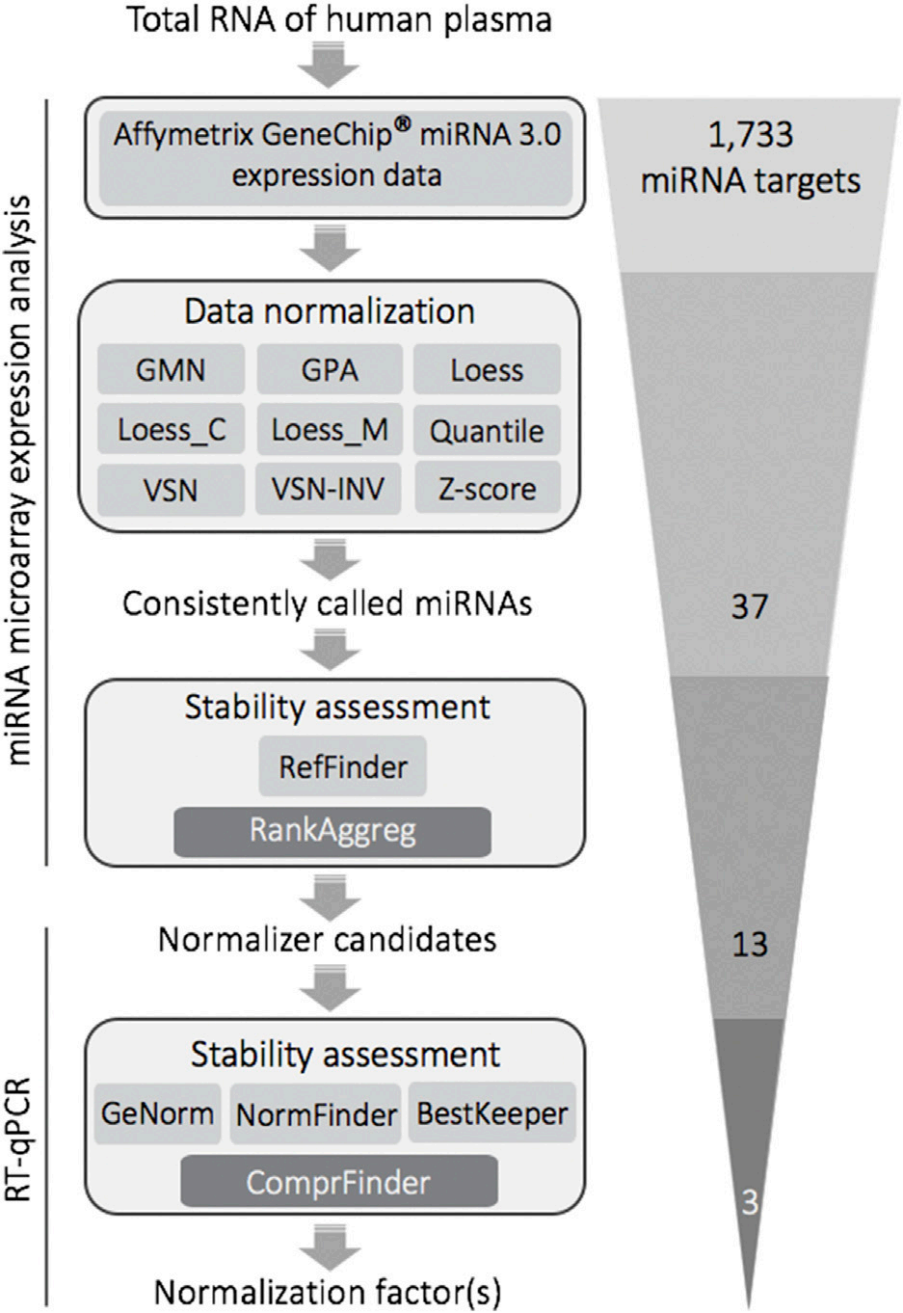
Selection and validation of stable reference miRNAs for RT-qPCR-based quantitative expression analysis of plasma donated by human males differing in cognitive stress coping styles: methodological flow chart and filtering steps. The final trio of stable miRNAs was composed of miR-320d, miR-4787-5p (duo *NF* for plasma of Caucasian males) and miR-425-5p (composing the duo *NF* together with miR-4787-5p when plasma samples were stratified into cognitive avoidant or vigilant copers). Abbreviations: GMN, Global Mean Normalization; GPA, Generalized Procrustes Analysis; Loess, locally estimated scatterplot smoothing; Loess_C, Cyclic Loess; Loess_M, Modified Loess; VSN, Variance Stabilization Normalization; VSN-INV, VSN based on invariant miRNAs; Z-score, Z-Score Normalization; RefFinder, comprehensive analysis of stability based on the common statistical algorithms geNorm, NormFinder, BestKeeper, and Δ*Ct* method; RankAggreg, software package for weighted rank aggregation; ComprFinder, comprehensive stability ranking algorithm based on weighted standardisation.

The complexity of biological systems contradicts the hypothetical concept of a single-best normalization strategy fitting all physiological contexts. For the condition of plasma sampled from healthy Caucasian men and additionally stratifying for modes of cognitive stress-coping, this study combined expression analysis on a miRNA microarray and stem-loop RT-qPCR to select and verify stable miRNAs, respectively (Figure 5). Expression analysis on a miRNA microarray is still a contemporary technique (Pimentel et al., 2015) that is superior to sRNA-seq in targeting the highly GC-rich fraction of the miRNome (Figure 1 and see below) and well balances precision, accuracy, sample input, expediency and costs.

Our strategy resulted in an extended miRNA repertoire for context-optimised RT-qPCR normalization (*n* = 8). The set of normalizers included miR-3960-5p, the tumour suppressors miR-185-5p, miR-3665 and miR-4787-5p, an oncogenic miRNA, miR-425-5p, and the Janus-faced tumour molecules miR-93-5p, miR-320d and miR-1915-3p that fulfil either tumour-suppressive oroncogenic functions depending on the cellular context and the downstream targets they affect (Han et al., 2020). Their key characteristics are summarised in Supplementary Table S17. All circulating miRNA candidates of this study might (also) appear as extracellular exosomal miRNAs (exomiRs) in human plasma/serum (Supplementary Table S9). The lipid-bilayer enclosing of exosomes might add to their stability in the cell-free circulation by protecting against nucleolytic degradation. Moreover, ordered secondary structures can provide nuclease resistance for miRNAs residing outside of the RNA-induced silencing complex (Gangemi et al., 2020), thus, can further add to their superior stability. At least *in silico*, the extremely GC-rich mature miRNAs were more structured as indicated by more negative Δ*G* values (*p* = 0.013, Supplementary Table S9), thus confirming the assumption that a sequence of higher GC content is likely to have more stable folding.

The high quality of the novel miRNA normalizers is indicated by the following. First, by the suggestion of geNorm to use just two sequences for adequate normalization. Second, stability evaluation using *CV* analysis identified two miRNAs that showed extraordinary or high uniformity of abundance across the plasma samples of the 32 human males, namely, miR-3665 and miR-1915-3p (*CVs*: 0.08 and 0.50, respectively). Third, we confirmed the status of stable abundance for circulating miR-320d and miR-425-5p. While miR-320d was formerly identified as a suitable reference gene for the plasma of healthy human males (Faraldi et al., 2019), miR-425-5p was reported to be stable in plasma exosomes of healthy donors likely of Chinese descent (Han et al., 2020). However, an ethnicity issue might limit the use of the latter miRNA. miR-425-5p that is among the seven potential miRNA reference genes of Qiagen’s Serum/Plasma Focus miRNA PCR Panels (Qiagen, 2022), is ∼8-fold more abundant in the blood of black compared to white individuals (Patel et al., 2019). It is currently unknown how this ethnicity difference translates to plasma or serum samples of other ethnic groups.

The disclosure of assay oligonucleotides (Table 2) allows to estimate the putative impact of heterogeneity resulting from isomiRs— 5′, 3′ or internal modifications of a canonical miRNA regulated post-transcriptionally (Tomasello et al., 2021). For example, the recommended plasma/serum normalizer hsa-miR-425-5p ((Qiagen, 2019); Supplementary Table S14 and this study) is subject of dynamic regulation of 3′ isomiRs resulting from nucleotide addition, deletion or substitution of a canonical miRNA (Dhanoa et al., 2019). Since 3′-addition or -trimming variants are relatively widespread (Nejad et al., 2018), the canonical length of a miRNA, currently defined in the miRBase based on a landmark study of miRNA cloning, has to be taken with caution.

This study and earlier reports encountered the difficulty or even failure to detect our GC-extreme miRNA normalizer candidates in human plasma by sRNA-seq (Supplementary Table S19, Figure 1). This is in line with the common underrepresentation of GC-extreme sequences in NGS libraries (Browne et al., 2020) and especially applies to miRNome sequencing of biofluids such as plasma where the poor sensitivity of NGS meets low template input (Raabe et al., 2014; Backes et al., 2016; Coenen-Stass et al., 2018; Shi et al., 2021). Hence, these types of sample material require a minimum number of PCR cycles (*e.g.,* 4–8) to produce sufficient library yield. In addition to GC bias of PCR amplification (Wright et al., 2019) implemented into common sRNA-seq protocols (Burgos et al., 2013; Backes et al., 2016; Yagi et al., 2017), coverage bias can be caused by various steps of the library preparation such as adapter ligation (Barberan-Soler et al., 2018), RT (Wright et al., 2019), and other sample-handling procedures (Browne et al., 2020). For example, bias of PCR amplification can be mitigated or even resolved by various means (Shi et al., 2021) including automation of library preparation (Santacruz et al., 2022), fine-tuning of cycling conditions, the use of PCR additives and/or outperforming polymerases (and master mixes) that reduce the impact of secondary structure (Wright et al., 2019).

In summary, a detailed understanding of the source and nature of the under-coverage of extremely GC-rich miRNAs by sRNA-seq is essential to interpret the sequencing outcome and to avoid or at least reduce the bias, especially for low input materials (reviewed by (Shi et al., 2021)). Alternatively, the complementary use of methods might help to reveal the full spectrum of miRNA abundances.

## Conclusion

Each expression study set-up requires adequate selection of multiple, preferably same-class normalizers to reduce technical noise, hence to minimise masking or exaggerating biologically meaningful changes. Here, we expanded the panel of putative miRNA normalizers for the context of human (and possibly also animal) plasma by adding miR-3665, miR-1915-3p, miR-185-5p, miR-320d, miR- 3960-5p, miR-425-5p, miR-93-5p, and miR-4787-5p. For the GC-moderate miR-320d of this panel, we confirmed its status of a plasma normalizer reported before. In addition, we identified two extraordinarily stable miRNAs, miR-3665, miR-1915-3p, that were extremely GC-rich (>80%, Supplementary Table S9). Another extremely GC-rich miRNA normalizer, miR-4787-5p, was part of the most appropriate two-gene *NF* recommended for healthy male Caucasians classified as either cognitive-avoidant or vigilant stress copers according to the self-report instrument MCI. The novel normalizers can serve as a tool for identifying miRNA plasma biomarkers related to cognitive stress-coping and other (patho)physiological human (or animal) conditions.

Last but not least, we enhanced assay reliability by providing information on putative cross-hybridization of the designed stem-loop RT-qPCR oligonucleotides and by disclosing their sequences as requested by the original minimum information for the publication of quantitative PCR experiments (MIQE) guidelines (Bustin et al., 2009). This would also facilitate cost reduction compared to a commercial solution.

## Data Availability

The datasets presented in this study can be found in online repositories. The names of the repository/repositories and accession number(s) can be found below: https://www.ncbi.nlm.nih.gov/geo/, GSE59359; https://www.ncbi.nlm.nih.gov/geo/, GSE209568.

## Abbreviations

Cq, cycle of quantification; CV, coefficient of variation; E, PCR efficiency; EVs, extracellular vesicles; GC content, guanine-cytosine content; LNA, locked nucleic acid; MCI, Mainz Coping Inventory; MFE, minimal free energy; miRNA, micro RNA; NF, normalization factor; NGS, next-generation sequencing; RT-qPCR, reverse-transcription quantitative PCR; sRNA-seq, small RNA sequencing; STR, short tandem repeat; UMI, unique molecular identifier.
