# Corrigendum: Identification of extremely GC-rich micro RNAs for RT-qPCR data normalization in human plasma

**DOI:** 10.3389/fgene.2023.1216150

**Published:** 2023-05-31

**Authors:** Volker Baumann, Angelos-Theodoros Athanasiou, Omid R. Faridani, Andreas R. Schwerdtfeger, Bernard Wallner, Ralf Steinborn

**Affiliations:** ^1^ Genomics Core Facility, VetCore, University of Veterinary Medicine, Vienna, Austria; ^2^ Garvan Institute of Medical Research, Sydney, NSW, Australia; ^3^ Lowy Cancer Research Centre, School of Biomedical Sciences, University of New South Wales, Sydney, NSW, Australia; ^4^ Institute of Psychology, Karl-Franzens-University Graz, Graz, Austria; ^5^ Department of Behavioral and Cognitive Biology, University of Vienna, Vienna, Austria; ^6^ Department of Microbiology, Immunobiology and Genetics, University of Vienna, Vienna, Austria

**Keywords:** miRNA expression microarray, small-RNA sequencing, stem-loop reverse-transcription quantitative PCR, human plasma miRNAs, miRNA reference genes, cognitive stress-coping, qPCR normalization

In the original article, the coefficient of variation (*CV*, ratio between standard deviation and mean) was calculated from *Cq* values that results in a profound difference compared to the use of real numbers (Kralik and Ricchi, 2017). We now corrected the calculation by **converting the *Cq* values into relative quantities** (Hellemans et al., 2007), also termed linear-scale transformation or just **linearisation of *Cq* values** (2^−*Cq*^) (Marabita et al., 2016; Sundaram et al., 2019). This was achieved by converting the efficiency-adjusted *Cq* values into n-fold quantities relatively to the lowest expressing sample according to the term $2^{maximum\;Cq\;–\;sample\;Cq}$ .

Therefore, we had to correct the ***CV* values** contained in Figure 3, Table 3 and the **Supplementary Tables S12, S17** as well as the Extended Methods contained in **Supplementary Material S1**.

**FIGURE 3:**
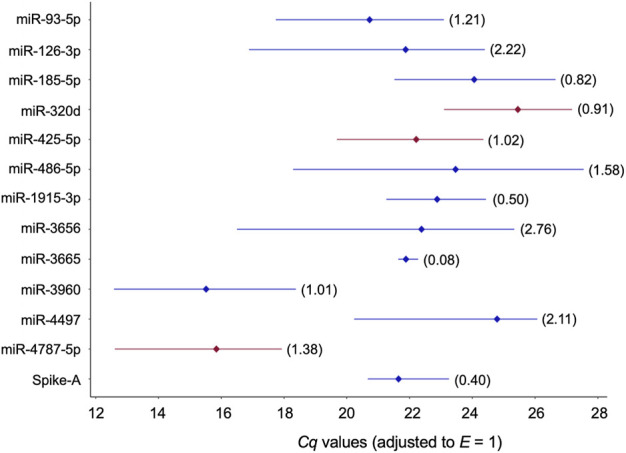
Profiles of *Cq* values of 13 candidate reference miRNAs across 32 blood plasmas derived from probands with different modes of cognitive stress coping. *Cq* values were corrected with the amplification efficiency value determined for the respective qPCR assay (**Table 2**). The median value is depicted by the diamond. The percent coefficient of variation (*CV*) is given in parentheses. miRNAs composing the two-gene *NF*s either recommended for plasma or the two stress-coping dimensions are highlighted in red.

**TABLE 3:** Abundance stability of candidate miRNA references in plasma of healthy male Caucasians.

***CV***		**Scores of statistical algorithms for stability assessment**						Weighted comprehensive analysis*		
		**geNorm**		**BestKeeper**		**NormFinder**				
		**miRNA**	***M***	**miRNA**	***SD***	**miRNA**	***ρ***	**miRNA**	**score**	**final rank**
miR-3665	0.08	miR-425-5p	0.30	miR-3665	0.09	miR-425-5p	0.31	**miR-425-5p**	0.10	1
Spike-A	0.40	miR-93-5p	0.36	miR-1915-3p	0.33	miR-320d	0.38	**miR-320d**	0.13	2
miR-1915-3p	0.50	miR-320d	0.37	Spike-A	0.56	miR-4787-5p	0.50	miR-93-5p	0.17	3
miR-185-5p	0.82	miR-185-5p	0.65	miR-185-5p	0.80	miR-93-5p	0.54	miR-1915-3p	0.25	4
miR-320d	0.91	Spike-A	0.79	miR-320d	0.94	miR-4497	0.72	miR-185-5p	0.25	5
miR-3960	1.01	miR-1915-3p	0.85	miR-4497	0.96	miR-1915-3p	0.80	Spike-A	0.27	6
miR-425-5p	1.02	miR-3665	0.90	miR-4787-5p	0.99	miR-185-5p	0.88	miR-3665	0.27	7
miR-93-5p	1.21	miR-4787-5p	0.94	miR-425-5p	0.99	Spike-A	0.88	miR-4787-5p	0.31	8
miR-4787-5p	1.38	miR-4497	0.97	miR-93-5p	1.18	miR-3960	0.93	miR-4497	0.35	9
miR-486-5p	1.58	miR-3960	1.02	miR-3960	1.36	miR-3656	1.12	miR-3960	0.43	10
miR-4497	2.11	miR-126-3p	1.08	miR-126-3p	1.40	miR-3665	1.13	miR-126-3p	0.49	11
miR-126-3p	2.22	miR-3656	1.13	miR-3656	1.44	miR-126-3p	1.18	miR-3656	0.50	12
miR-3656	2.76	miR-486-5p	1.44	miR-486-5p	2.99	miR-486-5p	3.03	miR-486-5p	1.00	13

*CV*, coefficient of variation; *SD*, standard deviation; *M* and *ρ*, stability scores of GeNorm and NormFinder, respectively.

*Comprehensive score of ComprFinder; the algorithm uses weighted standardization instead of the geometric mean of the individual scores applied by RefFinder (www.heartcure.com.au/reffinder/?type=reference); score is based on the stability values of geNorm, BestKeeper and NormFinder, but not of the *CV* analysis; higher stability is indicated by a lower comprehensive score.

Mature miRNAs highlighted by bold font composed the duo *NF* being most appropriate in the condition of blood plasma donated by healthy Caucasian males.

Based on the adjusted *CV* values, a correction has been made in the **Abstract** (single paragraph). The incorrect statement was “In general, inter-individual variance of miRNA abundance was low or very low as indicated by coefficient of variation (*CV*) values of 0.6%–8.2%. miR-3665 and miR-1915-3p outperformed in this analysis (*CV*s: 0.6% and 2.4%, respectively).” It was corrected to:

“The lowest inter-individual variance of miRNA abundance was determined for miR-3665 and miR-1915-3p [coefficient of variation (*CV*) values: 0.08 and 0.50, respectively].”

A correction has been made to the **Results** section, sub-section *Stability assessment of the selected miRNA reference candidates* (paragraph 1). The sentence previously stated “They showed high to extraordinary uniformity of abundance as indicated by *CV* values of 13.9%–0.6% (Table 3; **Supplementary Table S12**) and mostly low inter- and intragroup variability assessed with the NormFinder algorithm (**Figure 4**).” The corrected text on the *CV* range appears below:

“They differed considerably in abundance stability ranging from poor to high or extraordinarily high (*CV* range: 2.76 down to 0.50 and 0.08; Figure 3, Table 3, and **Supplementary Table S12**) and showed mostly low inter- and intragroup variability assessed with the NormFinder algorithm (**Figure 4**).”

Another correction has been made in this sub-section (paragraph 3). The previous sentence “However, *MFE* of folding was not related to the *CV* of miRNA’s plasma abundance (Pearson’s correlation coefficient *r* = 0.17, *p* = 0.60, *n* = 10).” was adjusted as follows:

“However, *MFE* of folding was not related to the *CV* of miRNA’s plasma abundance (Pearson’s correlation coefficient *r* = 0.03, *p* = 0.93, *n* = 12).”

A correction was made in the **Results** section, sub-section *Folding of spike-in miRNA controls* (paragraph 1). Previously we stated: “Its uniformity of recovery was concluded to be good based on a low *CV* value of 3.1% and was only outperformed by miR-3665 and miR-1915-3p (Table 3).” The corrected sentence appears below:

“Its uniformity of recovery was good based on low *CV* (0.40) and was only outperformed by miR-3665 (Table 3).”

Based on the corrected *CV* analysis, an update on the recommended set of miRNA reference genes was made in the **Discussion** section (paragraph 2). The previous text was: “Our strategy resulted in an extended miRNA repertoire for context-optimised RT-qPCR normalization (*n* = 6). The normalizers include tumour suppressors (miR-3665, miR-4497 and miR-4787-5p), oncogenic (miR-425-5p), and Janus-faced tumour molecules (miR-320d and miR-1915-3p) that fulfil either tumour-suppressive or oncogenic functions depending on the cellular context and the downstream targets they affect (Han et al., 2020).” Here is the adjusted sentence:

“Our strategy resulted in an extended miRNA repertoire for context-optimised RT-qPCR normalization (*n* = 8). The set of normalizers included miR-3960-5p, the tumour suppressors miR-185-5p, miR-3665 and miR-4787-5p, an oncogenic miRNA, miR-425-5p, and the Janus-faced tumour molecules miR-93-5p, miR-320d and miR-1915-3p that fulfil either tumour-suppressive or oncogenic functions depending on the cellular context and the downstream targets they affect (Han et al., 2020).”

A correction has been made to the **Discussion** section (paragraph 3). The previous sentence was: “Second, stability evaluation using *CV* analysis identified two miRNAs, that showed extraordinary even abundances across the plasma samples of the 32 human males, namely, miR-3665 and miR-1915-3p (*CV*s: 0.6% and 2.4%, respectively).” The corrected statement appears below:

“Second, stability evaluation using *CV* analysis identified two miRNAs that showed extraordinary or high uniformity of abundance across the plasma samples of the 32 human males, namely, miR-3665 and miR-1915-3p (*CV*s: 0.08 and 0.50, respectively).”

Finally, a correction has been made to the **Conclusion** section (paragraph 1). The previous information on the set of recommended miRNA normalizers was; “Here, we expanded the panel of putative miRNA normalizers for the context of human (and possibly also animal) plasma by adding miR-3665, miR-1915-3p, miR-320d, miR-4497, miR-425-5p, and miR-4787-5p.” The corrected statement appears below:

“Here, we expanded the panel of putative miRNA normalizers for the context of human (and possibly also animal) plasma by adding miR-3665, miR-1915-3p, miR-185-5p, miR-320d, miR-3960-5p, miR-425-5p, miR-93-5p, and miR-4787-5p.”

The authors apologize for these errors and state that this does not change the scientific conclusions of the article in any way. The original article has been updated.

## References

[B1] Hellemans J. Mortier G. De Paepe A. Speleman F. Vandesompele J. (2007). qBase relative quantification framework and software for management and automated analysis of real-time quantitative PCR data. Genome Biol. 8, R19. 10.1186/gb-2007-8-2-r19 17291332 PMC1852402

[B2] Kralik P. Ricchi M. (2017). A basic Guide to real time PCR in microbial diagnostics: Definitions, Parameters, and everything. Front. Microbiol. 8, 108. 10.3389/fmicb.2017.00108 28210243 PMC5288344

[B3] Marabita F. de Candia P. Torri A. Tegner J. Abrignani S. Rossi R. L. (2016). Normalization of circulating microRNA expression data obtained by quantitative real-time RT-PCR. Brief Bioinform. 17, 204–212. 10.1093/bib/bbv056 26238539 PMC4793896

[B4] Sundaram V. K. Sampathkumar N. K. Massaad C. Grenier J. (2019). Optimal use of statistical methods to validate reference gene stability in longitudinal studies. PLoS One 14, e0219440. 10.1371/journal.pone.0219440 31335863 PMC6650036

