# Supplementary material for: Identification of extremely GC-rich micro RNAs for RT-qPCR data normalization in human plasma
Source: Front Genet. 2023 Jan 4;13:1058668. doi: 10.3389/fgene.2022.1058668 (PMC9846067; doi:10.3389/fgene.2022.1058668)
Supplement: Supplementary file 1 [file DataSheet1.zip › Supporting information/Figure_S1_Removal_of_microarray_background.docx]

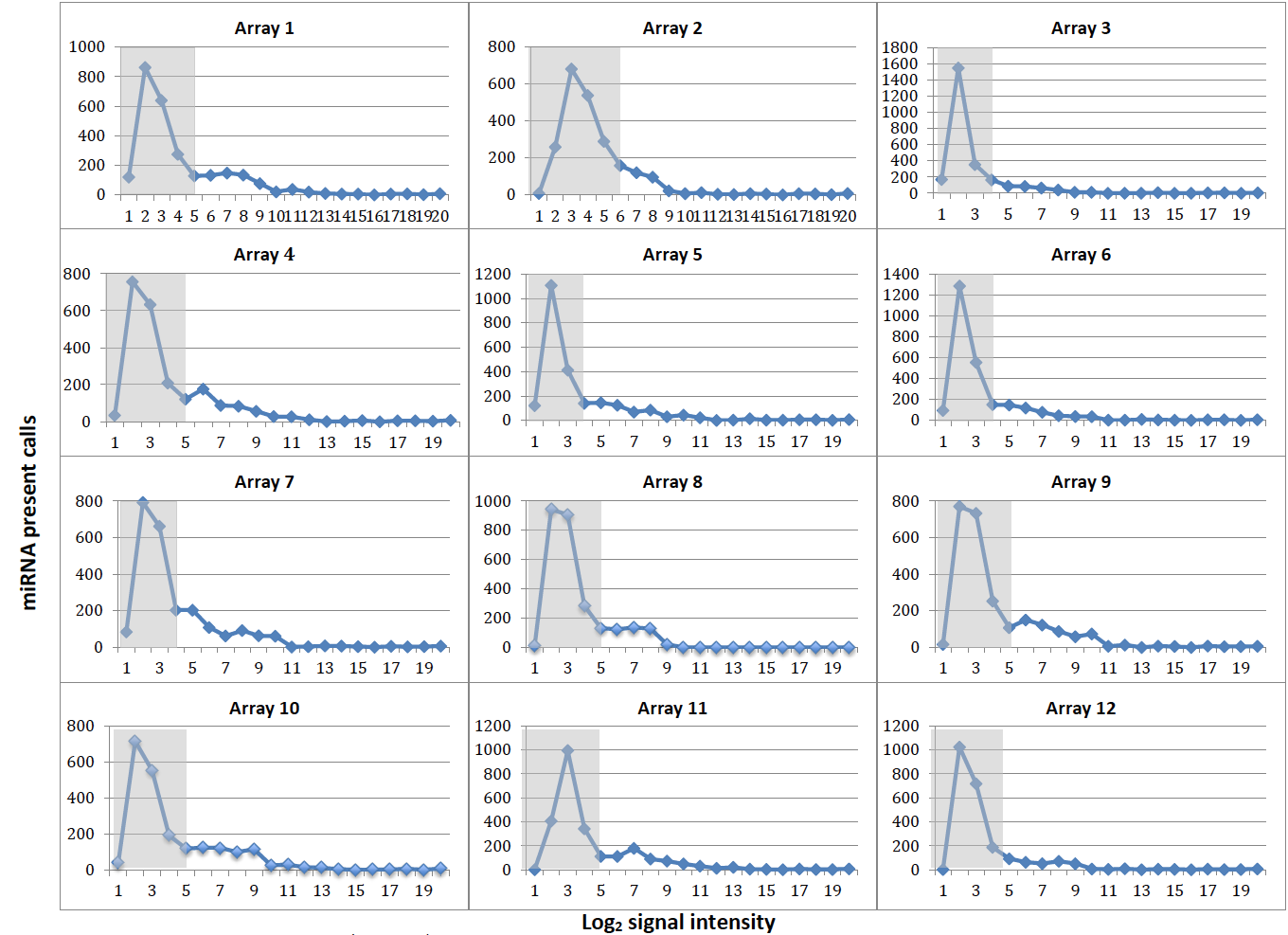


**Figure S1 |**Removal of low intensity signals (grey boxes) not following the expected unimodal signal distribution in a miRNA number per intensity plot. Expression values were grouped by their log_2_ signal intensities.
