# Supplementary material for: Identification of extremely GC-rich micro RNAs for RT-qPCR data normalization in human plasma
Source: Front Genet. 2023 Jan 4;13:1058668. doi: 10.3389/fgene.2022.1058668 (PMC9846067; doi:10.3389/fgene.2022.1058668)
Supplement: Supplementary file 1 [file DataSheet1.zip › Supporting information/Figure_S2_Distribution of log2 signal intensities of normalization approaches.docx]

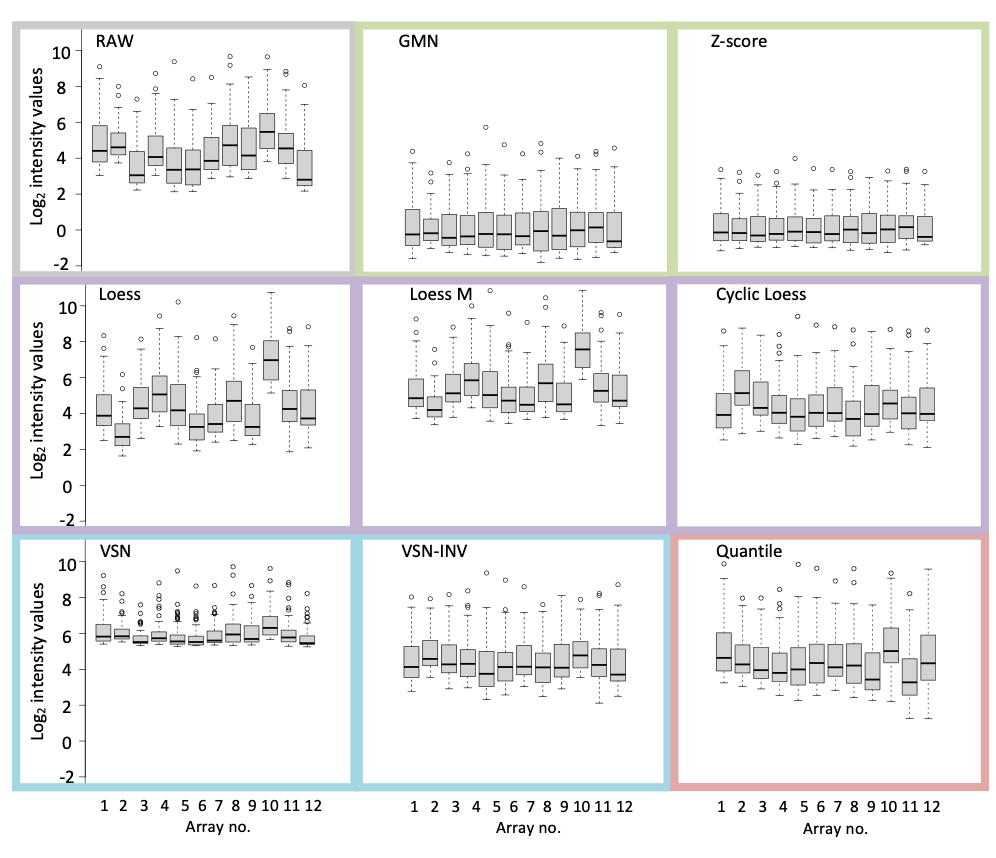


**Figure S2 |** Distribution of log_2_ signal intensities across the eight array normalization approaches. Related calculations procedures are indicated by the same colour around individual plots. Note that Z-score and GMN normalizations intrinsically produce low intensity values (semi-quantitative values with a normalized mean of zero).
