# Supplementary material for: Identification of extremely GC-rich micro RNAs for RT-qPCR data normalization in human plasma
Source: Front Genet. 2023 Jan 4;13:1058668. doi: 10.3389/fgene.2022.1058668 (PMC9846067; doi:10.3389/fgene.2022.1058668)
Supplement: Supplementary file 1 [file DataSheet1.zip › Supporting information/Figure_S3_Melting curves and agarose gels of miRNA assays.docx]

**hsa-miR-93-5p** (MIMAT0000093)

TR146 RT+

TR146 RT-

Subject

NTC

Tm: 83.3°C


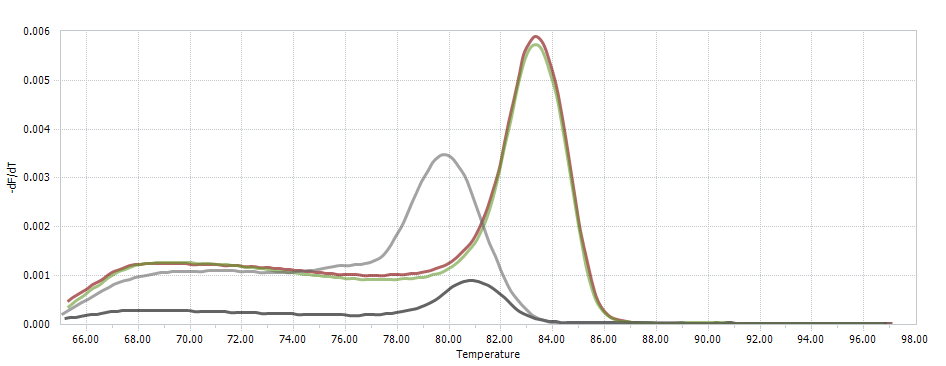

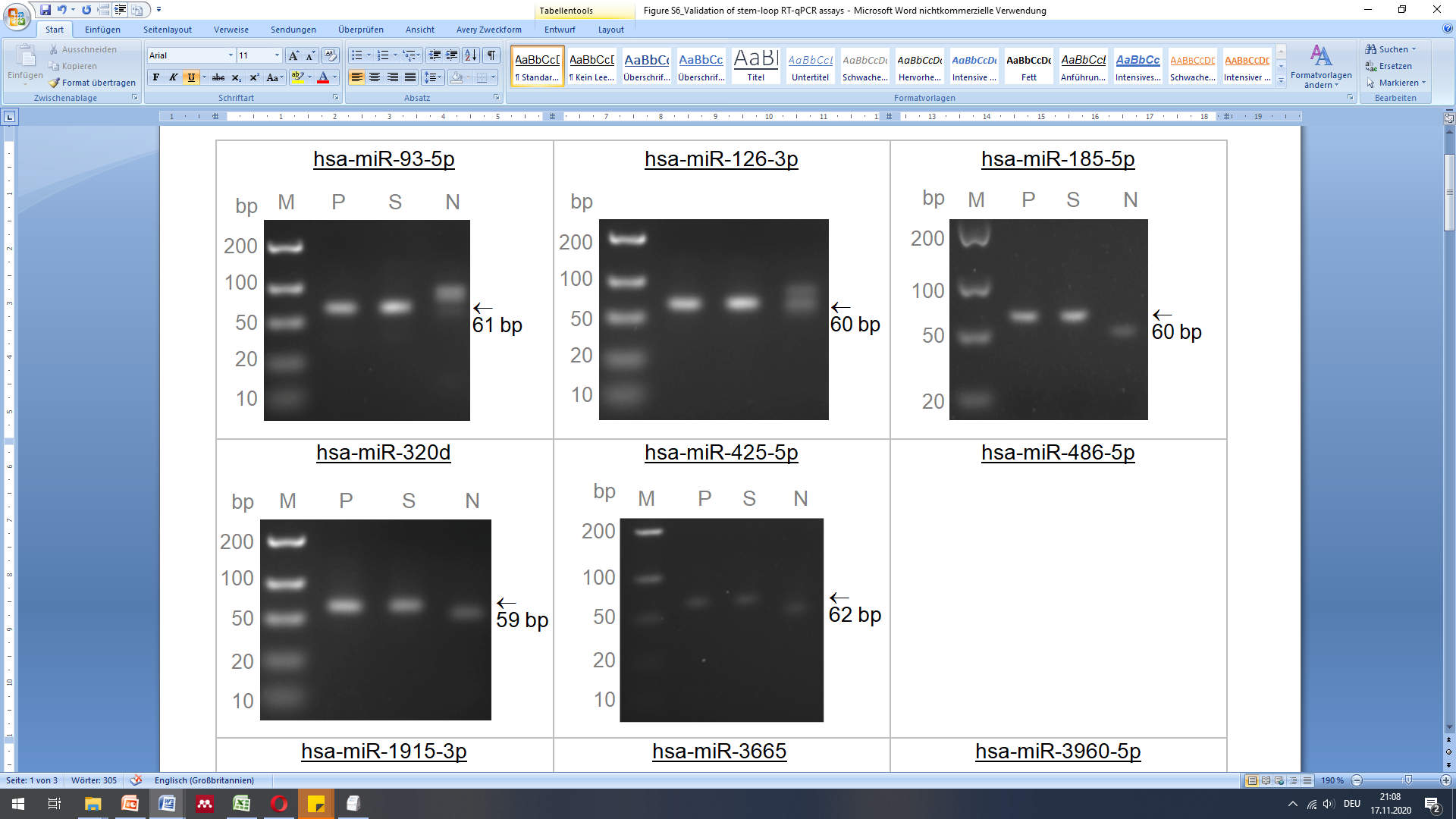


**hsa-miR-126-3p** (MIMAT0000445)

Tm: 82.0°C


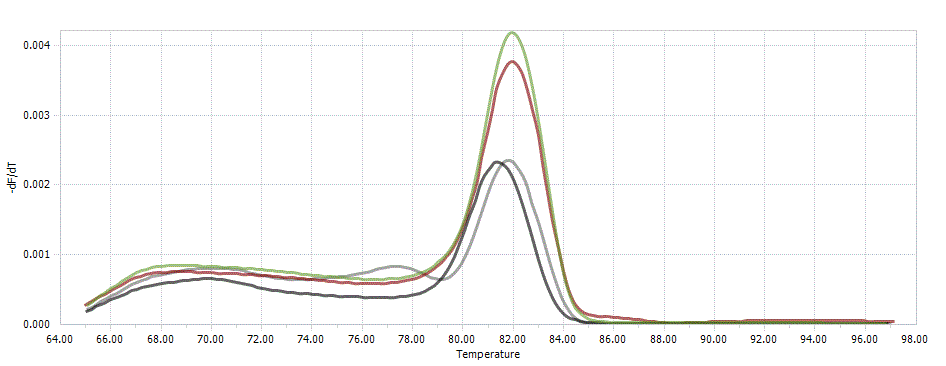

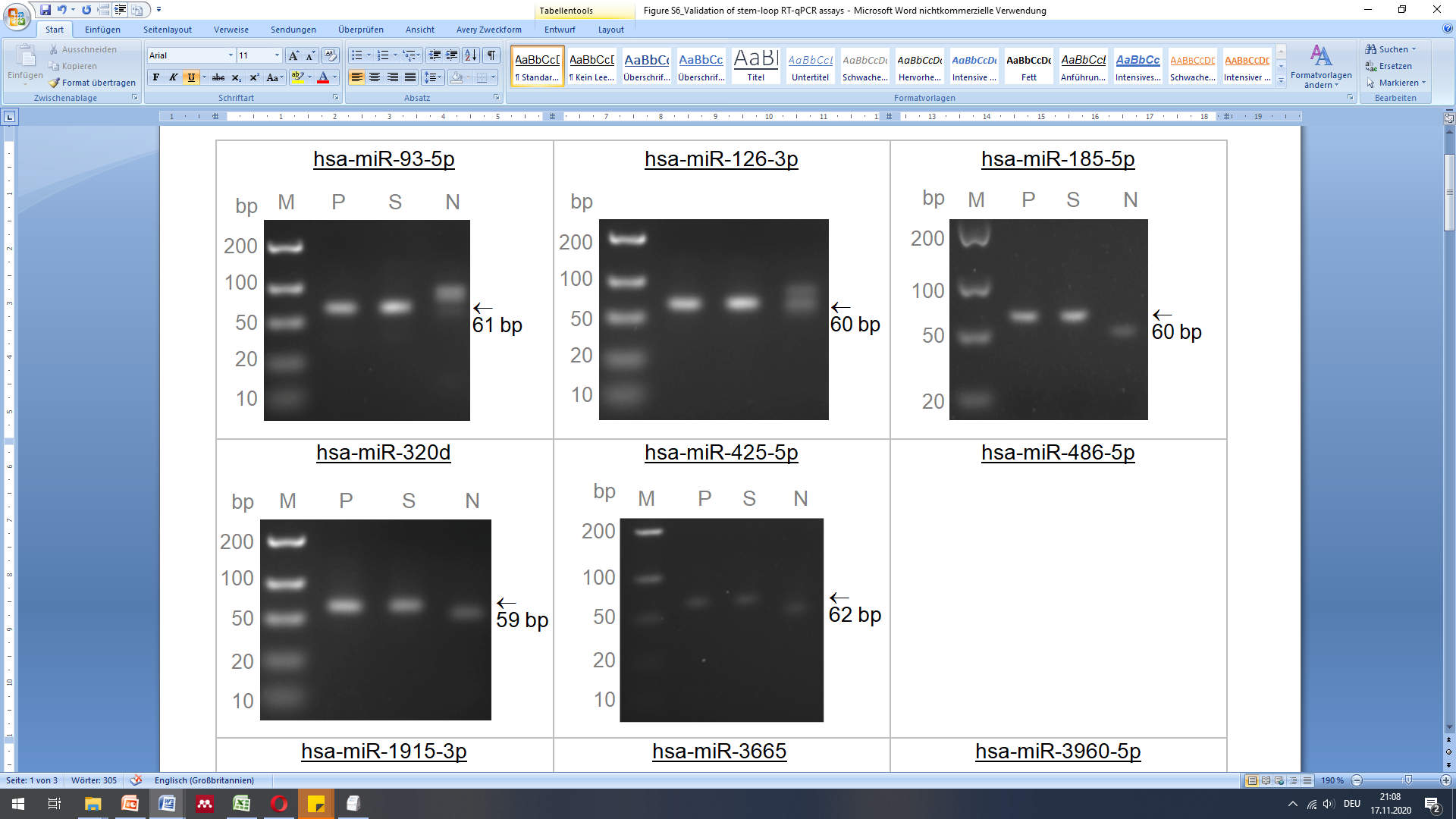


M P S N

TR146 RT+

TR146 RT-

Subject

NTC

**hsa-miR-185-5p** (MIMAT0000455)

TR146 RT+

TR146 RT-

Subject

NTC

Tm: 83.3°C


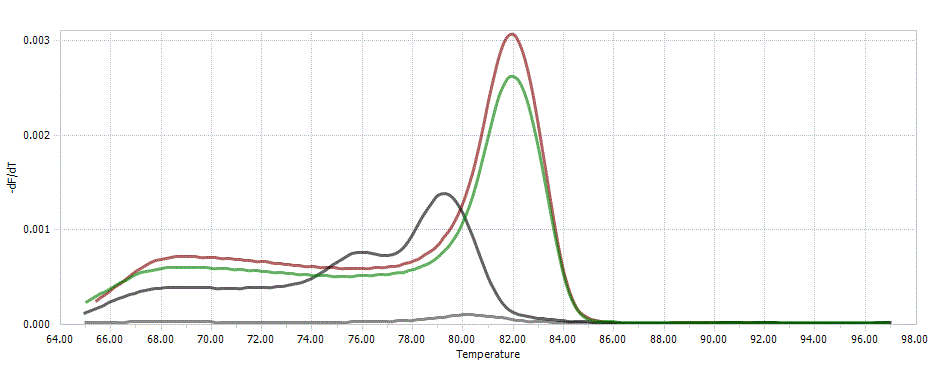

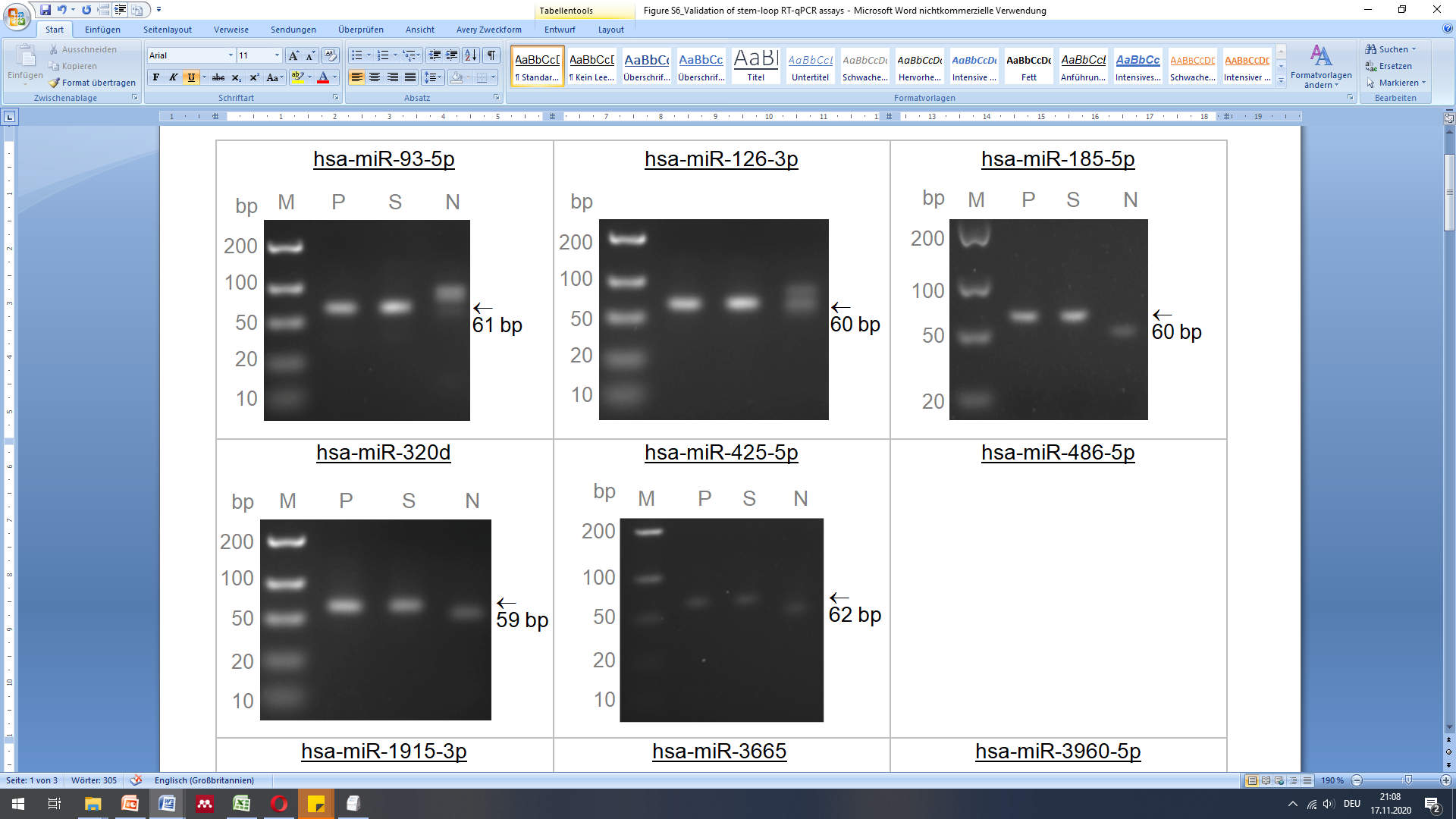


**hsa-miR-320d** (MIMAT0006764)

Tm: 82.3°C


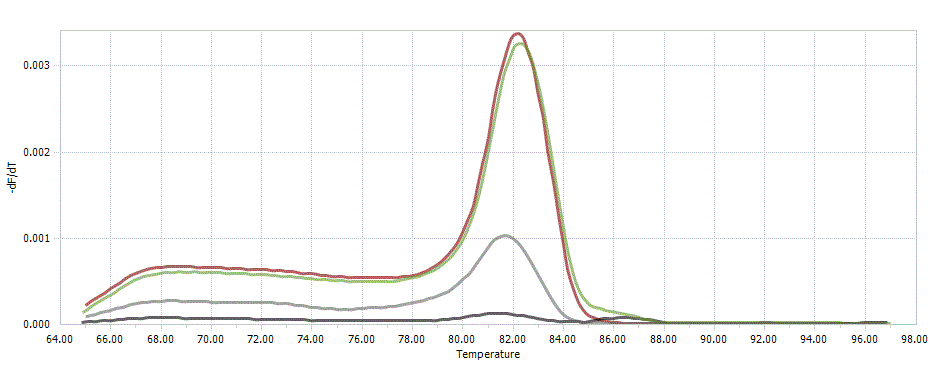

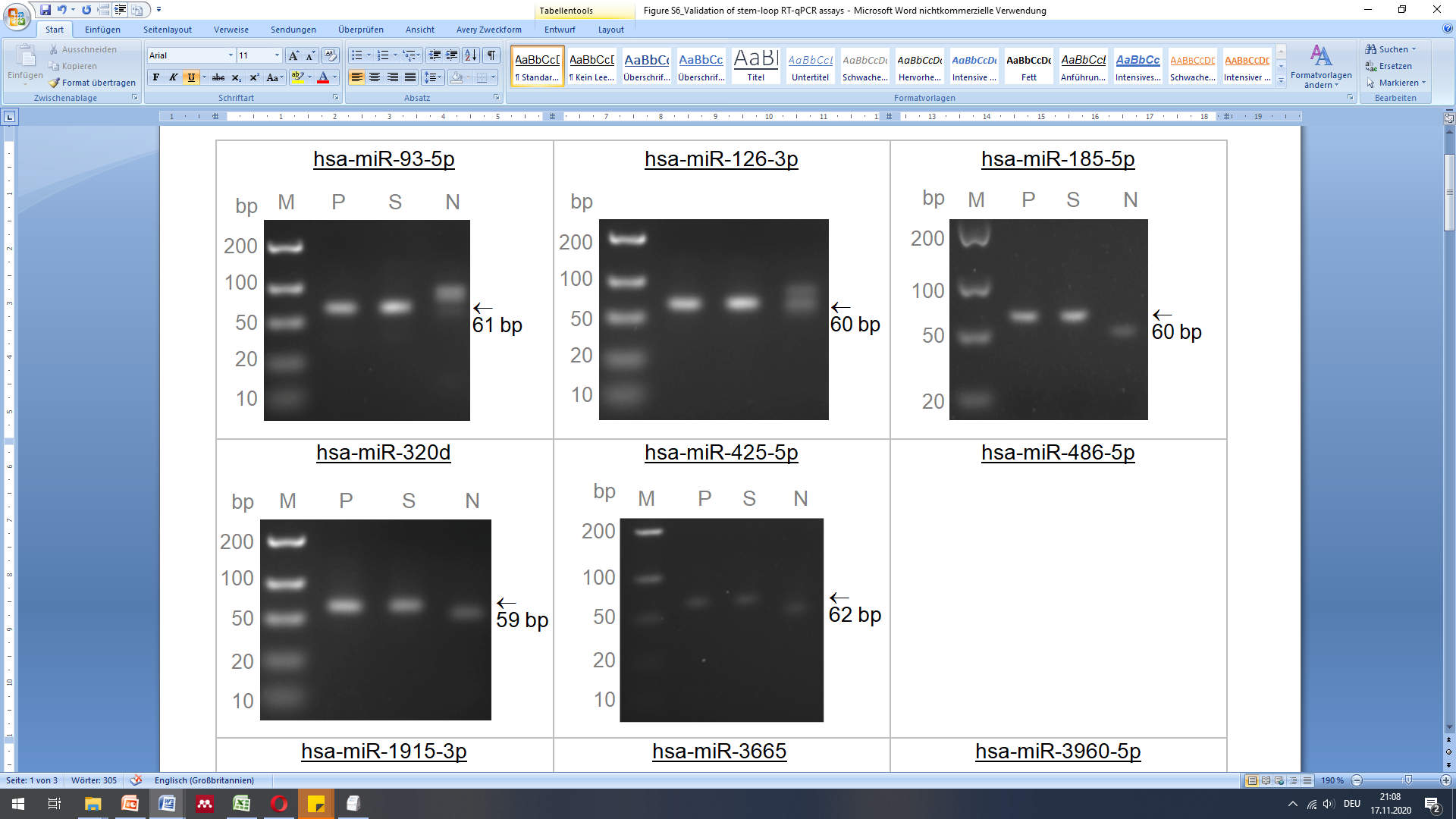


TR146 RT+

TR146 RT-

Subject

NTC

**hsa-miR-425-5p** (MIMAT0003393)

Tm: 82.5°C


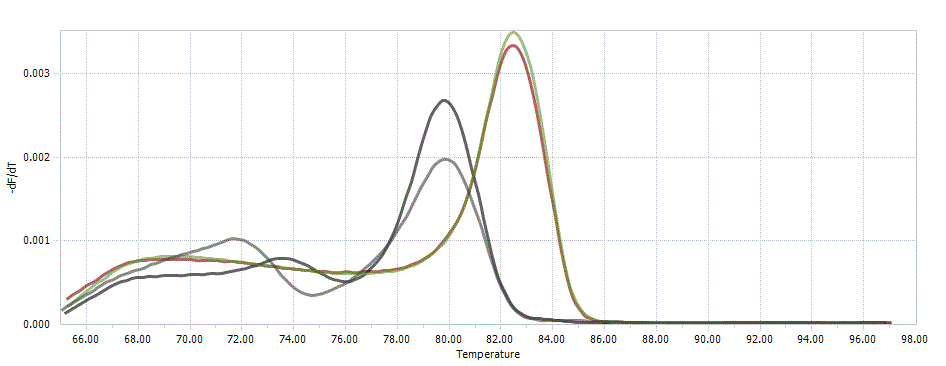

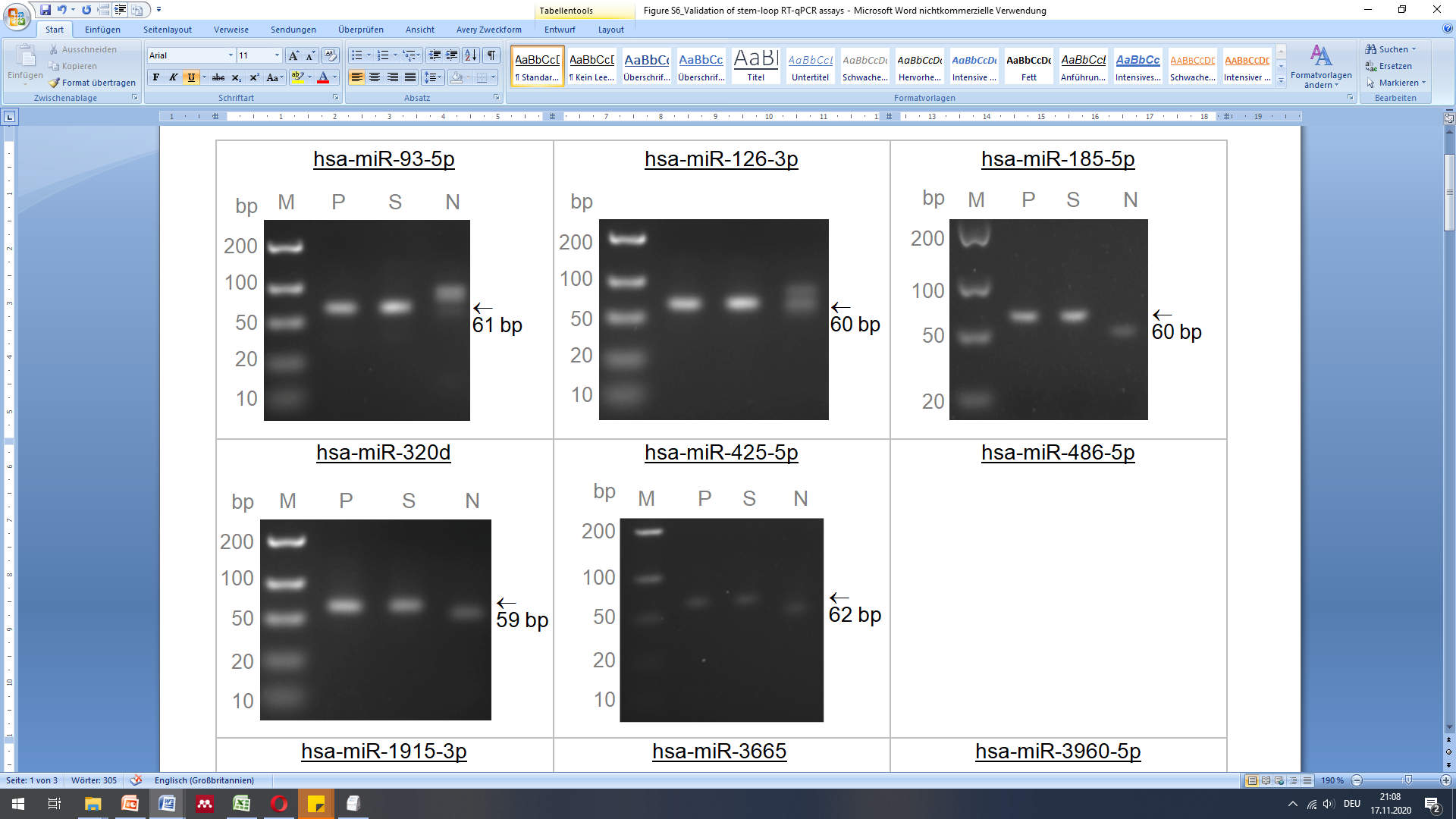


TR146 RT+

TR146 RT-

Subject

NTC

**hsa-miR-486-5p** (MIMAT0002177)

Tm: 84.6°C


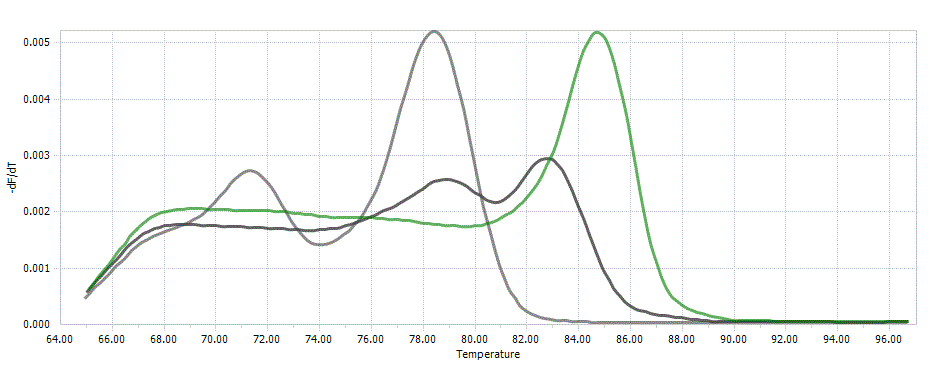

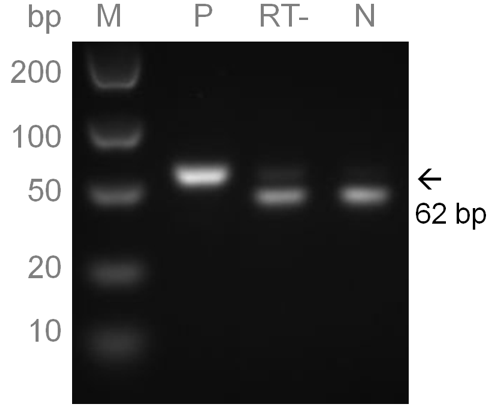


HepG2 RT+

HepG2 RT-

NTC

**hsa-miR-1915-3p** (MIMAT0007892)

Tm: 85.1°C


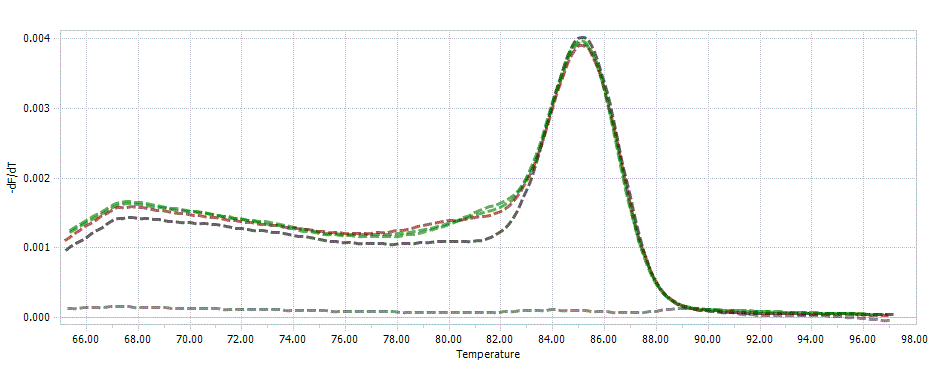

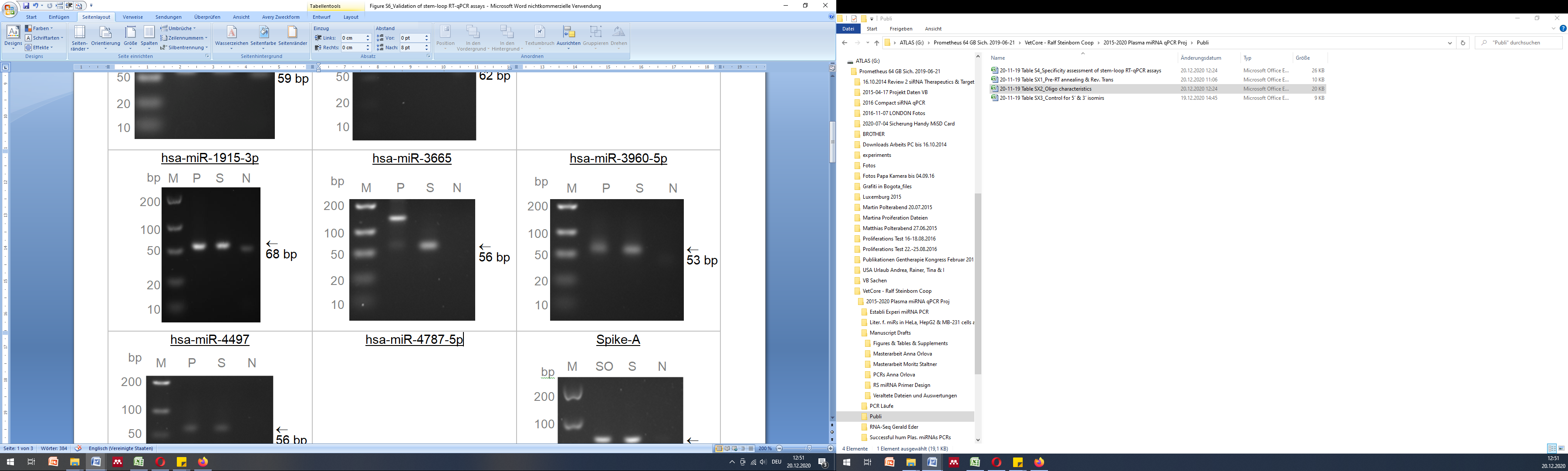


MDA-MB-231 RT+

HepG2 RT+

HepG2 RT-

Subject

NTC

**hsa-miR-3656** (MI0016056)

Tm: 86.3°C


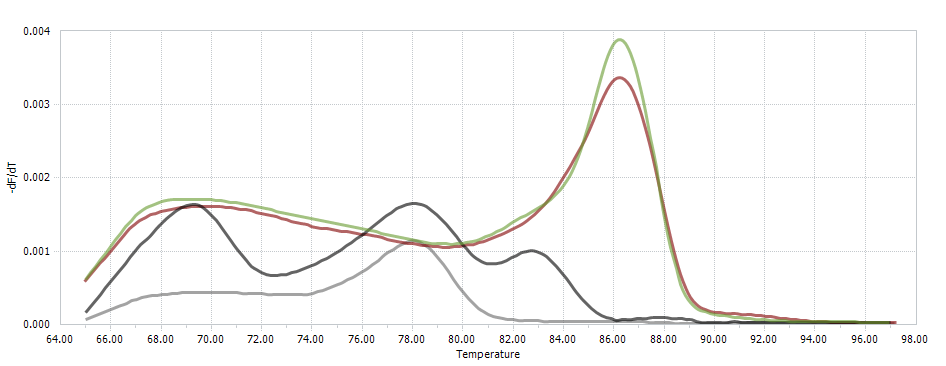

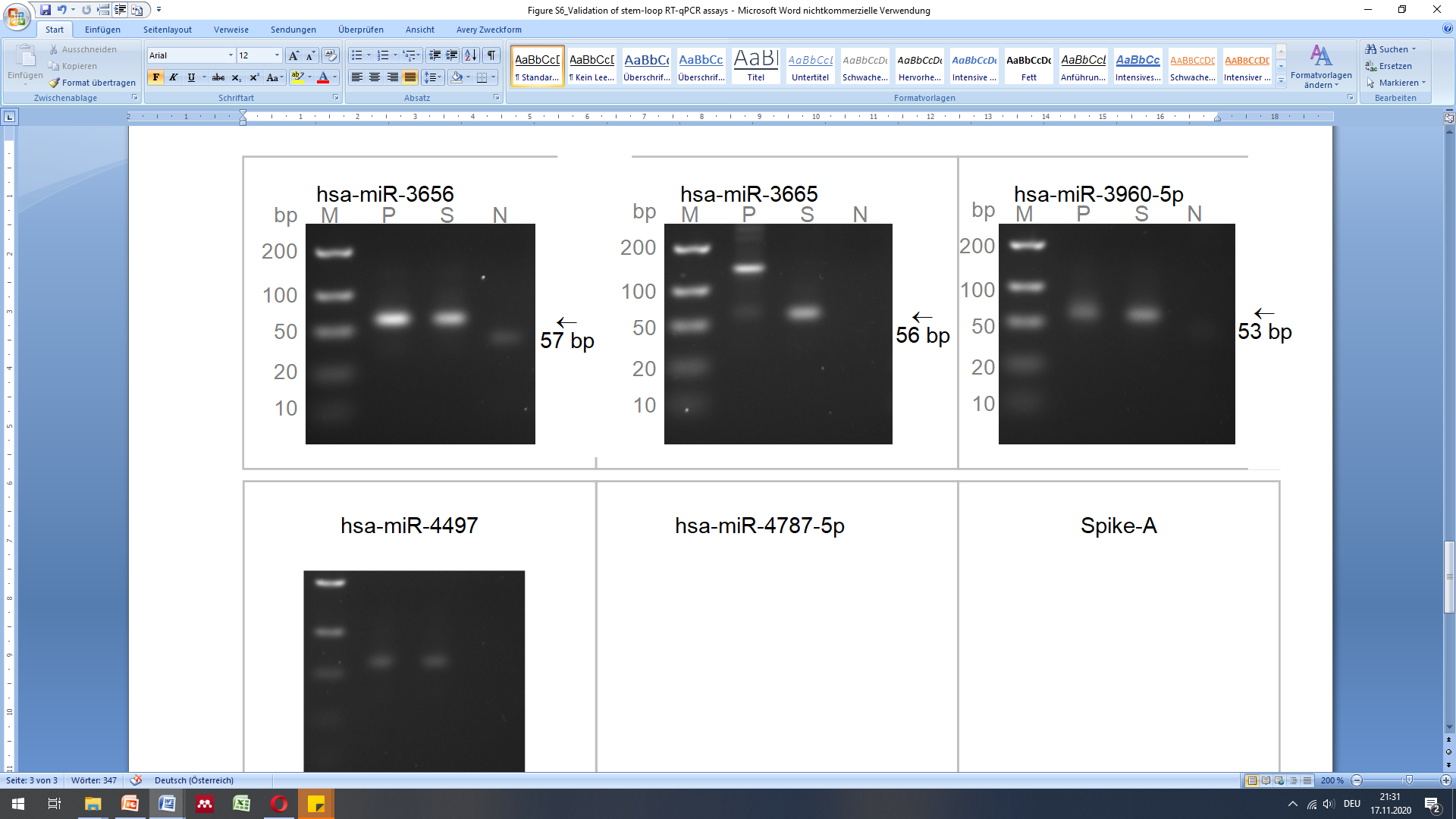


TR146 RT+

TR146 RT-

Subject

NTC

**hsa-miR-3665** (MIMAT0018087)

Tm: 86.6°C


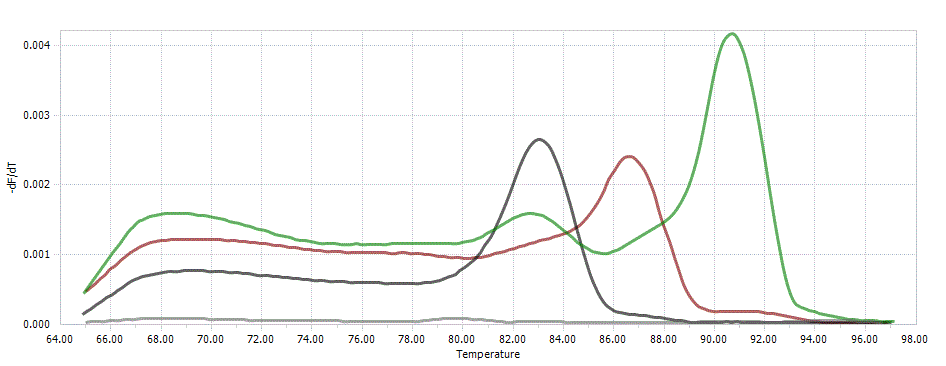

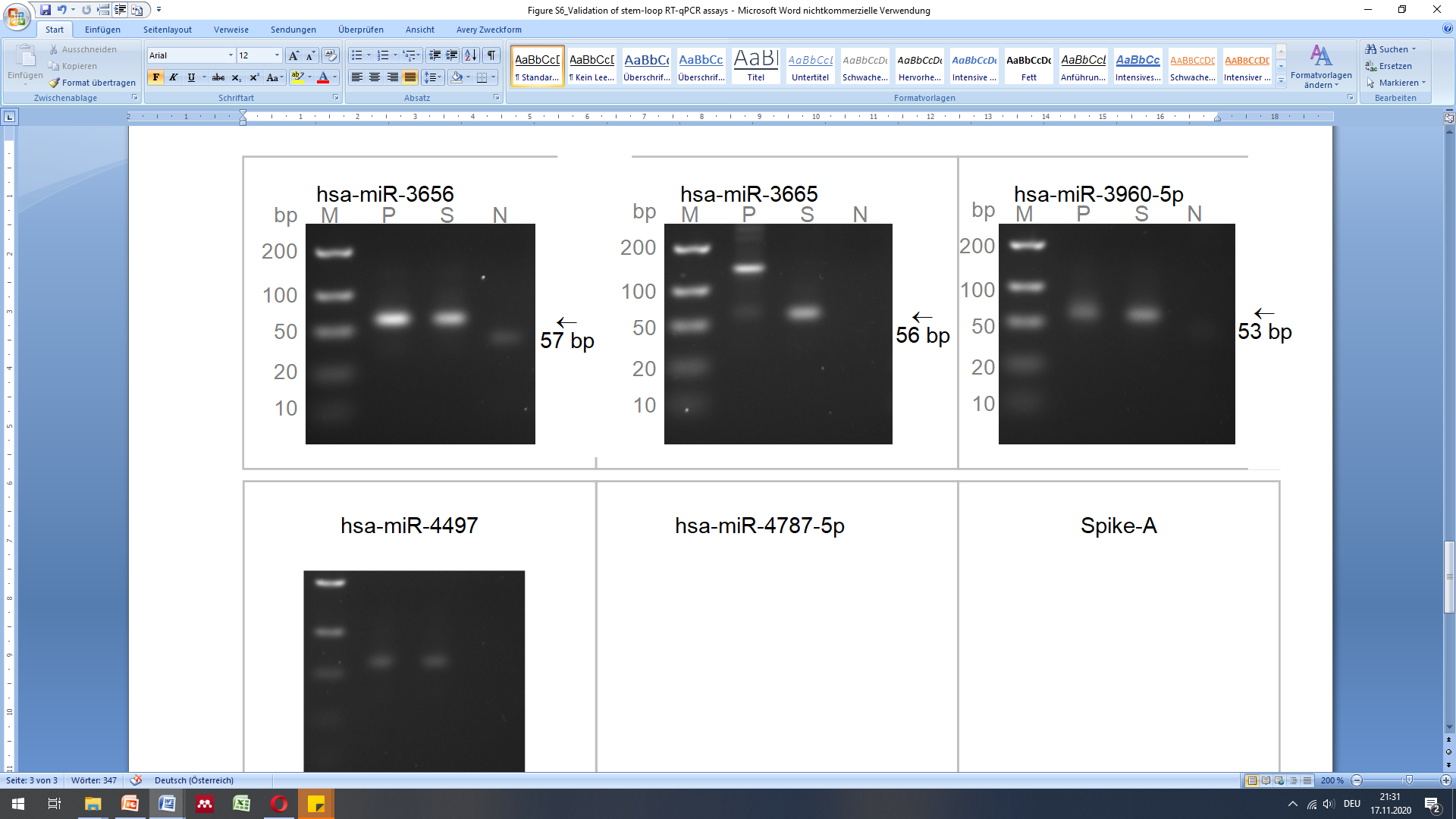


TR146 RT+

TR146 RT-

Subject

NTC

**hsa-miR-3960** (MIMAT0019337)

Tm: 84.6°C


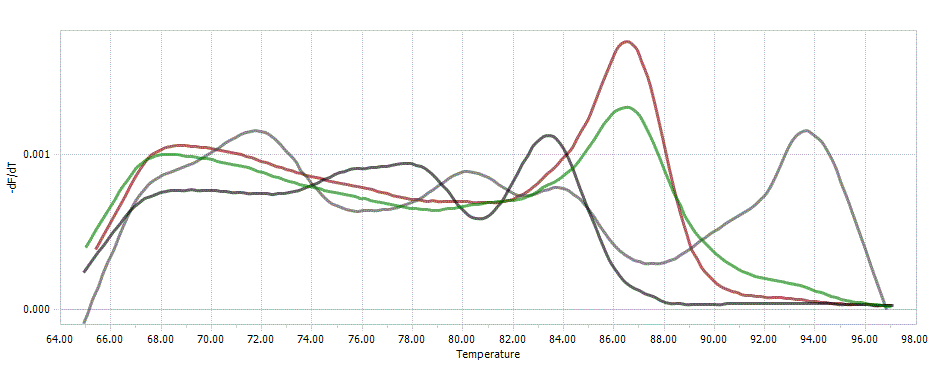

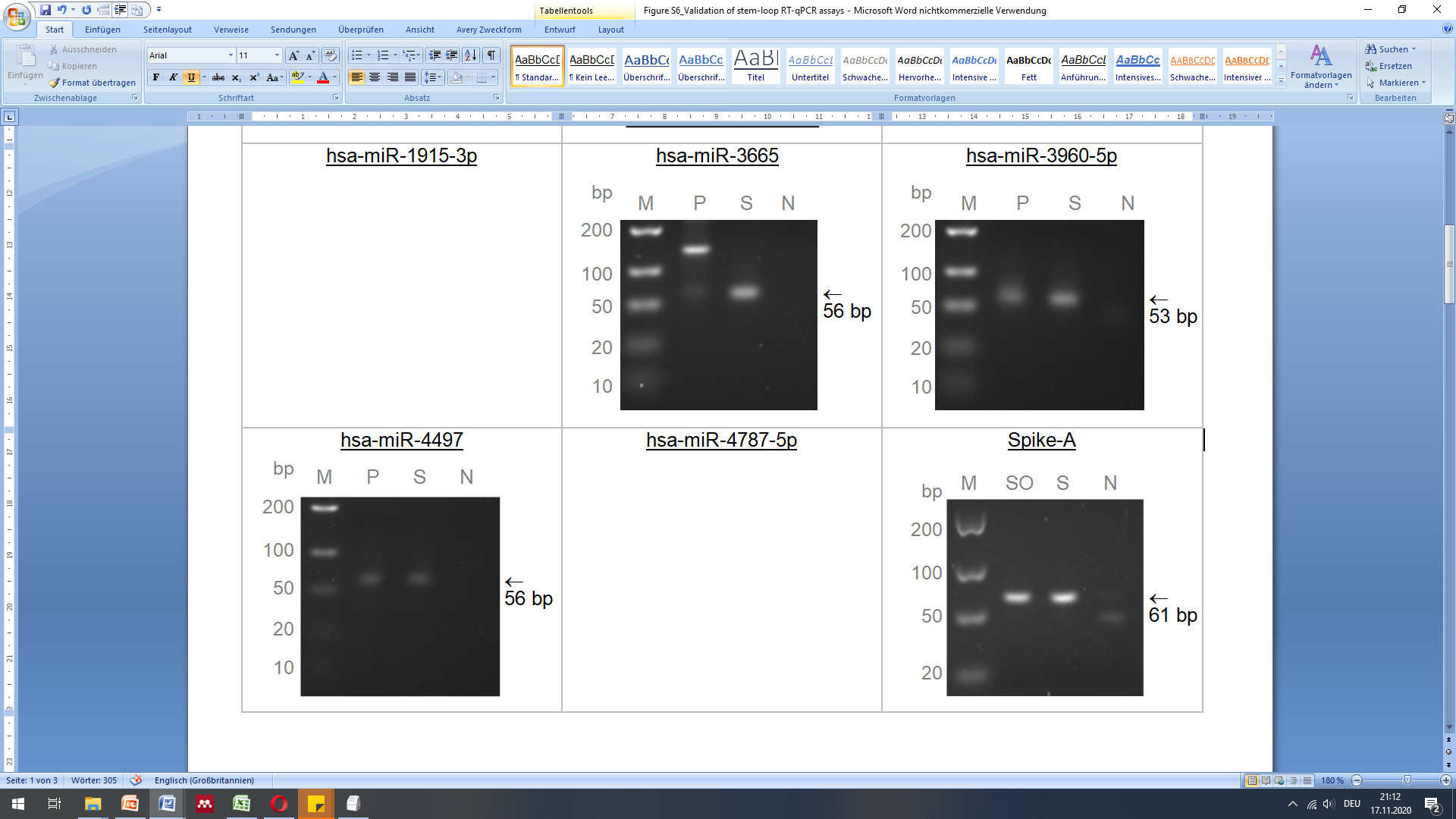


TR146 RT+

TR146 RT-

Subject

NTC

**hsa-miR-4488** (MIMAT0019022): not consistently detected by stem-loop RT-qPCR

Tm: 87.6°C


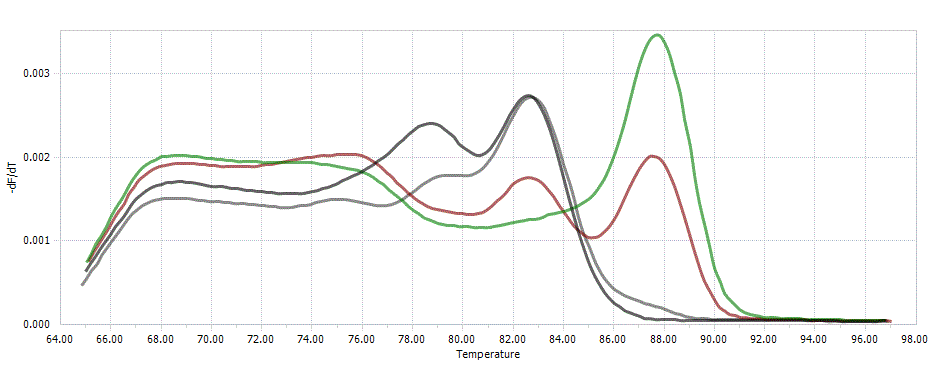

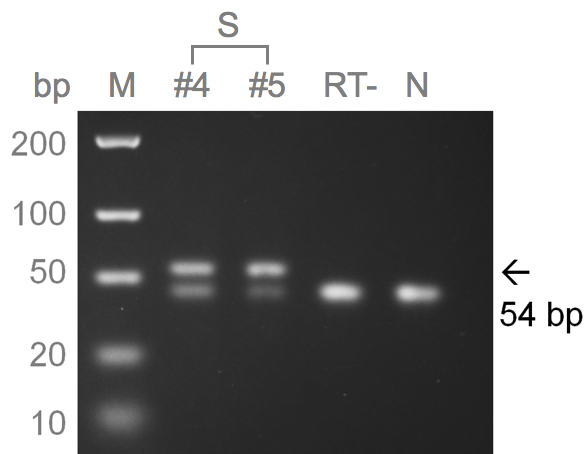


HepG2 RT+

HepG2 RT-

Subject 4

NTC

**hsa-miR-4497** (MIMAT0019032)

Tm: 86.0°C


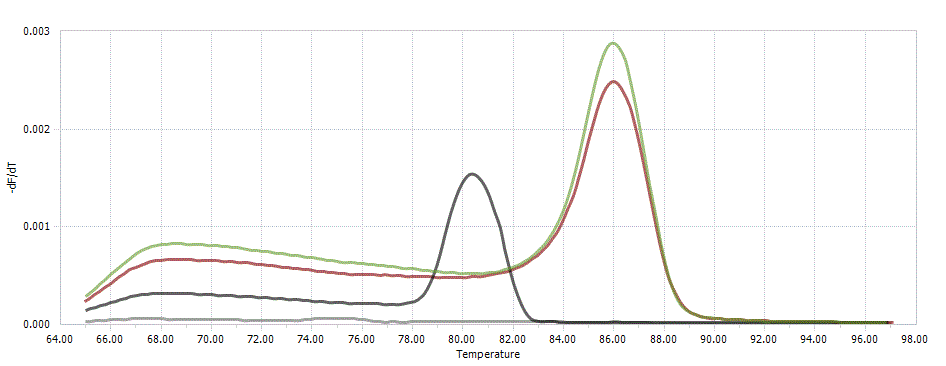

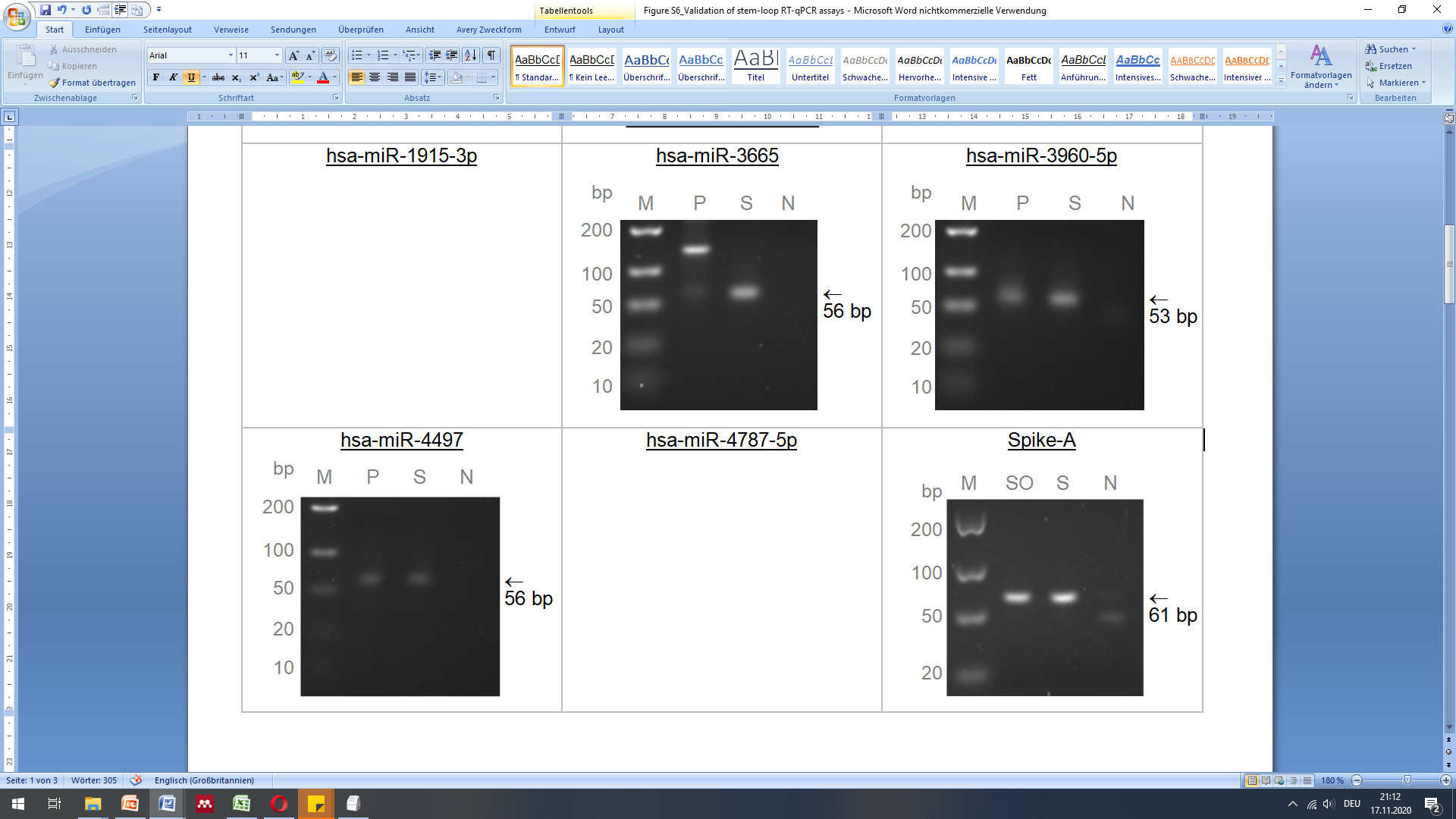


TR146 RT+

TR146 RT-

Subject

NTC

**hsa-miR-4787-5p** (MIMAT0019956)

Tm: 86.6°C


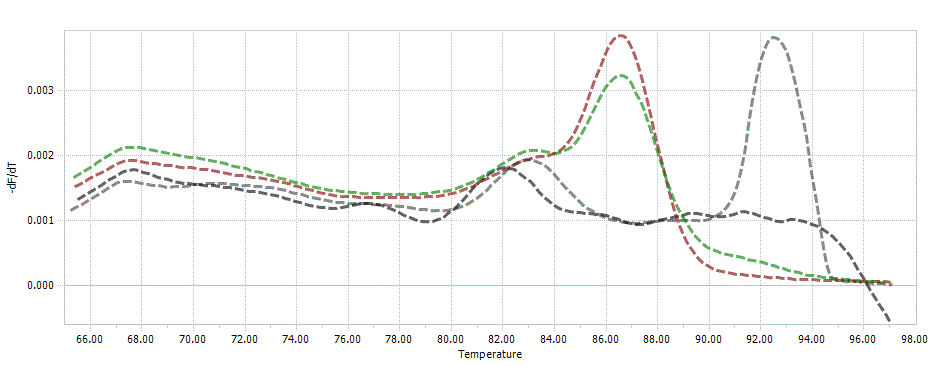

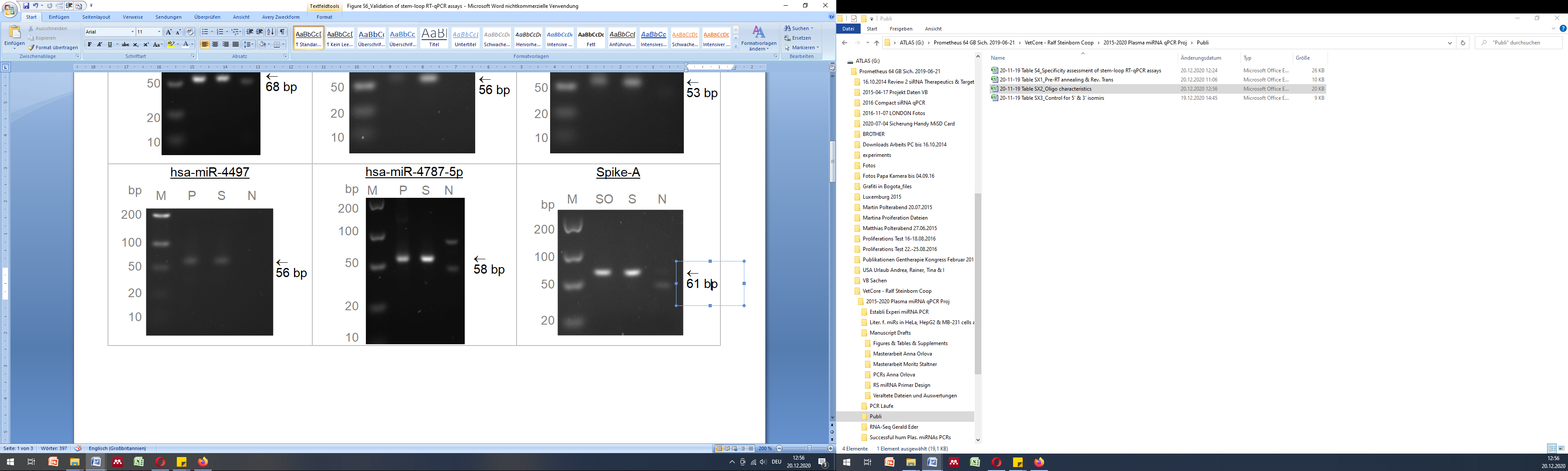


MDA-MB-231 RT+

MDA-MB-231 RT-

Subject

NTC

**Spike A** (Androvic PN *et al.* (2019) Two-tailed RT-qPCR panel for quality control of circulating microRNA studies. *Sci Rep* 9(1): 4255)

Tm: 84.5°C


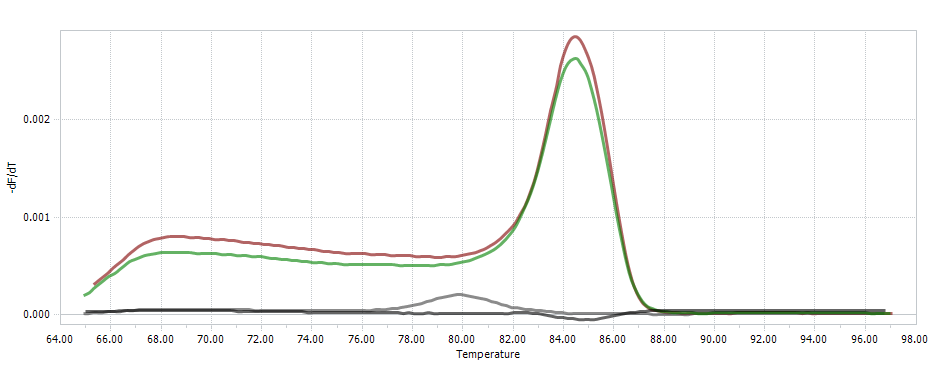

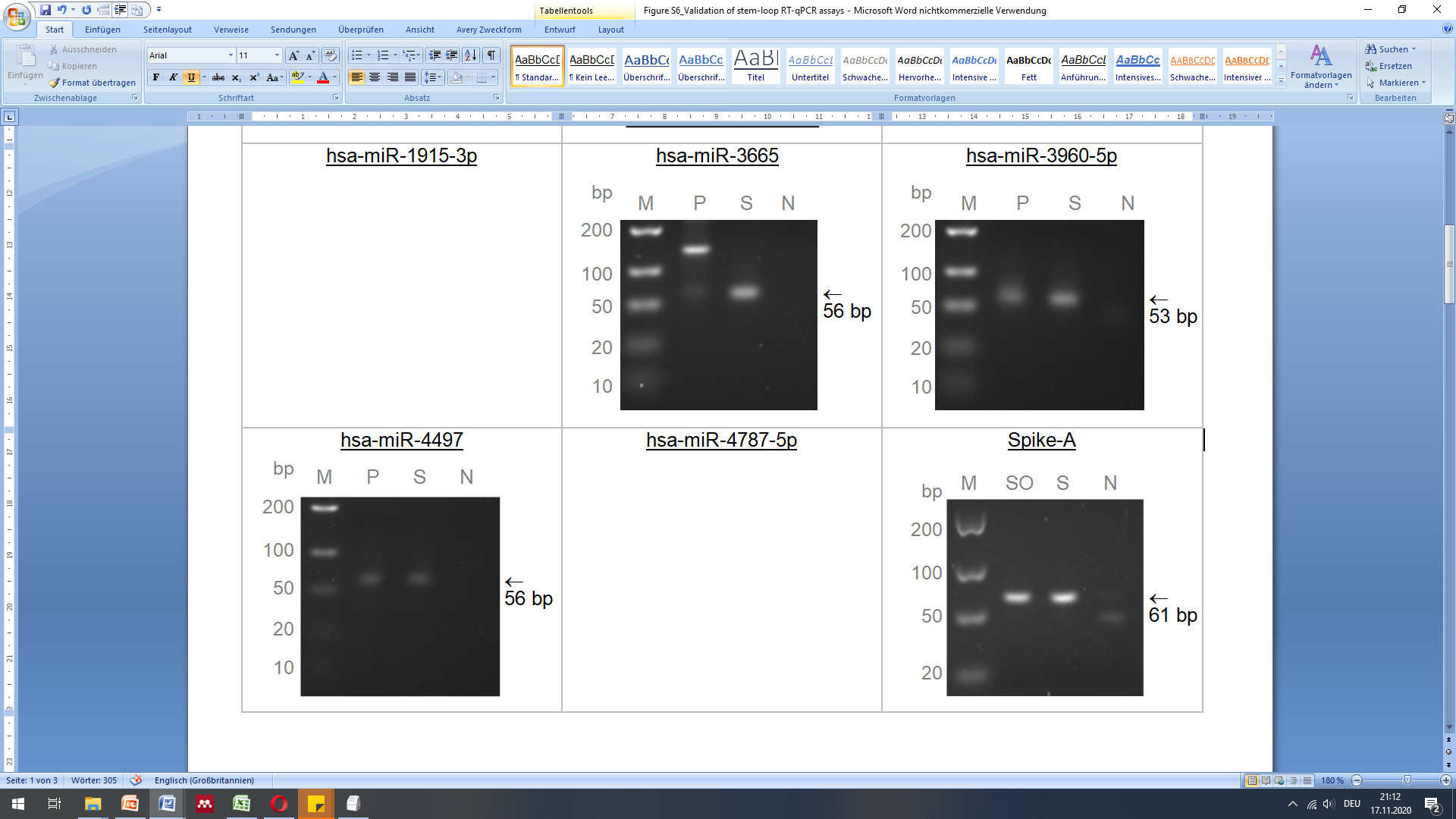


Spike A only RT+

Subject RT-

Subject

NTC

**Figure S3 |** Specificity assessment of stem-loop RT-qPCR amplicons using melt curve analysis (left) and agarose-gel electrophoresis (right). Positive controls for assay set-up: TR146 cells (nine assays), HepG2 cells (<https://www.atcc.org/products/hb-8065>) (three assays) or MDA-MB-231 cells (one assay).

RT+: positive control, RT-: mock RT, M: marker, S: subject (human plasma sample (spiked) from cohort of study phase-2), P: positive control, NTC or N: No-template control, SO: “Spike A” only RT+ control.

The procedure of electrophoretic separation is outlined in Supplementary File S1.
