# Supplementary material for: Identification of extremely GC-rich micro RNAs for RT-qPCR data normalization in human plasma
Source: Front Genet. 2023 Jan 4;13:1058668. doi: 10.3389/fgene.2022.1058668 (PMC9846067; doi:10.3389/fgene.2022.1058668)
Supplement: Supplementary file 1 [file DataSheet1.zip › Supporting information/Figure_S4_S-Poly(T) method designed for hsa-miR-1469.docx]

| 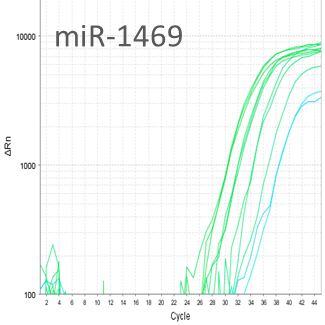 | 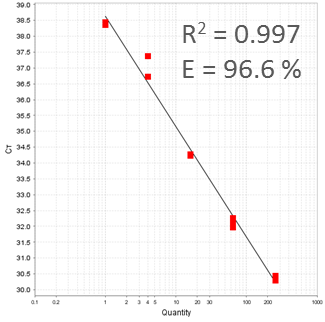 | 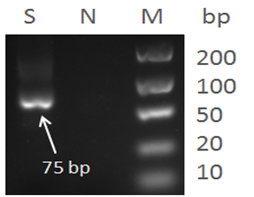 |
| --- | --- | --- |

**A B C**

**Figure S4 |** Performance of the S-Poly(T) method designed for hsa-miR-1469 that was consistently abundant in plasma of human males. (A) Duplicate qPCR plots of serial dilutions of a human plasma RNA sample. (B) Linear dynamic range and amplification efficiency (*E*) of the assay. The calibration curve was constructed from serial dilutions of a plasma RNA sample measured in duplicate. (C) Electrophoresis of the assay amplicon on a 2% agarose gel. The specific amplicon is indicated by the arrow. S: sample, N: “water control”, M: marker. The procedure of electrophoretic separation is outlined in Supplementary File S1.
