# Supplementary material for: Identification of extremely GC-rich micro RNAs for RT-qPCR data normalization in human plasma
Source: Front Genet. 2023 Jan 4;13:1058668. doi: 10.3389/fgene.2022.1058668 (PMC9846067; doi:10.3389/fgene.2022.1058668)
Supplement: Supplementary file 1 [file DataSheet1.zip › Supporting information/Figure_S5_GeNorm's pairwise variation (V) of putative normalizers.docx]

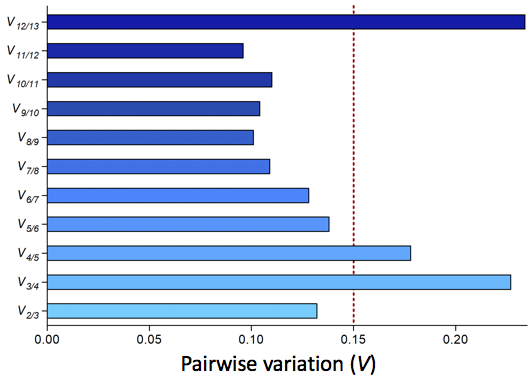


**Figure S5 |** Pairwise variation (*V*) of candidate genes determined by the GeNorm algorithm. The pairwise variation (*V_n_*/*V_n_* _+ 1_) was calculated between the normalization factors *NF_n_* and *NF_n_*_+ 1_. Each pairwise variation value is compared with the recommended cut-off of 0.15 (dotted line). Below this threshold value the inclusion of an additional reference gene would be optional.
