# Supplementary material for: Identification of extremely GC-rich micro RNAs for RT-qPCR data normalization in human plasma
Source: Front Genet. 2023 Jan 4;13:1058668. doi: 10.3389/fgene.2022.1058668 (PMC9846067; doi:10.3389/fgene.2022.1058668)
Supplement: Supplementary file 1 [file DataSheet1.zip › Supporting information/Figure_S6_miRNAs composing the two most stable NFs.docx]

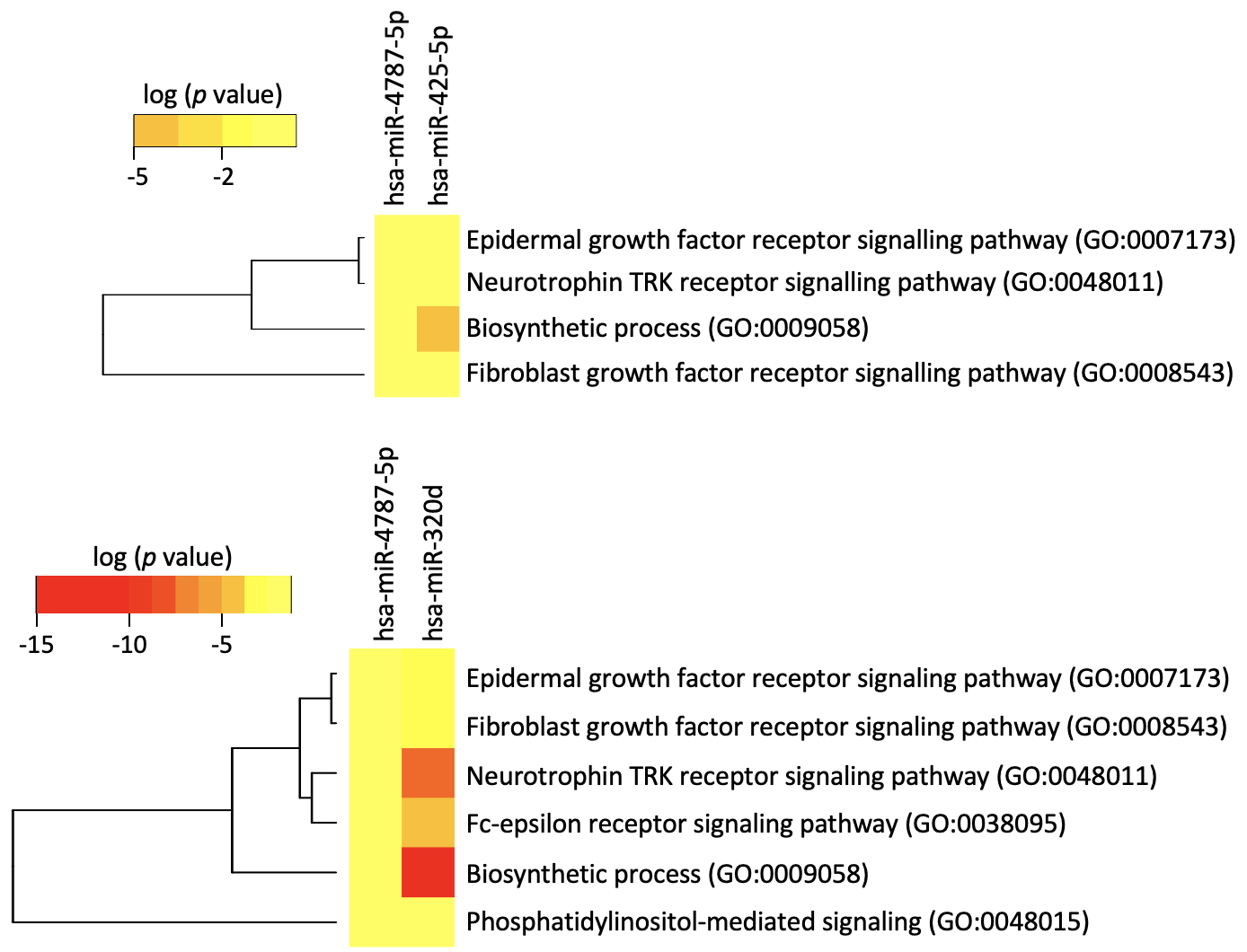


**Figure S6 |** The miRNAs composing the two most stable *NF*s are involved in different signalling or metabolic pathways that only occasionally overlap. The dendrogram depicts hierarchical clustering results for the GO pathways targeted by at least one of the miRNAs of a pair. The extend of targeting is signified by the *p* value with darker colour representing higher significance (see colour key of heatmap).

GO: Gene Ontology; *NF*: normalization factor. The pathway’s GO identity is bracketed. For miRNAs with very high GC content such as miR-4787-5p (top and bottom illustrations), less experimental support, hence, lower statistical significance should be considered ([1] and this study).

Reference

1. Nersisyan S, Shkurnikov M, Poloznikov A, Turchinovich A, Burwinkel B, Anisimov N, Tonevitsky A: **A Post-Processing Algorithm for miRNA Microarray Data**. *Int J Mol Sci* 2020, **21**(4):1228. doi: 10.3390/ijms21041228.
