# Supplementary material for: Identification of extremely GC-rich micro RNAs for RT-qPCR data normalization in human plasma
Source: Front Genet. 2023 Jan 4;13:1058668. doi: 10.3389/fgene.2022.1058668 (PMC9846067; doi:10.3389/fgene.2022.1058668)
Supplement: Supplementary file 1 [file DataSheet1.zip › Supporting information/File_S1_Extended_Methods.DOCX]

**Supplementary File S1**: Extended Methods section.

**Cell culture and extraction of cell-line RNA**

RT-qPCR assays were established on templates isolated from the human cancer-derived cell lines TR146, HepG2 and MDA-MB-231. Nine assays were set-up using TR146 cells derived from buccal carcinoma ((Rupniak *et al.* 1985); catalogue number 10032305-1VL; Sigma-Aldrich, Vienna Austria; [https://web.expasy.orGCellosaurus/CVCL_2736](https://web.expasy.org/cellosaurus/CVCL_2736)). Hepatocellular carcinoma HepG2 cells (<https://www.atcc.org/products/hb-8065>) and breast adenocarcinoma MDA-MB-231 cells (<https://www.atcc.org/products/crm-htb-26>) were used for three and one assays, respectively.

Cells were grown in monolayer up to an estimated density of ~ 5 to 10 × 10^6^ cells per 25-cm^2^ flask, washed twice with 1 × phosphate-buffered saline (PBS) buffer (137 mM NaCl, 2.7 mM KCl, 8 mM Na_2_HPO_4_, and 2 mM KH_2_PO_4_, pH 7.4), detached with 1 ml 0.025% trypsin supplemented with 1 mM ethylenediaminetetraacetic acid (EDTA) for 3 to 5 min at 37°C, and resuspended in 5 ml of PBS buffer. The suspension was aliquoted and centrifuged at 750× *g* for 3 min at room temperature. After removing the supernatant, pellets containing ~ 0.5 to 1 × 10^6^ cells were stored at –20°C for RNA extraction within ≤ 30 days or at –70°C for longer time to facilitate cell-line authentication by DNA genotyping.

An aliquot containing 5 × 10^5^ frozen cells was subjected to RNA extraction by acid guanidinium thiocyanate-phenol-chloroform extraction using 1 ml TRI Reagent (Zymo Research, Irvine, CA, USA). The lysate was homogenised by pipetting up and down and transferred into a pre-spun 1.5-ml Invitrogen™ Phasemaker™ Tube (Thermo Fisher Scientific; (Fuentes-Iglesias *et al.* 2020)) and further processed according to the instruction of the manufacturer. RNA was resuspended in 30 µl RNase-free water, incubated at 55°C for 5 min and stored at –80°C until further use. RNA concentration and purity were determined using the DeNovix DS-11 FX+ Spectrophotometer (DeNovix, Wilmington, DE, USA).

**Cell-line authentication**

The cell line TR146 used to set-up most of the assays was authenticated by short tandem repeat (STR) analysis. A pellet containing 5 × 10^6^ TR146 cells was washed twice in 1 × PBS buffer, resuspended in 0.5 ml 80% ethanol and shipped by regular mail to a service provider for STR DNA Profiling Analysis (Microsynth AG, Wolfurt-Bahnhof, Austria). Loci were amplified using a multiplex STR system for use in DNA typing (PowerPlex^®^ 16 HS System; Promega, Madison, WI, USA). Fragments were separated by capillary electrophoresis on the Applied Biosystems™ 3730xl 96-capillary DNA Analyzer (Thermo Fisher Scientific). Genotypes were derived from the resulting data using the GeneMarker^®^ HID human identity software (SoftGenetics, State College, PA, USA). To allow a certain degree of heterozygosity gains and/or losses that might accumulate during passaging, at least 80% of the STR alleles were requested to match the reference (Capes-Davis *et al.* 2013). The proportion of matching alleles was calculated by dividing the number of shared alleles by the number of total alleles.

**Gel electrophoresis**

For size confirmation of qPCR amplicons, 4 µl of product was mixed with 2 μl of 6 × Orange DNA Loading Dye (Thermo Fisher Scientific) and loaded onto a 2% (weight/volume) agarose gel prepared in 1 × sodium-borate conductive medium (Brody and Kern 2004) and precast-stained with GelGreen™ Nucleic Acid Stain (Biotium, Hayward, CA, USA). Poor resolution and band artifacts resulting from current and asymmetric heating effects were minimised by running the gel at low voltage (9.0 V/cm). Product size was assessed using the FastRuler Ultra Low Range DNA Ladder (Thermo Fisher Scientific).

**Coefficient of variation (*CV*) analysis**

For determination of the *CV* (ratio between standard deviation and mean), *Cq* values were converted into relative quantities (Hellemans *et al.* 2007), also termed linear scale transformation (linearisation) of *Cq* values (2^–^*^Cq^*) (Marabita *et al.* 2016; Sundaram *et al.* 2019). In detail, the efficiency-adjusted *Cq* values were transformed into *n*-fold quantities relatively to lowest expressing sample according to the term $2^{maximum Cq - sample Cq}$. Note that using real numbers for *CV* calculation results in a profound difference compared to just *Cq* values (Kralik and Ricchi 2017) and renders this measure of variation/stability independent of the actual level of target abundance.

**Bioinformatical details**

Identity numbers of the mature miRNAs and their chromosomal locations were retrieved from miRBase release 22 ((Kozomara and Griffiths-Jones 2014), <https://www.mirbase.org>). Information on miRNA subtype— intergenic, intronic or exonic was obtained from the miRIAD database ((Hinske *et al.* 2014); <https://bmi.ana.med.uni-muenchen.de/miriad/>). Information on miRNA shuttles was retrieved from The Extracellular Vesicles miRNA Database (EVmiRNA; (Liu *et al.* 2019); <http://bioinfo.life.hust.edu.cn/EVmiRNA/#!/>), Vesiclepedia ((Pathan *et al.* 2019); <http://microvesicles.org/index.html>), or the peer-reviewed literature. Information on miRNA orthologues was obtained from miRBase (<https://www.mirbase.org>), RNAcentral release 19 ((Consortium 2021); <https://rnacentral.org>) and The Alliance of Genome Resources version 5.2.1 ((Alliance of Genome Resources 2022); <https://www.alliancegenome.org>). The potential of mature miRNAs to form intramolecular structures was predicted at 37°C using the ViennaRNA Package version 2.4.18 (Lorenz *et al.* 2011) implemented in the RNAFold server (<http://rna.tbi.univie.ac.at/cgi-bin/RNAWebSuite/RNAfold.cgi>).

**Target prediction and enrichment analysis of gene ontology (GO) terms**

Putative targets of the stable miRNAs were predicted using the bioinformatic tool microRNA Data Integration Portal mirDIP 4.1 (Tokar *et al.* 2018); <http://ophid.utoronto.ca/mirDIP/>) run with modification implemented in November 2020 (version 4.1.11.2, database version 4.1.0.3). In particular, the tool integrated human RNA-gene target predictions contained in 30 miRNA prediction databases, thus obtaining more robust data about the miRNA-mRNA interaction levels with a minimised database-specific bias. The pathways and larger processes to which a certain gene product’s activity contributes, *i.e.*, the biological processes, were retrieved from the GO project (Carbon *et al.* 2017) using QuickGO (EMBL-EBI, part of the European Molecular Biology Laboratory, Wellcome Genome Campus, Hinxton, UK; <https://www.ebi.ac.uk/QuickGO/>). If a biological process GO term was not yet assigned to a gene, its pathway classification was obtained from PathCards, a multi-source consolidation of human biological pathways ((Belinky *et al.* 2015); <https://pathcards.genecards.org>).

Insight into the functional interconnection of the ten most likely miRNA targets was obtained from GO term enrichment analysis. The scanning for GO categories overrepresented in the input list was performed with the open-source web-application GOnet ((Pomaznoy *et al.* 2018); <https://tools.dice-database.org/GOnet/>) using the GO namespace “biological_process”, UniProt IDs as input and a *q* value — a corrected *p* value calculated using the false-discovery rate — of ≤ 0.05.

Functionality of the miRNAs predicted as the most stable *NF* combination was assessed using the miRNA Pathway Dictionary Database (miRPathDB) release 2.0 ((Kehl *et al.* 2020); <https://mpd.bioinf.uni-sb.de/>).

The biological functionality of the miRNA pair composing the *NF* for an experimental context was evaluated at DIANA-mirPath v3.0, a web-server for miRNA pathway analysis ((Vlachos *et al.* 2015); <https://dianalab.e-ce.uth.gr/html/mirpathv3/index.php?r=mirpath>). Interactions were predicted by DIANA’s microT-CDS (version 5.0) database. Significantly enriched GO terms were obtained by Fisher’s exact test followed by Benjamini-Hochberg *post hoc* correction (thresholds: *p* < 0.05, microT = 0.8).

**References**

Alliance of Genome Resources, C., 2022 Harmonizing model organism data in the Alliance of Genome Resources. Genetics 220**:** iyac022.

Belinky, F., N. Nativ, G. Stelzer, S. Zimmerman, T. Iny Stein *et al.*, 2015 PathCards: multi-source consolidation of human biological pathways. Database (Oxford) 2015.

Brody, J. R., and S. E. Kern, 2004 Sodium boric acid: a Tris-free, cooler conductive medium for DNA electrophoresis. BioTechniques 36**:** 214-216.

Carbon, S., H. Dietze, S. E. Lewis, C. J. Mungall, M. C. Munoz-Torres *et al.*, 2017 Expansion of the Gene Ontology knowledgebase and resources. Nucleic Acids Research 45**:** D331-D338.

Consortium, R., 2021 RNAcentral 2021: secondary structure integration, improved sequence search and new member databases. Nucleic Acids Res 49**:** D212-D220.

Fuentes-Iglesias, A., V. Garcia-Outeiral, J. A. Pardavila, J. Wang, M. Fidalgo *et al.*, 2020 An Optimized Immunoprecipitation Protocol for Assessing Protein-RNA Interactions In Vitro. STAR Protoc 1.

Hellemans, J., G. Mortier, A. De Paepe, F. Speleman and J. Vandesompele, 2007 qBase relative quantification framework and software for management and automated analysis of real-time quantitative PCR data. Genome Biol 8**:** R19.

Hinske, L. C., G. S. Franca, H. A. Torres, D. T. Ohara, C. M. Lopes-Ramos *et al.*, 2014 miRIAD-integrating microRNA inter- and intragenic data. Database (Oxford) 2014.

Kehl, T., F. Kern, C. Backes, T. Fehlmann, D. Stockel *et al.*, 2020 miRPathDB 2.0: a novel release of the miRNA Pathway Dictionary Database. Nucleic Acids Res 48**:** D142-D147.

Kozomara, A., and S. Griffiths-Jones, 2014 miRBase: annotating high confidence microRNAs using deep sequencing data. Nucleic Acids Research 42**:** D68-D73.

Kralik, P., and M. Ricchi, 2017 A Basic Guide to Real Time PCR in Microbial Diagnostics: Definitions, Parameters, and Everything. Front Microbiol 8**:** 108.

Liu, T., Q. Zhang, J. Zhang, C. Li, Y. R. Miao *et al.*, 2019 EVmiRNA: a database of miRNA profiling in extracellular vesicles. Nucleic Acids Res 47**:** D89-D93.

Lorenz, R., S. H. Bernhart, C. Höner zu Siederdissen, H. Tafer, C. Flamm *et al.*, 2011 ViennaRNA Package 2.0. Algorithms for Molecular Biology 6**:** 26.

Marabita, F., P. de Candia, A. Torri, J. Tegner, S. Abrignani *et al.*, 2016 Normalization of circulating microRNA expression data obtained by quantitative real-time RT-PCR. Briefings in Bioinformatics 17**:** 204-212.

Pathan, M., P. Fonseka, S. V. Chitti, T. Kang, R. Sanwlani *et al.*, 2019 Vesiclepedia 2019: a compendium of RNA, proteins, lipids and metabolites in extracellular vesicles. Nucleic Acids Res 47**:** D516-D519.

Pomaznoy, M., B. Ha and B. Peters, 2018 GOnet: a tool for interactive Gene Ontology analysis. BMC Bioinformatics 19**:** 470.

Rupniak, H. T., C. Rowlatt, E. B. Lane, J. G. Steele, L. K. Trejdosiewicz *et al.*, 1985 Characteristics of four new human cell lines derived from squamous cell carcinomas of the head and neck. J Natl Cancer Inst 75**:** 621-635.

Sundaram, V. K., N. K. Sampathkumar, C. Massaad and J. Grenier, 2019 Optimal use of statistical methods to validate reference gene stability in longitudinal studies. PLoS One 14**:** e0219440.

Tokar, T., C. Pastrello, A. E. M. Rossos, M. Abovsky, A. C. Hauschild *et al.*, 2018 mirDIP 4.1-integrative database of human microRNA target predictions. Nucleic Acids Res 46**:** D360-D370.

Vlachos, I. S., K. Zagganas, M. D. Paraskevopoulou, G. Georgakilas, D. Karagkouni *et al.*, 2015 DIANA-miRPath v3.0: deciphering microRNA function with experimental support. Nucleic Acids Res 43**:** W460-466.
