# Supplementary material for: Identification of extremely GC-rich micro RNAs for RT-qPCR data normalization in human plasma
Source: Front Genet. 2023 Jan 4;13:1058668. doi: 10.3389/fgene.2022.1058668 (PMC9846067; doi:10.3389/fgene.2022.1058668)
Supplement: Supplementary file 1 [file DataSheet1.zip › Supporting information/Table_S1_Samples_of_microarray_analysis.docx]

**Table S1 |** Samples of study phase 1: templates for miRNA microarray analysis.

| **Microarray** | **MCI group** | **ID of proband(s) (single or pooled sample*)** |
| --- | --- | --- |
| 1 | non-defensive | 990306, 991146 |
| 2 | non-defensive | 990546 |
| 3 | non-defensive | 991226 |
| 4 | repressor | 990016, 991216 |
| 5 | repressor | 990586, 990796 |
| 6 | repressor | 991216, 991276 |
| 7 | high-anxious | 990316 |
| 8 | high-anxious | 990316, 991186 (2:1) |
| 9 | high-anxious | 991046, 991186 (2:1) |
| 10 | sensitizer | 990256, 990416, 991116 |
| 11 | sensitizer | 990366, 990436, 990616 |
| 12 | sensitizer | 990656, 991326 |

MCI: coping-style classification based on the responses to the cognitive avoidance and vigilance scales of the Mainz Coping Inventory (MCI) (Krohne 1989).

*Samples were pooled before RNA isolation.

In brackets: dilution factor

**Reference**

Krohne, H. W., 1989 The concept of coping modes: Relating cognitive person variables to actual coping behavior. Advances in Behaviour Research and Therapy 11**:** 235-248.
