# Supplementary material for: Identification of extremely GC-rich micro RNAs for RT-qPCR data normalization in human plasma
Source: Front Genet. 2023 Jan 4;13:1058668. doi: 10.3389/fgene.2022.1058668 (PMC9846067; doi:10.3389/fgene.2022.1058668)
Supplement: Supplementary file 1 [file DataSheet1.zip › Supporting information/Table_S12_Descriptive_statistics_for_miRNA_abundances_measured_by_stem-loop_RT-qPCR..DOCX]

**Table S12 |** Descriptive statistics for miRNA abundances measured by stem-loop RT-qPCR.

| Statistical category | miR-93-5p | miR-126-3p | miR-185-5p | miR-320d | miR-425-5p | miR-486-5p | miR-1915-3p | miR-3656 | miR-3665 | miR-3960 | miR-4497 | miR-4787-5p | Spike-A |
| --- | --- | --- | --- | --- | --- | --- | --- | --- | --- | --- | --- | --- | --- |
| Arithmetic *Cq* mean | 20.7 | 21.9 | 24.1 | 25.5 | 22.2 | 23.5 | 22.9 | 22.4 | 21.9 | 15.5 | 24.8 | 15.8 | 21.7 |
| Geometric *Cq* mean | 20.7 | 21.8 | 24.0 | 25.4 | 22.2 | 23.2 | 22.9 | 22.3 | 21.9 | 15.4 | 24.7 | 15.8 | 21.6 |
| Minimum *Cq* | 17.7 | 16.9 | 21.5 | 23.1 | 19.7 | 18.3 | 21.3 | 16.5 | 21.6 | 12.6 | 20.2 | 12.6 | 20.7 |
| Maximum *Cq* | 23.1 | 24.4 | 26.7 | 27.2 | 24.4 | 27.5 | 24.4 | 25.3 | 22.3 | 18.4 | 26.1 | 17.9 | 23.3 |
| *Cq* difference | 5.4 | 7.5 | 5.1 | 4.1 | 4.7 | 9.3 | 3.2 | 8.8 | 0.6 | 5.8 | 5.8 | 5.3 | 2.6 |
| Maximum fold-change | 41.0 | 182.9 | 35.1 | 16.9 | 25.5 | 612.9 | 9.0 | 457.2 | 1.6 | 55.4 | 57.0 | 40.0 | 6.0 |
| *CV* | 1.21 | 2.22 | 0.82 | 0.91 | 1.02 | 1.58 | 0.50 | 2.76 | 0.08 | 1.01 | 2.11 | 1.38 | 0.40 |

*SD*: standard deviation; *CV*: percent coefficient of variation; Size of sample cohort: 32.

*Cq* values were adjusted to 100% efficiency.

In blue font: established plasma miRNA normalizers selected from literature.

Maximum fold-change: 2^(maximum^ *^Cq –^*^sample^ *^Cq^*^)^

Calculation of *CV* values included linear scale transformation (linearisation) of *Cq* values (2^–^*^Cq^*) (Marabita *et al.* 2016; Sundaram *et al.* 2019).

References

Marabita, F., P. de Candia, A. Torri, J. Tegner, S. Abrignani *et al.*, 2016 Normalization of circulating microRNA expression data obtained by quantitative real-time RT-PCR. Briefings in Bioinformatics 17**:** 204-212.

Sundaram, V. K., N. K. Sampathkumar, C. Massaad and J. Grenier, 2019 Optimal use of statistical methods to validate reference gene stability in longitudinal studies. PLoS One 14**:** e0219440.
