# Supplementary material for: Identification of extremely GC-rich micro RNAs for RT-qPCR data normalization in human plasma
Source: Front Genet. 2023 Jan 4;13:1058668. doi: 10.3389/fgene.2022.1058668 (PMC9846067; doi:10.3389/fgene.2022.1058668)
Supplement: Supplementary file 1 [file DataSheet1.zip › Supporting information/Table_S13_Secondary_structure_of_common_spike-in_controls.docx]

**Table S13 |** Predicted secondary structures of spike-in miRNAs for mammalian samples.

| **miRNA spike** | **miRBase ID** | **GC content (%)** | **Sequence,**  **base-pairing probability** | **Ref.** | ***MEF*** |
| --- | --- | --- | --- | --- | --- |
| ath-mir-159a | MIMAT0000177 | 45.0 | uuuggaugaagggagcucua | [1, 2] | 0.0 |
| ath-miR-172a | MIMAT0000203 | 38.1 | agaaucuugaugaugcugcau  ((.(((.....))).)).... | [3, 4] | –1.0 |
| ath-miR-394a | MIMAT0000936 | 55.0 | uuggcauucuguccaccucc  .(((((...)).)))..... | [3] | –1.4 |
| cel-miR-39-3p | MIMAT0000010 | 50.0 | ucaccggguguaaaucagcuug  ....(((((........))))) | [2, 5-8] | –2.2 |
| cel-miR-39-5p | MIMAT0020306 | 36.4 | agcugauuucgucuugguaaua | [9] | –0.4 |
| cel-miR-54-3p | MIMAT0000025 | 41.7 | uacccguaaucuucauaauccgag | [6, 8] | 0.0 |
| cel-miR-238-3p | MIMAT0000293 | 43.5 | uuuguacuccgaugccauucaga | [6, 8, 9] | 0.0 |
| cel-miR-239b-5p | MIMAT0000295 | 31.8 | uuuguacuacacaaaaguacug  ...(((((.......))))).. | [10] | –3.5 |
| osa-miR414 | MIMAT0001330 | 72.5 | ucauccucaucaucaucgucc | [11, 12] | 0.0 |
| osa-miR442 | MIMAT0001605 | 42.7 | ugacguguaaauugcgagacgaau  ...((((.....))))........ | [12] | –1.0 |
| Spike-A | artificial sequence | 63.0 | ugcagcccuaccgacacguucc | [13] | 0.0 |

*MEF*: minimum free energy (kcal/mol) of RNAFold server (<http://rna.tbi.univie.ac.at/cgi-bin/RNAWebSuite/RNAfold.cgi>)

Dot plot depicts probability of base pairing.

cel: worm *Caenorhabditis elegans*; ath: *Arabidopsis thaliana*; osa: *Oryza sativa*

REFERENCES

1. Patuleia SIS, van der Wall E, van Gils CH, Bakker MF, Jager A, Voorhorst-Ogink MM, van Diest PJ, Moelans CB: **The changing microRNA landscape by color and cloudiness: a cautionary tale for nipple aspirate fluid biomarker analysis**. *Cell Oncol (Dordr)* 2021, **44**(6):1339-1349.

2. Marabita F, de Candia P, Torri A, Tegner J, Abrignani S, Rossi RL: **Normalization of circulating microRNA expression data obtained by quantitative real-time RT-PCR**. *Briefings in Bioinformatics* 2016, **17**(2):204-212.

3. Redshaw N, Wilkes T, Whale A, Cowen S, Huggett J, Foy CA: **A comparison of miRNA isolation and RT-qPCR technologies and their effects on quantification accuracy and repeatability**. *Biotechniques* 2013, **54**(3):155-+.

4. Shihana F, Wong WKM, Joglekar MV, Mohamed F, Gawarammana IB, Isbister GK, Hardikar AA, Seth D, Buckley NA: **Urinary microRNAs as non-invasive biomarkers for toxic acute kidney injury in humans**. *Sci Rep-Uk* 2021, **11**(1).

5. McAlexander MA, Phillips MJ, Witwer KW: **Comparison of Methods for miRNA Extraction from Plasma and Quantitative Recovery of RNA from Cerebrospinal Fluid**. *Front Genet* 2013, **4**:83.

6. Burgos KL, Javaherian A, Bomprezzi R, Ghaffari L, Rhodes S, Courtright A, Tembe W, Kim S, Metpally R, Van Keuren-Jensen K: **Identification of extracellular miRNA in human cerebrospinal fluid by next-generation sequencing**. *RNA* 2013, **19**(5):712-722.

7. Cirera S, Andersen-Ranberg EU, Langkilde S, Aaquist M, Gredal H: **Challenges and standardization of microRNA profiling in serum and cerebrospinal fluid in dogs suffering from non-infectious inflammatory CNS disease**. *Acta Vet Scand* 2019, **61**(1):57.

8. Mitchell PS, Parkin RK, Kroh EM, Fritz BR, Wyman SK, Pogosova-Agadjanyan EL, Peterson A, Noteboom J, O'Briant KC, Allen A *et al*: **Circulating microRNAs as stable blood-based markers for cancer detection**. *P Natl Acad Sci USA* 2008, **105**(30):10513-10518.

9. Stein EV, Duewer DL, Farkas N, Romsos EL, Wang LL, Cole KD: **Steps to achieve quantitative measurements of microRNA using two step droplet digital PCR**. *Plos One* 2017, **12**(11).

10. Babion I, Snoek BC, van de Wiel MA, Wilting SM, Steenbergen RDM: **A Strategy to Find Suitable Reference Genes for miRNA Quantitative PCR Analysis and Its Application to Cervical Specimens**. *Journal of Molecular Diagnostics* 2017, **19**(5):625-637.

11. **TECH NOTE: nCounter® miRNA Expression Analysis in Plasma and Serum Samples** [<https://nanostring.com/wp-content/uploads/TN_MK1342_Plasma-Serum_r6.pdf>]

12. Mantel PY, Hjelmqvist D, Walch M, Kharoubi-Hess S, Nilsson S, Ravel D, Ribeiro M, Gruring C, Ma S, Padmanabhan P *et al*: **Infected erythrocyte-derived extracellular vesicles alter vascular function via regulatory Ago2-miRNA complexes in malaria**. *Nat Commun* 2016, **7**:12727.

13. Androvic P, Romanyuk N, Urdzikova-Machova L, Rohlova E, Kubista M, Valihrach L: **Two-tailed RT-qPCR panel for quality control of circulating microRNA studies**. *Sci Rep* 2019, **9**(1):4255.
