# Supplementary material for: Identification of extremely GC-rich micro RNAs for RT-qPCR data normalization in human plasma
Source: Front Genet. 2023 Jan 4;13:1058668. doi: 10.3389/fgene.2022.1058668 (PMC9846067; doi:10.3389/fgene.2022.1058668)
Supplement: Supplementary file 1 [file DataSheet1.zip › Supporting information/Table_S14_Current_repertoire_of_miRNA_references_for_human_plasma.docx]

**Table S14 |** Current normalizers for RT-qPCR-based miRNA expression analysis of plasma/serum from healthy human individuals.

| **Normalizer** | **Identity number** | **Chromo-some** | **%GC**^†^ | **Biofluid** | **Condition** | **Method** | **Stability assessment** | **Cohort size** | **Citation** |
| --- | --- | --- | --- | --- | --- | --- | --- | --- | --- |
| U6 snRNA (*RNU6-1*) | 26827 | 15 | 46.2 | plasma, serum | 2^nd^ trimester of pregnancy | RT-qPCR | not specified | 35 | (1) |
| U6 snRNA,  let-7b-5p | 26827, MIMAT0000063 | 15,  22 | 46.2,  45.4 | plasma, serum | normal condition | RT-qPCR | not specified | 4 | (2) |
| let-7a-5p | MIMAT0000062 | 9, 11, 22 | 36.4 | serum | healthy condition | RT-qPCR | comprehensive stability (geNorm, NormFinder, BestKeeper, Delta *Ct*) | 20 | (3) |
| let-7i-5p, let-7d-5p, let-7g-5p | MIMAT0000415, MIMAT0000065, MIMAT0000414 | 9,  9,  3 | 40.9,  40.9, 36.4 | serum | pathologies & healthy condition | **SBS**,  RT-qPCR | geNorm, NormFinder | 257, 200, 320 & 1278 | (4) |
| miR-16-5p | MIMAT0000069 | 3, 13 | 45.5 | serum | gastric cancer & healthy controls^¶^ | RT-qPCR | comprehensive stability of geNorm, NormFinder, BestKeeper, Delta *Ct* | 40, 20 | (5) |
| miR-16-5p | MIMAT0000069 | 3, 13 | 45.5 | serum | non-ischaemic heart failure & healthy controls | RT-qPCR | see (5) | 14, 8 | (6) |
| miR-21a-5p | MIMAT0000076 | 17 | 36.4 | plasma | physical training | RT-qPCR  (179 miRNAs) | NormFinder | 24 | (7) |
| miR-25-3p | MIMAT0000081 | 7 | 50.0 | serum | colorectal cancer & healthy controls | qPCR-based assay | geNorm, NormFinder, BestKeeper | 30, 30 | (8) |
| miR-30e-5p (with miR-148b-3p) | MIMAT0000692 | 1 | 40.9 | plasma | endometriosis & control women | RT-qPCR | similar abundance across groups | 53, 53 | (9) |
| miR-92a-3p | MIMAT0000092 | 13 | 54.5 | plasma | hypertension & healthy controls^¶^ | RT-qPCR | geNorm, NormFinder | 18, 10 | (10) |
| miR-93-5p*^,\|\|^ | MIMAT0000093 | 7 | 52.2 | serum | healthy condition | RT-qPCR | comprehensive stability of geNorm, NormFinder, BestKeeper, Delta *Ct* | 20 | (3) |
| miR-93 | MIMAT0000093 | 7 | 52.2 | plasma | healthy condition and training mode | RT-qPCR | NormFinder | 30 | (11) |
| miR-103a-3p* | MIMAT0000101 | 5, 20 | 47.8 | serum | psychological activity (healthy controls and ischemic heart disease) | RT-qPCR | not specified | 90 patients, 30 controls | (12) |
| miR-126-3p^\|\|^ | MIMAT0000445 | 9 | 45.5 | plasma | healthy condition & centenarians | RT-qPCR | NormFinder | 20 | (13) |
| let‑7i‑3p,  miR‑148‑3p^§^ | MIMAT0004585,MIMAT0000243 | 12,  7 | 59.1,  40.9 | plasma, serum | low‑risk pregnancy | RT-qPCR | not specified | 10 | (14) |
| miR-185 | MIMAT0000455 | 22 | 50.0 | plasma | normal | RT-qPCR (TaqMan card) | coefficient of variation | 6 (3 ♂, 3 ♀) | (15) |
| miR-188-5p,  miR-222-3p^‡^ | MIMAT0000457, MIMAT0000279 | X,  X | 61.9,  52.4 | plasma | circulating oestradiol | NanoString's nCounter miRNA assay | NormFinder, BestKeeper | 20 | (16) |
| miR-191* | MIMAT0000440 | 3 | 52.2 | serum | urban, community-dwelling young and old individuals (Whites and African Americans) | RT-qPCR | least variable expression, not associated with age | 11 (6♀, 5♂; age: ~30), 11 (5 ♀, 6♂; age: ~65) | (17) |
| miR-222-3p | MIMAT0000279 | X | 52.4 | plasma | pulmonary arterial hypertension & healthy controls | RT-qPCR | NormFinder | 27 (14, 13) | (18) |
| miR-223 | MIMAT0004570 | X | 40.9 | plasma, serum | not specified | RT-qPCR | not specified | not specified | (19) |
| miR-320d | MIMAT0006764 | 13, X | 47.4 | plasma | physical training | RT-qPCR  (179 miRNAs) | NormFinder, significance of target-miRNA expression | 24 | (7) |
| miR-328-3p | MIMAT0000752 | 16 | 68.2 | plasma | diabetic retinopathy & healthy controls | RT-qPCR | NormFinder, RefFinder | 62, 60^#^ | (20) |
| miR-345 | MIMAT0000772 | 14 | 63.6 | serum | normal | RT-qPCR | coefficient of variation | 6 (3 ♂, 3 ♀) | (15) |
| miR-425-5p^#^, miR-484 | MIMAT000339, MIMAT0002174 | 3,  16 | 47.8,  63.6 | plasma | sprints training | not specified | not specified | 18 | (21) |
| miR-520d-5p | MIMAT0002855 | 19 | 50.0 | plasma | healthy controls | RT-qPCR | narrow standard deviation | 12 | (22) |
| miR-3665 | MIMAT0018087 | 13 | 83.3 | serum | malignant pleural mesothelioma & healthy controls | **microarray**, RT-qPCR | CV, ANOVA, NormFinder | 20, 14 | (23) |
| miR-6090,  miR-4516 | MIMAT0023715, MIMAT0019053 | 11,  16 | 89.5,  76.5 | plasma | coronary artery disease & healthy controls | **microarray**, RT-qPCR | CV, NormFinder, BestKeeper | 8 & 8; 21 &21 | (24) |

Abbreviations: analysis of variance (ANOVA), coefficient of variation (CV), standard deviation (SD), small nuclear RNA (snRNA)

Identity numbers: Gene ID at NCBI or accession number of mature miRNA at miRBase ([www.mirbase.org](http://www.mirbase.org))

Clustered miRNAs are depicted by identical colour (for example, miR-191 and miR-425)

Genome location was derived from RNAcentral (<https://rnacentral.org>)

Underlined: miRNAs that Qiagen has identified as stably expressed in human serum/plasma (25)

*a typically detected but not necessarily stably expressed miRNA (25)

^#^Comprehensive ranking tool based on the stability algorithms geNorm, BestKeeper, NormFinder and Delta Ct ([www.heartcure.com.au/reffinder/](http://www.heartcure.com.au/reffinder/))

^†^GC content (in percent) determined at [www.endmemo.com/bio/gc.php](http://www.endmemo.com/bio/gc.php)

^§^Normalization: geometric mean of hsa‑let‑7i‑3p and hsa‑miR‑148‑3p

^‡^Normalization: *Cq* average of the two miRNAs

^¶^No differences were found between miRNAs of the patient group and healthy controls.

^||^associated with age and body mass index (26)

In bold: undirected profiling to select miRNA reference genes using microarray expression analysis or sequencing by synthesis (SBS) instead of selecting traditionally utilized miRNA references from peer-reviewed literature

REFERENCES

1. Ge, Q., Shen, Y., Tian, F., Lu, J., Bai, Y., and Lu, Z. (2015) Profiling circulating microRNAs in maternal serum and plasma. *Molecular Medicine Reports* **12**, 3323-3330

2. Dufourd, T., Robil, N., Mallet, D., Carcenac, C., Boulet, S., Brishoual, S., Rabois, E., Houeto, J. L., De La Grange, P., and Carnicella, S. (2019) Plasma or serum? A qualitative study on rodents and humans using high-throughput microRNA sequencing for circulating biomarkers. *Biology Methods and Protocols* **4**, bpz006

3. Song, J. N., Bai, Z. G., Han, W., Zhang, J., Meng, H., Bi, J. T., Ma, X. M., Han, S. W., and Zhang, Z. T. (2012) Identification of Suitable Reference Genes for qPCR Analysis of Serum microRNA in Gastric Cancer Patients. *Digest Dis Sci* **57**, 897-904

4. Chen, X., Liang, H., Guan, D., Wang, C., Hu, X., Cui, L., Chen, S., Zhang, C., Zhang, J., Zen, K., and Zhang, C.-Y. (2013) A combination of Let-7d, Let-7g and Let-7i serves as a stable reference for normalization of serum microRNAs. *PloS one* **8**, e79652-e79652

5. Song, J., Bai, Z., Han, W., Zhang, J., Meng, H., Bi, J., Ma, X., Han, S., and Zhang, Z. (2012) Identification of suitable reference genes for qPCR analysis of serum microRNA in gastric cancer patients. *Dig Dis Sci* **57**, 897-904

6. Vogel, B., Keller, A., Frese, K. S., Leidinger, P., Sedaghat-Hamedani, F., Kayvanpour, E., Kloos, W., Backe, C., Thanaraj, A., Brefort, T., Beier, M., Hardt, S., Meese, E., Katus, H. A., and Meder, B. (2013) Multivariate miRNA signatures as biomarkers for non-ischaemic systolic heart failure. *European Heart Journal* **34**, 2812-+

7. Faraldi, M., Gomarasca, M., Sansoni, V., Perego, S., Banfi, G., and Lombardi, G. (2019) Normalization strategies differently affect circulating miRNA profile associated with the training status. *Sci Rep* **9**, 1584

8. Niu, Y., Wu, Y., Huang, J., Li, Q., Kang, K., Qu, J., Li, F., and Gou, D. (2016) Identification of reference genes for circulating microRNA analysis in colorectal cancer. *Sci Rep* **6**, 35611

9. Papari, E., Noruzinia, M., Kashani, L., and Foster, W. G. (2020) Identification of candidate microRNA markers of endometriosis with the use of next-generation sequencing and quantitative real-time polymerase chain reaction. *Fertil Steril* **113**, 1232-1241

10. Solayman, M. H., Langaee, T., Patel, A., El-Wakeel, L., El-Hamamsy, M., Badary, O., and Johnson, J. A. (2016) Identification of Suitable Endogenous Normalizers for qRT-PCR Analysis of Plasma microRNA Expression in Essential Hypertension. *Mol Biotechnol* **58**, 179-187

11. Wardle, S. L., Bailey, M. E. S., Kilikevicius, A., Malkova, D., Wilson, R. H., Venckunas, T., and Moran, C. N. (2015) Plasma MicroRNA Levels Differ between Endurance and Strength Athletes. *Plos One* **10**

12. Dal Lin, C., Marinova, M., Brugnolo, L., Rubino, G., Plebani, M., Iliceto, S., and Tona, F. (2021) Rapid changes of miRNAs-20, -30, -410, -515, -134, and -183 and telomerase with psychological activity: A one year study on the relaxation response and epistemological considerations. *J Tradit Complement Med* **11**, 409-418

13. Balzano, F., Deiana, M., Dei Giudici, S., Oggiano, A., Pasella, S., Pinna, S., Mannu, A., Deiana, N., Porcu, B., Masala, A. G. E., Pileri, P. V., Scognamillo, F., Pala, C., Zinellu, A., Carru, C., and Deiana, L. (2017) MicroRNA Expression Analysis of Centenarians and Rheumatoid Arthritis Patients Reveals a Common Expression Pattern. *International Journal of Medical Sciences* **14**, 622-628

14. Parker, V. L., Gavriil, E., Marshall, B., Pacey, A., and Heath, P. R. (2021) Profiling microRNAs in uncomplicated pregnancies: Serum vs. plasma. *Biomed Rep* **14**, 24

15. Wang, K., Yuan, Y., Cho, J. H., McClarty, S., Baxter, D., and Galas, D. J. (2012) Comparing the MicroRNA Spectrum between Serum and Plasma. *Plos One* **7**, e41561

16. Tay, J. W., James, I., Hughes, Q. W., Tiao, J. Y., and Baker, R. I. (2017) Identification of reference miRNAs in plasma useful for the study of oestrogen-responsive miRNAs associated with acquired Protein S deficiency in pregnancy. *BMC Res Notes* **10**, 312

17. Noren Hooten, N., Fitzpatrick, M., Wood, W. H., 3rd, De, S., Ejiogu, N., Zhang, Y., Mattison, J. A., Becker, K. G., Zonderman, A. B., and Evans, M. K. (2013) Age-related changes in microRNA levels in serum. *Aging (Albany NY)* **5**, 725-740

18. Schlosser, K., McIntyre, L. A., White, R. J., and Stewart, D. J. (2015) Customized Internal Reference Controls for Improved Assessment of Circulating MicroRNAs in Disease. *PLoS One* **10**, e0127443

19. Kroh, E. M., Parkin, R. K., Mitchell, P. S., and Tewari, M. (2010) Analysis of circulating microRNA biomarkers in plasma and serum using quantitative reverse transcription-PCR (qRT-PCR). *Methods (San Diego, Calif.)* **50**, 298-301

20. Prado, M. S. G., de Goes, T. C., de Jesus, M. L., Mendonca, L. S. O., Nascimento, J. S., and Kaneto, C. M. (2019) Identification of miR-328-3p as an endogenous reference gene for the normalization of miRNA expression data from patients with Diabetic Retinopathy. *Sci Rep-Uk* **9**

21. Sansoni, V., Perego, S., Vernillo, G., Barbuti, A., Merati, G., La Torre, A., Banfi, G., and Lombardi, G. (2018) Effects of repeated sprints training on fracture risk-associated miRNA. *Oncotarget* **9**, 18029-18040

22. Rice, J., Roberts, H., Rai, S. N., and Galandiuk, S. (2015) Housekeeping genes for studies of plasma microRNA: A need for more precise standardization. *Surgery* **158**, 1345-1351

23. Bononi, I., Comar, M., Puozzo, A., Stendardo, M., Boschetto, P., Orecchia, S., Libener, R., Guaschino, R., Pietrobon, S., Ferracin, M., Negrini, M., Martini, F., Bovenzi, M., and Tognon, M. (2016) Circulating microRNAs found dysregulated in ex-exposed asbestos workers and pleural mesothelioma patients as potential new biomarkers. *Oncotarget* **7**, 82700-82711

24. Zhang, Y. J., Tang, W. X., Peng, L., Tang, J. Q., and Yuan, Z. K. (2016) Identification and validation of microRNAs as endogenous controls for quantitative polymerase chain reaction in plasma for stable coronary artery disease. *Cardiology Journal* **23**, 694-703

25. Qiagen. (2019) Guidelines for Profiling Biofluid miRNAs. Qiagen

26. Ameling, S., Kacprowski, T., Chilukoti, R. K., Malsch, C., Liebscher, V., Suhre, K., Pietzner, M., Friedrich, N., Homuth, G., Hammer, E., and Volker, U. (2015) Associations of circulating plasma microRNAs with age, body mass index and sex in a population-based study. *BMC Med Genomics* **8**, 61
