# Supplementary material for: Identification of extremely GC-rich micro RNAs for RT-qPCR data normalization in human plasma
Source: Front Genet. 2023 Jan 4;13:1058668. doi: 10.3389/fgene.2022.1058668 (PMC9846067; doi:10.3389/fgene.2022.1058668)
Supplement: Supplementary file 1 [file DataSheet1.zip › Supporting information/Table_S17_An_extended_set_of_eight_miRNA_reference_genes_for_RT-qPCR_normalisation_of_human_plasma_samples..DOCX]

**Table S17 |** An extended set of eight miRNA reference genes for RT-qPCR normalisation of human plasma samples.

| **No.** | **Mature miRNA** | ***CV*** | **exomiR (plasma/ serum)*** | **Differential expression in plasma/serum** | **Functions** |
| --- | --- | --- | --- | --- | --- |
| 1. | miR-3665 | 0.08 | yes (serum (Lim *et al.* 2020)) | down:   - familial ALS (plasma (Freischmidt *et al.* 2014))   up:   - HAND (plasma (Asahchop *et al.* 2016)) - TBI (plasma (Qin *et al.* 2018)) | - tumour suppressor (Braoudaki et al. 2014); reduced viability and/or anchorage independent growth of cancer cells (Wilting *et al.* 2016) - development of neurological functions (Asahchop *et al.* 2016) |
| 2. | miR-1915-3p | 0.50 | yes (serum (Lim *et al.* 2020)) | down:   - familial ALS (plasma (Freischmidt *et al.* 2014))   up:   - BCa (serum (Guo *et al.* 2018b)) - PCa (sEVs (Ali *et al.* 2021)) - remission of RA compared to non-clinical remission group (serum exosomes (Lim *et al.* 2020) ) | - DNA damage response (Nakazawa *et al.* 2014) - TP53-dependent, oxidative-stress responsive in HCC (Wan *et al.* 2017) - two-faced Janus of cancer: tumour suppressor (Cui *et al.* 2019) and inhibitor of cell migration (Pan *et al.* 2021), but inhibits apoptosis of LCa cells (Xu *et al.* 2016) and enhances cell proliferation and migration of BCa cells *in vitro* (Guo *et al.* 2018a) |
| 3. | miR-185-5p | 0.82 | yes (plasma (Zhang *et al.* 2019a; Wang *et al.* 2022); serum (Zhang *et al.* 2020a)) | down:   - OCa (plasma exosomes (Zhang et al. 2019a)) - NSCLC (serum exosomes (Zhang *et al.* 2020a)) - AD (plasma exosomes (Wang *et al.* 2022))   up:   - smoking (plasma (Takahashi *et al.* 2013)) | - induces cell-cycle arrest in human NSCLC (Takahashi *et al.* 2009) - induce telomere dysfunction and cellular senescence (Li *et al.* 2020a) - regulates development of myocardial fibrosis (Lin *et al.* 2022) - repression of cells growth and metastasis of osteosarcoma (Wu *et al.* 2022b) - predominantly exhibits tumor-suppressive function (Babaeenezhad *et al.* 2022) |
| 4. | miR-320d | 0.91 | yes (serum (Yagi *et al.* 2017; Tang *et al.* 2019; Li *et al.* 2020b)) | down:   - adenoma and CRC (plasma (Liu *et al.* 2019)) - HCC (serum exosomes (Li *et al.* 2020b));   up:   - AML (serum (Zhi *et al.* 2013)) - metastatic CRC (serum exosomes (Tang *et al.* 2019)) | - promotes apoptosis of PCa cells (Lin *et al.* 2017) - regulates tumour growth and invasion of GCA (Chen *et al.* 2020) - suppresses the progression of BCa (Shi *et al.* 2018), EGFR-positive CRC (Zhu *et al.* 2021) and EMT (Sugano *et al.* 2021) - deregulated in diabetes, acute stroke, and infant RSV infection (references in (Li *et al.* 2020b)) |
| 5. | miR-3960-5p | 1.01 | yes (serum (Kim *et al.* 2020)) | T2D (serum (Ding *et al.* 2016))  down:  BlCa (serum (Usuba *et al.* 2019)) | - osteoblast differentiation (Hu *et al.* 2011) - nervous system development (miRPathDB 2.0) - Wnt signaling pathway (Salehi and Akrami 2017) |
| 6. | miR-425-5p | 1.02 | yes (serum (Taylor and Gercel-Taylor 2008; Yagi *et al.* 2017; Zhang *et al.* 2019b; Zhang *et al.* 2020b)) | down:   - MM (serum exosomes (Zhang *et al.* 2019b)) - NSCLC (serum exosomes (Zhang *et al.* 2020b))   up:  smoking (plasma (Takahashi *et al.* 2013)) | - oncogenic in GA (Yan *et al.* 2017b), BCa (Xiao et al. 2019), HCC (Wu *et al.* 2019; Rao *et al.* 2020; Wu *et al.* 2022a), CRC (Liu *et al.* 2020), OCa (Wu *et al.* 2021b) and DLBC (Wu *et al.* 2021a) - plasma biomarker of cardiovascular disease in RA patients (Taverner et al. 2021) - modulates osteoporosis (Chen *et al.* 2021) |
| 7. | miR-93-5p | 1.21 | yes (plasma (Zhang *et al.* 2019a; Jafari *et al.* 2022)); (serum exosomes (Zhang *et al.* 2017)) | up:   - OCa (serum (Resnick *et al.* 2009)) - ESCC (plasma (Liao *et al.* 2016; Liu *et al.* 2018)) - male LSCC patients (serum and serum exosomes (Zhang *et al.* 2017)) - OCa (plasma exosomes (Zhang *et al.* 2019a)) - T2D (plasma exosomes (Jafari *et al.* 2022)) | - oncogenic activity and opposing role in other tumour types (Yang *et al.* 2022) |
| 8. | miR-4787-5p | 1.38 | yes (serum (Yan *et al.* 2017a)) | down:   - familial ALS (plasma (Freischmidt *et al.* 2014)) - CRC (serum exosomes (Yan *et al.* 2017a)) (Cojocneanu *et al.* 2020)   up:   - ADD (plasma (Wang *et al.* 2017)) | - growth control and EMT resistance in PDAC (Mody *et al.* 2016) - regulates apoptosis of vascular smooth muscle cells (Wang *et al.* 2021) |

*According to Vesiclepedia ([www.microvesicles.org](http://www.microvesicles.org)) and additional reports

^#^According to The Extracellular Vesicles miRNA Database (EVmiRNA; <http://bioinfo.life.hust.edu.cn/EVmiRNA/#!/>)

exomiR: exosomal miRNA; *CV*: coefficient of variation;

Physiological conditions are abbreviated as follows. AAD: acute aortic dissection; ALS: amyotrophic lateral sclerosis; AML: acute myeloid leukaemia; BCa: breast cancer; BlCa: bladder cancer; CRC: colorectal cancer; DLBC: diffuse large B-cell lymphoma; EMT: epithelial–mesenchymal transition; ESCC: esophageal squamous cell carcinoma; GA: gastric cancer; GCA: gastric cardiac adenocarcinoma; HAND: HIV-associated neurocognitive disorder; HCC: hepatocellular carcinoma; LSCC: laryngeal squamous cell carcinoma; LCa: lung cancer; LSCC: lung squamous cell carcinoma; MM: multiple myeloma; NSCLC: non-small lung cancer; OCa: ovarian cancer; PCa: prostate cancer; PDAC: pancreatic ductal adenocarcinoma; RA: rheumatoid arthritis; RSV: respiratory syncytial virus; sEVs: small extracellular vesicles; SM: severe malaria; TBI: traumatic brain injury; T2D: type 2 diabetes.

REFERENCES

Ali, H. E. A., M. S. A. Gaballah, R. Gaballa, S. Mahgoub, Z. A. Hassan *et al.*, 2021 Small Extracellular Vesicle-Derived microRNAs Stratify Prostate Cancer Patients According to Gleason Score, Race and Associate with Survival of African American and Caucasian Men. Cancers (Basel) 13.

Asahchop, E. L., S. M. Akinwumi, W. G. Branton, E. Fujiwara, M. J. Gill *et al.*, 2016 Plasma microRNA profiling predicts HIV-associated neurocognitive disorder. AIDS 30**:** 2021-2031.

Babaeenezhad, E., F. Naghibalhossaini, M. Rajabibazl, Z. Jangravi, F. Hadipour Moradi *et al.*, 2022 The Roles of microRNA miR-185 in Digestive Tract Cancers. Noncoding RNA 8.

Braoudaki, M., G. I. Lambrou, K. Giannikou, V. Milionis, K. Stefanaki *et al.*, 2014 Microrna expression signatures predict patient progression and disease outcome in pediatric embryonal central nervous system neoplasms. J Hematol Oncol 7**:** 96.

Chen, G., G. Huang, H. Lin, X. Wu, X. Tan *et al.*, 2021 MicroRNA-425-5p modulates osteoporosis by targeting annexin A2. Immun Ageing 18**:** 45.

Chen, X., S. Gao, Z. Zhao, G. Liang, J. Kong *et al.*, 2020 MicroRNA-320d regulates tumor growth and invasion by promoting FoxM1 and predicts poor outcome in gastric cardiac adenocarcinoma. Cell Biosci 10**:** 80.

Cojocneanu, R., C. Braicu, L. Raduly, A. Jurj, O. Zanoaga *et al.*, 2020 Plasma and Tissue Specific miRNA Expression Pattern and Functional Analysis Associated to Colorectal Cancer Patients. Cancers (Basel) 12.

Cui, H. W., W. Y. Han, L. N. Hou, L. Yang, X. Li *et al.*, 2019 miR-1915-3p inhibits Bcl-2 expression in the development of gastric cancer. Biosci Rep 39.

Ding, L., D. Ai, R. Wu, T. Zhang, L. Jing *et al.*, 2016 Identification of the differential expression of serum microRNA in type 2 diabetes. Biosci Biotechnol Biochem 80**:** 461-465.

Freischmidt, A., K. Muller, L. Zondler, P. Weydt, A. E. Volk *et al.*, 2014 Serum microRNAs in patients with genetic amyotrophic lateral sclerosis and pre-manifest mutation carriers. Brain 137**:** 2938-2950.

Guo, J., C. Liu, W. Wang, Y. Liu, H. He *et al.*, 2018a Identification of serum miR-1915-3p and miR-455-3p as biomarkers for breast cancer. PLoS One 13**:** e0200716.

Guo, J., C. Liu, W. Wang, Y. Liu, H. W. He *et al.*, 2018b Identification of serum miR-1915-3p and miR455-3p as biomarkers for breast cancer. Plos One 13.

Hu, R., W. Liu, H. Li, L. Yang, C. Chen *et al.*, 2011 A Runx2/miR-3960/miR-2861 regulatory feedback loop during mouse osteoblast differentiation. J Biol Chem 286**:** 12328-12339.

Jafari, N., A. Chen, M. Kolla, I. R. Pompa, Y. Qiu *et al.*, 2022 Novel plasma exosome biomarkers for prostate cancer progression in co-morbid metabolic disease. Adv Cancer Biol Metastasis 6.

Kim, H., Y. U. Bae, H. Lee, H. Kim, J. S. Jeon *et al.*, 2020 Effect of diabetes on exosomal miRNA profile in patients with obesity. BMJ Open Diabetes Res Care 8.

Li, T., Z. Luo, S. Lin, C. Li, S. Dai *et al.*, 2020a MiR-185 targets POT1 to induce telomere dysfunction and cellular senescence. Aging (Albany NY) 12**:** 14791-14807.

Li, W. X., X. M. Ding, S. H. Wang, L. Xu, T. Yin *et al.*, 2020b Downregulation of serum exosomal miR-320d predicts poor prognosis in hepatocellular carcinoma. Journal of Clinical Laboratory Analysis 34.

Liao, J., R. Liu, Y. J. Shi, L. H. Yin and Y. P. Pu, 2016 Exosome-shuttling microRNA-21 promotes cell migration and invasion-targeting PDCD4 in esophageal cancer. Int J Oncol 48**:** 2567-2579.

Lim, M. K., J. Yoo, D. H. Sheen, C. Ihm, S. K. Lee *et al.*, 2020 Serum Exosomal miRNA-1915-3p Is Correlated With Disease Activity of Korean Rheumatoid Arthritis. In Vivo 34**:** 2941-2945.

Lin, C.-L., C.-M. Chen, C.-L. Lin, C.-W. Cheng, C.-H. Lee *et al.*, 2017 Norcantharidin induces mitochondrial-dependent apoptosis through Mcl-1 inhibition in human prostate cancer cells. Biochimica et Biophysica Acta (BBA) - Molecular Cell Research 1864**:** 1867-1876.

Lin, R., L. Rahtu-Korpela, Z. Szabo, A. Kemppi, S. Skarp *et al.*, 2022 MiR-185-5p regulates the development of myocardial fibrosis. J Mol Cell Cardiol 165**:** 130-140.

Liu, D., H. Zhang, M. Cui, C. Chen and Y. Feng, 2020 Hsa-miR-425-5p promotes tumor growth and metastasis by activating the CTNND1-mediated beta-catenin pathway and EMT in colorectal cancer. Cell Cycle 19**:** 1917-1927.

Liu, M. X., J. Liao, M. Xie, Z. K. Gao, X. H. Wang *et al.*, 2018 miR-93-5p Transferred by Exosomes Promotes the Proliferation of Esophageal Cancer Cells via Intercellular Communication by Targeting PTEN. Biomed Environ Sci 31**:** 171-185.

Liu, X. X., X. N. Xu, B. Pan, B. S. He, X. X. Chen *et al.*, 2019 Circulating miR-1290 and miR-320d as Novel Diagnostic Biomarkers of Human Colorectal Cancer. Journal of Cancer 10**:** 43-50.

Mody, H. R., S. W. Hung, M. AlSaggar, J. Griffin and R. Govindarajan, 2016 Inhibition of S-Adenosylmethionine-Dependent Methyltransferase Attenuates TGFbeta1-Induced EMT and Metastasis in Pancreatic Cancer: Putative Roles of miR-663a and miR-4787-5p. Mol Cancer Res 14**:** 1124-1135.

Nakazawa, K., N. Dashzeveg and K. Yoshida, 2014 Tumor suppressor p53 induces miR-1915 processing to inhibit Bcl-2 in the apoptotic response to DNA damage. Febs Journal 281**:** 2937-2944.

Pan, H. L., Z. H. Pan, F. J. Guo, F. R. Meng, L. L. Zu *et al.*, 2021 MicroRNA-1915-3p inhibits cell migration and invasion by targeting SET in non-small-cell lung cancer. Bmc Cancer 21.

Qin, X. J., L. Z. Li, Q. Lv, Q. M. Shu, Y. L. Zhang *et al.*, 2018 Expression profile of plasma microRNAs and their roles in diagnosis of mild to severe traumatic brain injury. Plos One 13.

Rao, D., S. M. Guan, J. W. Huang, Q. Chang and S. G. Duan, 2020 miR-425-5p Acts as a Molecular Marker and Promoted Proliferation, Migration by Targeting RNF11 in Hepatocellular Carcinoma. Biomed Research International 2020.

Resnick, K. E., H. Alder, J. P. Hagan, D. L. Richardson, C. M. Croce *et al.*, 2009 The detection of differentially expressed microRNAs from the serum of ovarian cancer patients using a novel real-time PCR platform. Gynecol Oncol 112**:** 55-59.

Salehi, Z., and H. Akrami, 2017 Target genes prediction and functional analysis of microRNAs differentially expressed in gastric cancer stem cells MKN-45. J Cancer Res Ther 13**:** 477-483.

Shi, S., X. L. Hu, J. P. Xu, H. Liu and L. B. Zou, 2018 MiR-320d suppresses the progression of breast cancer via lncRNA HNF1A-AS1 regulation and SOX4 inhibition. Rsc Advances 8**:** 19196-19207.

Sugano, T., M. Masuda, F. Takeshita, N. Motoi, T. Hirozane *et al.*, 2021 Pharmacological blockage of transforming growth factor-beta signalling by a Traf2- and Nck-interacting kinase inhibitor, NCB-0846. Br J Cancer 124**:** 228-236.

Takahashi, K., S. Yokota, N. Tatsumi, T. Fukami, T. Yokoi *et al.*, 2013 Cigarette smoking substantially alters plasma microRNA profiles in healthy subjects. Toxicol Appl Pharmacol 272**:** 154-160.

Takahashi, Y., A. R. R. Forrest, E. Maeno, T. Hashimoto, C. O. Daub *et al.*, 2009 MiR-107 and MiR-185 Can Induce Cell Cycle Arrest in Human Non Small Cell Lung Cancer Cell Lines. Plos One 4.

Tang, Y. Y., Y. J. Zhao, X. G. Song, X. R. Song, L. M. Niu *et al.*, 2019 Tumor-derived exosomal miRNA-320d as a biomarker for metastatic colorectal cancer. Journal of Clinical Laboratory Analysis 33.

Taverner, D., D. Llop, R. Rosales, R. Ferre, L. Masana *et al.*, 2021 Plasma expression of microRNA-425-5p and microRNA-451a as biomarkers of cardiovascular disease in rheumatoid arthritis patients. Scientific Reports 11.

Taylor, D. D., and C. Gercel-Taylor, 2008 MicroRNA signatures of tumor-derived exosomes as diagnostic biomarkers of ovarian cancer. Gynecologic Oncology 110**:** 13-21.

Usuba, W., F. Urabe, Y. Yamamoto, J. Matsuzaki, H. Sasaki *et al.*, 2019 Circulating miRNA panels for specific and early detection in bladder cancer. Cancer Sci 110**:** 408-419.

Wan, Y., R. Cui, J. Gu, X. Zhang, X. Xiang *et al.*, 2017 Identification of Four Oxidative Stress-Responsive MicroRNAs, miR-34a-5p, miR-1915-3p, miR-638, and miR-150-3p, in Hepatocellular Carcinoma. Oxid Med Cell Longev 2017**:** 5189138.

Wang, L., Z. Wang, R. Zhang, L. Huang, Z. Zhao *et al.*, 2021 MiR-4787-5p Regulates Vascular Smooth Muscle Cell Apoptosis by Targeting PKD1 and Inhibiting the PI3K/Akt/FKHR Pathway. J Cardiovasc Pharmacol 78**:** 288-296.

Wang, L., S. J. Zhang, Z. G. Xu, J. J. Zhang, L. Li *et al.*, 2017 The diagnostic value of microRNA-4787-5p and microRNA-4306 in patients with acute aortic dissection. American Journal of Translational Research 9**:** 5138-+.

Wang, L., H. Zhen, Y. Sun, S. Rong, B. Li *et al.*, 2022 Plasma Exo-miRNAs Correlated with AD-Related Factors of Chinese Individuals Involved in Abeta Accumulation and Cognition Decline. Mol Neurobiol 59**:** 6790-6804.

Wilting, S. M., V. Miok, A. Jaspers, D. Boon, H. Sorgard *et al.*, 2016 Aberrant methylation-mediated silencing of microRNAs contributes to HPV-induced anchorage independence. Oncotarget 7**:** 43805-43819.

Wu, H., J. Shang, W. Zhan, J. Liu, H. Ning *et al.*, 2019 miR4255p promotes cell proliferation, migration and invasion by directly targeting FOXD3 in hepatocellular carcinoma cells. Mol Med Rep 20**:** 1883-1892.

Wu, S., S. Liu, Y. Cao, G. Chao, P. Wang *et al.*, 2022a Downregulation of ZC3H13 by miR-362-3p/miR-425-5p is associated with a poor prognosis and adverse outcomes in hepatocellular carcinoma. Aging (Albany NY) 14**:** 2304-2319.

Wu, W., L. Chen, C. Chen, L. Yu and J. Zheng, 2021a miRNA-425-5p enhances diffuse large B cell lymphoma growth by targeting PTEN. Transl Cancer Res 10**:** 4905-4913.

Wu, Y., W. Zhou, Z. Yang, J. Li and Y. Jin, 2022b miR-185-5p Represses Cells Growth and Metastasis of Osteosarcoma via Targeting Cathepsin E. Int J Toxicol 41**:** 115-125.

Wu, Z., J. Guo, Y. Zhang, J. Liu, H. Ma *et al.*, 2021b MiR-425-5p accelerated the proliferation, migration, and invasion of ovarian cancer cells via targeting AFF4. J Ovarian Res 14**:** 138.

Xiao, S., H. J. Zhu, J. Luo, Z. R. Wu and M. J. Xie, 2019 miR-425-5p is associated with poor prognosis in patients with breast cancer and promotes cancer cell progression by targeting PTEN. Oncology Reports 42**:** 2550-2560.

Xu, C. S., H. H. Li, L. Zhang, T. J. Jia, L. N. Duan *et al.*, 2016 MicroRNA-1915-3p prevents the apoptosis of lung cancer cells by downregulating DRG2 and PBX2. Molecular Medicine Reports 13**:** 505-512.

Yagi, Y., T. Ohkubo, H. Kawaji, A. Machida, H. Miyata *et al.*, 2017 Next-generation sequencing-based small RNA profiling of cerebrospinal fluid exosomes. Neuroscience Letters 636**:** 48-57.

Yan, S. S., B. Han, S. Y. Gao, X. C. Wang, Z. F. Wang *et al.*, 2017a Exosome-encapsulated microRNAs as circulating biomarkers for colorectal cancer. Oncotarget 8**:** 60149-60158.

Yan, Y. F., F. M. Gong, B. S. Wang and W. Zheng, 2017b MiR-425-5p promotes tumor progression via modulation of CYLD in gastric cancer. Eur Rev Med Pharmacol Sci 21**:** 2130-2136.

Yang, M., R. Xiao, X. Wang, Y. Xiong, Z. Duan *et al.*, 2022 MiR-93-5p regulates tumorigenesis and tumor immunity by targeting PD-L1/CCND1 in breast cancer. Ann Transl Med 10**:** 203.

Zhang, H., S. Xu and X. Liu, 2019a MicroRNA profiling of plasma exosomes from patients with ovarian cancer using high-throughput sequencing. Oncol Lett 17**:** 5601-5607.

Zhang, L., X. Shan, J. Wang, J. Zhu, Z. Huang *et al.*, 2017 A three-microRNA signature for lung squamous cell carcinoma diagnosis in Chinese male patients. Oncotarget 8**:** 86897-86907.

Zhang, Z.-J., X.-G. Song, L. Xie, K.-Y. Wang, Y.-Y. Tang *et al.*, 2020a Circulating serum exosomal miR-20b-5p and miR-3187-5p as efficient diagnostic biomarkers for early-stage non-small cell lung cancer. Experimental Biology and Medicine 245**:** 1428-1436.

Zhang, Z. J., X. G. Song, L. Xie, K. Y. Wang, Y. Y. Tang *et al.*, 2020b Circulating serum exosomal miR-20b-5p and miR-3187-5p as efficient diagnostic biomarkers for early-stage non-small cell lung cancer. Experimental Biology and Medicine 245**:** 1428-1436.

Zhang, Z. Y., Y. C. Li, C. Y. Geng, H. J. Wang and W. M. Chen, 2019b Potential Relationship between Clinical Significance and Serum Exosomal miRNAs in Patients with Multiple Myeloma. Biomed Research International 2019.

Zhi, F., X. S. Cao, X. B. Xie, B. Wang, W. M. Dong *et al.*, 2013 Identification of Circulating MicroRNAs as Potential Biomarkers for Detecting Acute Myeloid Leukemia. Plos One 8.

Zhu, Y. F., M. Qi and D. D. Wu, 2021 MiR-320d Inhibits Progression of EGFR-Positive Colorectal Cancer by Targeting TUSC3. Frontiers in Genetics 12.
