# Supplementary material for: Identification of extremely GC-rich micro RNAs for RT-qPCR data normalization in human plasma
Source: Front Genet. 2023 Jan 4;13:1058668. doi: 10.3389/fgene.2022.1058668 (PMC9846067; doi:10.3389/fgene.2022.1058668)
Supplement: Supplementary file 1 [file DataSheet1.zip › Supporting information/Table_S19_Underrepresentation_of_GC-extreme_miRNAs_by_sRNA-seq.docx]

**Table S19 |**Underrepresentation of extremely GC-rich miRNAs by sRNA-seq.

| **Profiling technique** | **Human material** | **Sample number** | **Detection of GC-extreme miRNA** | | | | | | **Refe-rence** |
| --- | --- | --- | --- | --- | --- | --- | --- | --- | --- |
|  |  |  | **miR-1915-3p** | **miR-3656** | **miR-3665** | **miR-3960** | **miR-4497** | **miR-4787-5p** |  |
| miRNA expression microarray | plasma | 32 | yes | yes | yes | yes | yes | yes | this study |
| sRNA-seq | plasma | 2 | no | no | no | no | no | no | this study |
| sRNA-seq | serum | 5 | no | no | no | no | no | no | [1] |
| sRNA-seq | serum exosomes & supernatant | 3 | no | no | no | no | no | no | [2] |
| sRNA-seq | plasma | triplicates of 2 pools | yes/no | n.a. | n.a. | NA | n.a. | no/yes and NA | [3] |

sRNA-seq: small RNA sequencing

NA: not applicable, n.a.: not available

no: presumably false-negative detection

*GC content of > 82% (Supplementary Table S9)

REFERENCES

1. Burgos KL, Javaherian A, Bomprezzi R, Ghaffari L, Rhodes S, Courtright A, Tembe W, Kim S, Metpally R, Van Keuren-Jensen K: **Identification of extracellular miRNA in human cerebrospinal fluid by next-generation sequencing**. *RNA* 2013, **19**(5):712-722.

2. Yagi Y, Ohkubo T, Kawaji H, Machida A, Miyata H, Goda S, Roy S, Hayashizaki Y, Suzuki H, Yokota T: **Next-generation sequencing-based small RNA profiling of cerebrospinal fluid exosomes**. *Neurosci Lett* 2017, **636**:48-57.

3. Godoy PM, Barczak AJ, DeHoff P, Srinivasan S, Etheridge A, Galas D, Das S, Erle DJ, Laurent LC: **Comparison of Reproducibility, Accuracy, Sensitivity, and Specificity of miRNA Quantification Platforms**. *Cell Rep* 2019, **29**(12):4212-4222 e4215.
