# Supplementary material for: Identification of extremely GC-rich micro RNAs for RT-qPCR data normalization in human plasma
Source: Front Genet. 2023 Jan 4;13:1058668. doi: 10.3389/fgene.2022.1058668 (PMC9846067; doi:10.3389/fgene.2022.1058668)
Supplement: Supplementary file 1 [file DataSheet1.zip › Supporting information/Table_S5_Statistics_of_underrepresented_GC-extreme_miRNAs_in_sRNA-seq_data_of_human_blood_plasma.docx]

**Table S5 |** Underrepresentation of GC-extreme miRNAs in sRNA-seq data of human blood plasma.

| Pairwise comparison | Mean rank 1 | Mean rank 2 | Mean rank difference | *n*_1_ | *n*_2_ | *Z* | Adjusted *p* value* |
| --- | --- | --- | --- | --- | --- | --- | --- |
| miRNA microarray *versus* miRBase | 1851 | 1890 | -39.53 | 837 | 2654 | 0.93 | > 0.9999 |
| Small RNA-seq *versus* miRBase | 1535 | 1890 | -355.5 | 227 | 2654 | 4.79 | < 0.0001 |
| Small RNA-seq *versus* miRNA microarray | 1535 | 1851 | -315.9 | 227 | 837 | 3.94 | 0.0002 |

*Dunn's (1964) test of multiple comparisons following a significant Kruskal-Wallis test (calculated in GraphPad Prism; GraphPad Software, Inc., San Diego, CA, USA)

Cohort sizes: 12 (miRNA microarray), 2 (small-RNA transcriptome sequencing (sRNA-seq))

miRBase version: release 21

In red: significant mean rank difference

Reference

Dunn, OJ. Multiple comparisons using rank sums. *Technometrics* 6: 241–252 (1964)
