# Supplementary material for: Identification of extremely GC-rich micro RNAs for RT-qPCR data normalization in human plasma
Source: Front Genet. 2023 Jan 4;13:1058668. doi: 10.3389/fgene.2022.1058668 (PMC9846067; doi:10.3389/fgene.2022.1058668)
Supplement: Supplementary file 1 [file DataSheet1.zip › Supporting information/Table_S8_Microarray_normalization_approaches.docx]

**TABLE S8 |**Summary on approaches for microarray data normalization.

| **No.** | **Normalization approach** | **Abbreviation** | **Reference** | **Software** |
| --- | --- | --- | --- | --- |
| 1 | Global Mean Normalization | GMN | [1] | Microsoft^®^ Excel^®^ for Mac 2011 (version 14.7.3)^a^ |
| 2 | Generalized Procrustes Analysis | GPA | [2] | R script of [3] |
| 3 | Locally Weighted Polynomial Regression | loess | [4, 5] | “normalize.loess” function of R package affy [6] |
| 4 | Modified Loess Normalization | loessM | [7] | R script of [3] |
| 5 | Cyclic Loess Normalization | loessC | [8] | “normalizeCyclicLoess” function of limma R package [9] |
| 6 | Quantile Normalization | Quantile | [10] | Affymetrix^®^ Expression Console™ Software |
| 7 | Variance Stabilizing Normalization | VSN | [11] | “vsn” package from Bioconductor^b^ [12] |
| 8 | Invariants Normalization | VSN-INV | [13] | R script of [13] |
| 9 | Z-score Normalization | Z-score | [14] | Microsoft^®^ Excel^®^ for Mac 2011 (version 14.7.3)^a^ |

^a^Microsoft Corporation, Redmond, WA, USA

^b^<https://www.bioconductor.org/packages/devel/BiocViews.html#___Software>

R scripts: R version 3.0.3 (<https://www.r-project.org>)

References

1. Mestdagh P, Van Vlierberghe P, De Weer A, Muth D, Westermann F, Speleman F, Vandesompele J: **A novel and universal method for microRNA RT-qPCR data normalization**. *Genome Biol* 2009, **10**(6):ARTN R64.

2. Xiong HL, Zhang DP, Martyniuk CJ, Trudeau VL, Xia XH: **Using Generalized Procrustes Analysis (GPA) for normalization of cDNA microarray data**. *Bmc Bioinformatics* 2008, **9**.

3. Meyer SU, Kaiser S, Wagner C, Thirion C, Pfaffl MW: **Profound effect of profiling platform and normalization strategy on detection of differentially expressed microRNAs--a comparative study**. *PLoS One* 2012, **7**(6):e38946.

4. Cleveland WS, Devlin SJ: **Locally Weighted Regression - an Approach to Regression-Analysis by Local Fitting**. *Journal of the American Statistical Association* 1988, **83**(403):596-610.

5. Yang YH, Dudoit S, Luu P, Lin DM, Peng V, Ngai J, Speed TP: **Normalization for cDNA microarray data: a robust composite method addressing single and multiple slide systematic variation**. *Nucleic Acids Res* 2002, **30**(4):e15.

6. Gautier L, Cope L, Bolstad BM, Irizarry RA: **affy - analysis of Affymetrix GeneChip data at the probe level**. *Bioinformatics* 2004, **20**(3):307-315.

7. Risso D, Massa MS, Chiogna M, Romualdi C: **A modified LOESS normalization applied to microRNA arrays: a comparative evaluation**. *Bioinformatics* 2009, **25**(20):2685-2691.

8. Bolstad BM, Irizarry RA, Astrand M, Speed TP: **A comparison of normalization methods for high density oligonucleotide array data based on variance and bias**. *Bioinformatics* 2003, **19**(2):185-193.

9. Smyth GK: **limma: Linear Models for Microarray Data**. In: *Bioinformatics and Computational Biology Solutions Using R and Bioconductor.* Edited by Gentleman R, Carey V, Huber W, Irizarry R, Dudoit S: Springer New York; 2005: 397-420.

10. Wang B, Xi Y: **Challenges for MicroRNA Microarray Data Analysis**. *Microarrays (Basel)* 2013, **2**(2).

11. Huber W, von Heydebreck A, Sultmann H, Poustka A, Vingron M: **Variance stabilization applied to microarray data calibration and to the quantification of differential expression**. *Bioinformatics* 2002, **18 Suppl 1**:S96-104.

12. Huber W, von Heydebreck A, Sueltmann H, Poustka A, Vingron M: **Parameter estimation for the calibration and variance stabilization of microarray data**. *Stat Appl Genet Mol Biol* 2003, **2**:Article3.

13. Pradervand S, Weber J, Thomas J, Bueno M, Wirapati P, Lefort K, Dotto GP, Harshman K: **Impact of normalization on miRNA microarray expression profiling**. *RNA* 2009, **15**(3):493-501.

14. Cheadle C, Vawter MP, Freed WJ, Becker KG: **Analysis of microarray data using Z score transformation**. *Journal of Molecular Diagnostics* 2003, **5**(2):73-81.
