# Supplementary material for: Identification of extremely GC-rich micro RNAs for RT-qPCR data normalization in human plasma
Source: Front Genet. 2023 Jan 4;13:1058668. doi: 10.3389/fgene.2022.1058668 (PMC9846067; doi:10.3389/fgene.2022.1058668)
Supplement: Supplementary file 1 [file DataSheet1.zip › Supporting information/Table_S9_Stable_miRNAs_selected_by_microarray_expression_analysis.docx]

**TABLE S9 |** Most stable plasma miRNAs selected by microarray-based technology & two established miRNA normalizers.

| **Mature miRNA** | **MiRBase accession number** | **Chromo-somal location** | **miRNA subtype (host gene)** | **GC content (%)** | ***MFE*** | **EV carrier in blood** | **Conserved in *n* other species** | **Rank** | **Detection technique** |
| --- | --- | --- | --- | --- | --- | --- | --- | --- | --- |
| miR-93-5p | MIMAT0000093 | 7 | intronic | 52.7 | –3.4 | exosomes & MVs | 161 | est. | Array [1], qPCR & sRNA-seq (this study) |
| miR-126-3p | MIMAT0000445 | 9 | intronic | 45.5 | –0.7 | exosomes & MVs | 200 | est. | Array [2], qPCR & sRNA-seq (this study) |
| miR-185-5p | MIMAT0000455 | 22 | intronic | 50.0 | –1.3 | exosomes & MVs | 133 | 14 | Array, qPCR & sRNA-seq (this study) |
| miR-320d | MIMAT0006764 | 13, X | intergenic | 47.4 | 0.0 | exosomes & MVs | *Pan troglodytes, Pongo pygmaeus* | 6 | Array & qPCR [1, 3] |
| miR-425-5p | MIMAT0003393 | 3 (49,020,199 to 49,020,221) | intronic | 47.8 | –0.7 | exosomes & MVs | 120 | 4 | Array, qPCR [4] & sRNA-seq [5] |
| miR-486-5p | MIMAT0002177 | 8 | intergenic | 63.6 | 0.0 | exosomes & MVs | 37 | 9 | Array & qPCR [6]; qPCR [7, 8] |
| miR-1469 | MIMAT0007347 | 15 | intronic/other | 90.9 | –7.4 | exosomes | *Oikopleura dioica, Pongo pygmaeus* | 2 | Array [9] |
| miR-1915-3p | MIMAT0007892 | 10 | intronic | 90.0 | –4.1 | exosomes (serum [10]) | *Pongo pygmaeus* | 5 | Array & qPCR [11] |
| miR-3656 | MIMAT0018076 | 11 | intronic/ exonic | 88.2 | –0.9 | exosomes | no information | 7 | Array [12]; Array & qPCR [13] |
| miR-3665 | MIMAT0018087 | 13 | intergenic | 83.3 | –1.9 | exosomes (serum [10]) | no information | 2 | Array [14] |
| miR-3960-5p | MIMAT0019337 | 9 | other (*CDK9*) | 95.0 | –1.8 | EVs [15] & exosomes | *Mus musculus* | 5 | Array & qPCR [16] |
| miR-4488 | MIMAT0019022 | 11 | intergenic | 88.9 | –4.8 | exosomes | *Gorilla gorilla* | 1 | sRNA-seq [17] |
| miR-4497 | MIMAT0019032 | 12 | intergenic | 82.4 | –5.0 | exosomes & MVs | no information | 14 | sRNA-seq [18] |
| miR-4787-5p | MIMAT0019956 | 3 (50,675,093 to 50,675,114) | intergenic | 86.4 | –8.0 | exosomes (serum [19]) | no information | 2 | Array [20, 21] |

In blue font: established miRNA normalizers selected from literature.

*MFE*: minimal free energy of folding (kcal/mol) at 37°C calculated by the RNAFold server (<http://rna.tbi.univie.ac.at/cgi-bin/RNAWebSuite/RNAfold.cgi>) with default settings.

Stability ranks: aggregated ranks of several procedures for microarray normalisation; MIMAT: unique chronological accession number of miRBase for a mature miRNA; EV: extracellular vesicle; MV: microvesicle; sRNA-seq: small RNA sequencing; Array: miRNA expression microarray;

Intragenic miRNAs: intragenic microRNA database termed miRIAD ([22]; <https://www.miriad-database.org>);

Blood shuttles: EVmiRNA ([23]; <http://bioinfo.life.hust.edu.cn/EVmiRNA/#!/>), Vesiclepedia ([24]; <http://microvesicles.org/index.html>), or the peer-reviewed literature;

Genomic location and miRNA orthologues: RNAcentral release 19 (<https://rnacentral.org>) and miRBase version 22 (<https://www.mirbase.org>).

REFERENCES

1. Liu X, Zhang L, Cheng K, Wang X, Ren G, Xie P: **Identification of suitable plasma-based reference genes for miRNAome analysis of major depressive disorder**. *J Affect Disord* 2014, **163**:133-139.

2. Matsuzaki K, Fujita K, Tomiyama E, Hatano K, Hayashi Y, Wang C, Ishizuya Y, Yamamoto Y, Hayashi T, Kato T *et al*: **MiR-30b-3p and miR-126-3p of urinary extracellular vesicles could be new biomarkers for prostate cancer**. *Transl Androl Urol* 2021, **10**(4).

3. Wang WH, Sun G, Zhang LY, Shi L, Zeng YJ: **Circulating MicroRNAs as Novel Potential Biomarkers for Early Diagnosis of Acute Stroke in Humans**. *Journal of Stroke & Cerebrovascular Diseases* 2014, **23**(10):2607-2613.

4. Li L, Chen HZ, Chen FF, Li F, Wang M, Wang L, Li YQ, Gao DS: **Global microRNA expression profiling reveals differential expression of target genes in 6-hydroxydopamine-injured MN9D cells**. *Neuromolecular Med* 2013, **15**(3):593-604.

5. Vanhie A, Peterse D, Beckers A, Cuellar A, Fassbender A, Meuleman C, Mestdagh P, D'Hooghe T: **Plasma miRNAs as biomarkers for endometriosis**. *Hum Reprod* 2019, **34**(9):1650-1660.

6. Boeri M, Verri C, Conte D, Roz L, Modena P, Facchinetti F, Calabro E, Croce CM, Pastorino U, Sozzi G: **MicroRNA signatures in tissues and plasma predict development and prognosis of computed tomography detected lung cancer**. *Proc Natl Acad Sci U S A* 2011, **108**(9):3713-3718.

7. Shen J, Liu Z, Todd NW, Zhang H, Liao J, Yu L, Guarnera MA, Li R, Cai L, Zhan M *et al*: **Diagnosis of lung cancer in individuals with solitary pulmonary nodules by plasma microRNA biomarkers**. *BMC Cancer* 2011, **11**:374.

8. Prats-Puig A, Ortega FJ, Mercader JM, Moreno-Navarrete JM, Moreno M, Bonet N, Ricart W, Lopez-Bermejo A, Fernandez-Real JM: **Changes in circulating microRNAs are associated with childhood obesity**. *J Clin Endocrinol Metab* 2013, **98**(10):E1655-1660.

9. Ali S, Almhanna K, Chen W, Philip PA, Sarkar FH: **Differentially expressed miRNAs in the plasma may provide a molecular signature for aggressive pancreatic cancer**. *American Journal of Translational Research* 2011, **3**(1):28-47.

10. Lim MK, Yoo J, Sheen DH, Ihm C, Lee SK, Kim SA: **Serum Exosomal miRNA-1915-3p Is Correlated With Disease Activity of Korean Rheumatoid Arthritis**. *In Vivo* 2020, **34**(5):2941-2945.

11. Ding L, Liu J, Shen HX, Pan LP, Liu QD, Zhang HD, Han L, Shuai LG, Ding EM, Zhao QN *et al*: **Analysis of plasma microRNA expression profiles in male textile workers with noise-induced hearing loss**. *Hear Res* 2015.

12. Qu XY, Zhao M, Wu S, Yu WJ, Xu JR, Xu J, Li JY, Chen LJ: **Circulating microRNA 483-5p as a novel biomarker for diagnosis survival prediction in multiple myeloma**. *Medical Oncology* 2014, **31**(10):ARTN 219.

13. Matamala N, Vargas MT, Gonzalez-Campora R, Minambres R, Arias JI, Menendez P, Andres-Leon E, Gomez-Lopez G, Yanowsky K, Calvete-Candenas J *et al*: **Tumor MicroRNA Expression Profiling Identifies Circulating MicroRNAs for Early Breast Cancer Detection**. *Clinical Chemistry* 2015, **61**(8):1098-1106.

14. Jin H, Li C, Ge H, Jiang Y, Li Y: **Circulating microRNA: a novel potential biomarker for early diagnosis of intracranial aneurysm rupture a case control study**. *J Transl Med* 2013, **11**:296.

15. Hu Y, Xu R, Chen CY, Rao SS, Xia K, Huang J, Yin H, Wang ZX, Cao J, Liu ZZ *et al*: **Extracellular vesicles from human umbilical cord blood ameliorate bone loss in senile osteoporotic mice**. *Metabolism* 2019, **95**:93-101.

16. Wang L, Chen YJ, Xu K, Xu H, Shen XZ, Tu RQ: **Circulating microRNAs as a Fingerprint for Endometrial Endometrioid Adenocarcinoma**. *Plos One* 2014, **9**(10):ARTN e110767.

17. Huang X, Yuan T, Tschannen M, Sun Z, Jacob H, Du M, Liang M, Dittmar RL, Liu Y, Liang M *et al*: **Characterization of human plasma-derived exosomal RNAs by deep sequencing**. *Bmc Genomics* 2013, **14**:319.

18. Mirza AH, Kaur S, Nielsen LB, Storling J, Yarani R, Roursgaard M, Mathiesen ER, Damm P, Svare J, Mortensen HB *et al*: **Breast Milk-Derived Extracellular Vesicles Enriched in Exosomes From Mothers With Type 1 Diabetes Contain Aberrant Levels of microRNAs**. *Front Immunol* 2019, **10**:2543.

19. Yan SS, Han B, Gao SY, Wang XC, Wang ZF, Wang FK, Zhang JJ, Xu DH, Sun BC: **Exosome-encapsulated microRNAs as circulating biomarkers for colorectal cancer**. *Oncotarget* 2017, **8**(36):60149-60158.

20. Pu Q, Huang Y, Lu Y, Peng Y, Zhang J, Feng G, Wang C, Liu L, Dai Y: **Tissue-specific and plasma microRNA profiles could be promising biomarkers of histological classification and TNM stage in non-small cell lung cancer**. *Thoracic Cancer* 2016.

21. Li Y, Li P, Yu S, Zhang J, Wang T, Jia G: **miR-3940-5p associated with genetic damage in workers exposed to hexavalent chromium**. *Toxicol Lett* 2014, **229**(1):319-326.

22. Hinske LC, Franca GS, Torres HA, Ohara DT, Lopes-Ramos CM, Heyn J, Reis LF, Ohno-Machado L, Kreth S, Galante PA: **miRIAD-integrating microRNA inter- and intragenic data**. *Database (Oxford)* 2014, **2014**.

23. Liu T, Zhang Q, Zhang J, Li C, Miao YR, Lei Q, Li Q, Guo AY: **EVmiRNA: a database of miRNA profiling in extracellular vesicles**. *Nucleic Acids Res* 2019, **47**(D1):D89-D93.

24. Pathan M, Fonseka P, Chitti SV, Kang T, Sanwlani R, Van Deun J, Hendrix A, Mathivanan S: **Vesiclepedia 2019: a compendium of RNA, proteins, lipids and metabolites in extracellular vesicles**. *Nucleic Acids Res* 2019, **47**(D1):D516-D519.
